# Redox-Active Metal–Organic Framework Nanocrystals for the Simultaneous Adsorption, Detection, and Detoxification of Heavy Metal Cations

**DOI:** 10.1021/acsami.5c18562

**Published:** 2025-12-22

**Authors:** Patrick Damacet, Elissa O. Shehayeb, Susanna Monti, Giovanni Barcaro, Katherine A. Mirica

**Affiliations:** † Department of Chemistry, Burke Laboratory, 3728 Dartmouth College, Hanover, New Hampshire 03755, United States; ‡ CNR-ICCOM, Institute of Chemistry of Organometallic Compounds, Area Della Ricerca, Pisa I56124, Italy; § CNR-IPCF, Institute for Chemical and Physical Processes, Area Della Ricerca, Pisa I56124, Italy

**Keywords:** water remediation, metal−organic frameworks, heavy metal ions, detoxification, redox-active frameworks, heavy metal detection, electronic textiles, material–analyte interactions

## Abstract

The widespread contamination of water by heavy metals requires materials capable of efficient capture, in situ detoxification, and real-time monitoring. This work examines a series of redox-active metal–organic frameworks (MOFs) constructed from hexahydroxytriphenylene (HHTP) ligands coordinated to cobalt, nickel, and copper (Co-HHTP, Ni-HHTP, and Cu-HHTP), revealing how framework architecture and metal coordination environment dictate adsorption capacity, redox activity, and detection performance toward cadmium (Cd^2+^), mercury (Hg^2+^), and lead (Pb^2+^) ions. Among the series, Co-HHTP exhibits the highest uptake capacities of 169, 733, and 554 mg g^–1^ for Cd^2+^, Hg^2+^, and Pb^2+^, respectively, attributed to its trigonal stacking and intercalated layers that expose labile water-capped metal sites. These sites facilitate electron transfer, enabling a redox-active capture pathway in which heavy metal cations are partially reduced, with concurrent oxidation of the HHTP ligand. In contrast, Cu-HHTP, with an eclipsed stacking arrangement and limited redox complementarity to the heavy metal ions examined, remains redox-inert and exhibits the lowest performance. Deposition of Co-HHTP onto cotton, silk, and polyester yields MOF@textile composites that retain adsorption efficiency and enable rapid detection of heavy metals at low-ppm concentrations. These findings establish a structure–function correlation, emphasizing how stacking configuration, metal accessibility, and redox-active ligands collectively govern multimechanistic heavy metal remediation.

## Graphical abstract

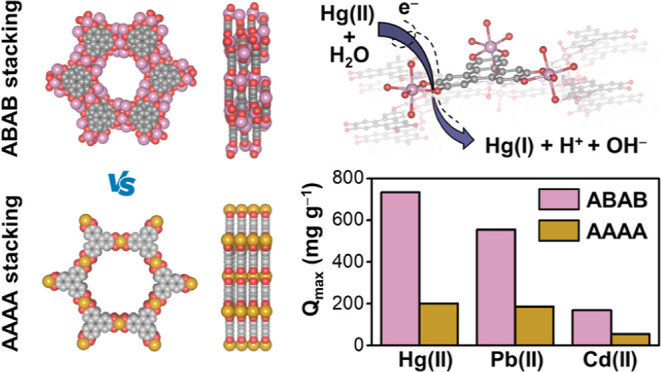

## 1 Introduction

The widespread contamination of water sources by heavy metal pollutants, driven by the extensive exploitation of natural resources, intensified human activities, and rapid industrialization processes, poses a significant threat to global health.^1,2^ Heavy metal pollutants leach into aquatic systems through both anthropogenic sources, such as mining, smelting, and urban runoff, and natural sources, including rock weathering, volcanic eruptions, and atmospheric deposition.^3,4^ Due to their highly toxic, bioaccumulative, and carcinogenic properties,^5^ heavy metals represent one of the most critical classes of pollutants.^6^ Efforts to address these threats on a global scale have led to the establishment of transboundary treaties, such as the Minamata Convention on Mercury,^7^ the Aarhus Protocol on Heavy Metals,^8^ and the Basel Convention.^9^ However, the effectiveness of such policies hinges on parallel advancements in technologies capable of rapidly, sensitively, and cost-effectively removing and detecting heavy metals in water systems.^10^ Despite the adoption of conventional water treatment techniques over time, such as reverse osmosis,^11^ chemical precipitation,^12^ ion exchange,^13^ coagulation,^14^ electrodialysis,^15^ and membrane filtration,^16^ these techniques often suffer from inherent limitations, including poor filtration capacity, high operational costs, low sensitivity, and monofunctionality. In response, adsorptive water decontamination using multifunctional porous materials has emerged as a promising alternative in water treatment applications, offering the potential for high uptake capacities and integrated sensing capabilities for heavy metal ions.^17,18^

Metal–organic frameworks (MOFs) represent an emerging class of porous, crystalline materials with high surface areas, tunable pore structures, and chemically versatile architectures.^19^ These properties have positioned MOFs as promising candidates for water purification,^20^ with demonstrated efficacy in removing heavy metal ions,^21,22^ organic pollutants,^23^ and radioactive waste.^24^ Nonetheless, the majority of conventional MOFs used in water remediation suffer from two primary limitations that restrict their broader utility. First, their lack of intrinsic redox activity restricts electron-transfer-based interactions with contaminants, limiting them to physisorption or coordination-driven pathways, resulting in modest adsorption capacities.^25^ While postsynthetic modification strategies have introduced redox-active functionalities,^26,27^ such approaches are often time-intensive, costly, and difficult to scale. Second, their electrically insulating nature limits their multifunctionality, hindering the integration of capture, detoxification, and detection within a single material platform.^28^ Two-dimensional (2D) electrically conductive MOFs (cMOFs) have emerged as a promising subclass capable of overcoming these limitations.^29,30^ Their combination of intrinsic conductivity, redox-activity, accessible metal sites, and tunable layered architectures provide a rich platform for achieving synergistic adsorption, detoxification, and detection.^31^ In our previous work, we demonstrated the first use of triphenylene-based cMOFs derived from hexaiminotriphenylene (HITP) and hexahydroxytriphenylene (HHTP) for the dual capture and detection of dichromate (Cr­(VI)) and permanganate (Mn­(VII)) oxyanions in water.^32^ These cMOFs exhibited exceptional uptake capacities (up to 827 mg g^–1^) and revealed valuable insights into material–analyte interactions, involving electrostatic forces, hydrogen bonding, and chemisorption. However, the previous study focused exclusively on negatively charged oxyanions, leaving the mechanisms governing interactions with cationic species, such as toxic heavy metal ions, largely unexplored in this subclass of MOFs. As such, several key gaps remain unexamined, including (i) the contribution of redox activity to detoxification and redox-coupled adsorption, (ii) the influence of framework stacking configuration on the density and accessibility of adsorption sites, and (iii) the mediating role of metal node identity on selective ion capture. Addressing these gaps is critical for establishing mechanistic design principles and could unlock new strategies for engineering multifunctional cMOFs capable of both efficient heavy-metal removal and sensing in aqueous environments.

Herein, we present a systematic investigation into how metal nodes, redox-active sites, and stacking arrangements in a series of M-HHTP cMOFs linked with cobalt, nickel, and copper govern their ability to capture, reduce, and detect cadmium (Cd^2+^), mercury (Hg^2+^), and lead (Pb^2+^) ions in water. Co-HHTP, which crystallizes in a trigonal layered architecture of 2D sheets interleaved with 0D water-capped cobalt–catechol complexes, exhibits the highest adsorption capacities and fastest kinetics among the tested materials, achieving 169, 733, and 554 mg g^–1^ for Cd^2+^, Hg^2+^, and Pb^2+^, respectively. Ni-HHTP, its isostructural analogue, shows moderately lower performance, while Cu-HHTP, which adopts an eclipsed stacking configuration, exhibits the lowest uptake. This reduced performance is attributed to the absence of exposed catechol oxygens from 0D complexes, which in Co- and Ni-HHTP provide interaction sites for heavy metal binding. Across all frameworks, a consistent adsorption trend of Hg^2+^ > Pb^2+^ > Cd^2+^ is observed, correlating with the standard reduction potentials (*E*°) of these ions. X-ray photoelectron spectroscopy (XPS) confirms partial reduction of Hg­(II) to Hg­(I) and Pb­(II) to Pb(0) in Co- and Ni-HHTP, coinciding with oxidation of the redox-active, electron-donating HHTP ligand. In contrast, Cd­(II) remains chemically unaltered and shows limited adsorption on all MOFs, underscoring the role of redox-matching between the contaminant ions and framework. Complementary spectroscopic and molecular modeling analyses reveal that uptake arises from the interplay of redox reactions, chemisorption, and electrostatic interactions. Deposition of Co-HHTP onto cotton, silk, and polyester fabrics generates MOF@textile composites that retain bulk adsorption efficiency and enable rapid detection of heavy metals at low-ppm concentrations. Collectively, these findings establish a structure–function correlation in redox-active cMOFs, demonstrating how stacking motifs and water-capped trinuclear complexes cooperatively dictate heavy metal capture and transformation in aqueous environments.

## 2 Experimental Design

### 2.1 Choice of MOF Materials

We selected M-HHTP MOFs with cobalt, nickel, and copper nodes for the dual capture and detection of heavy metal cations for four major reasons. First, these cMOFs possess negatively charged surfaces in water. This property is expected to favor electrostatic interactions with cationic species, thereby enhancing adsorption efficiency and selectivity.^33^ Second, the structural characteristics of these MOFs are closely tied to the identity of their metal nodes. Co- and Ni-HHTP crystallize in a trigonal phase, comprising alternating layers of 2D honeycomb sheets and intercalated trinuclear 0D M_3_(HHTP)­(H_2_O)_12_ clusters rotated 60° relative to the hexagonal lattice,.^34^ In contrast, Cu-HHTP adopts an eclipsed, C-centered monoclinic crystal structure (Figure 1). Studying how different metal nodes and stacking structures influence adsorption performance provides valuable insights into the structure–property relationships of these materials. Third, these MOFs offer at least four combinations of structural features advantageous for adsorption applications: (i) accessible metal sites, (ii) aligned porous channels, (iii) redox-active ligands, and (iv) oxygen-rich capping sites. These features create multiple adsorption pathways, including electrostatic attraction, redox-mediated capture, and chemisorption, that enhance efficacy toward heavy metals.^27,35^ Fourth, the synthetic precursors of these MOFs are readily accessible, offering a cost-effective and practical solution for water remediation.

**Figure 1:**
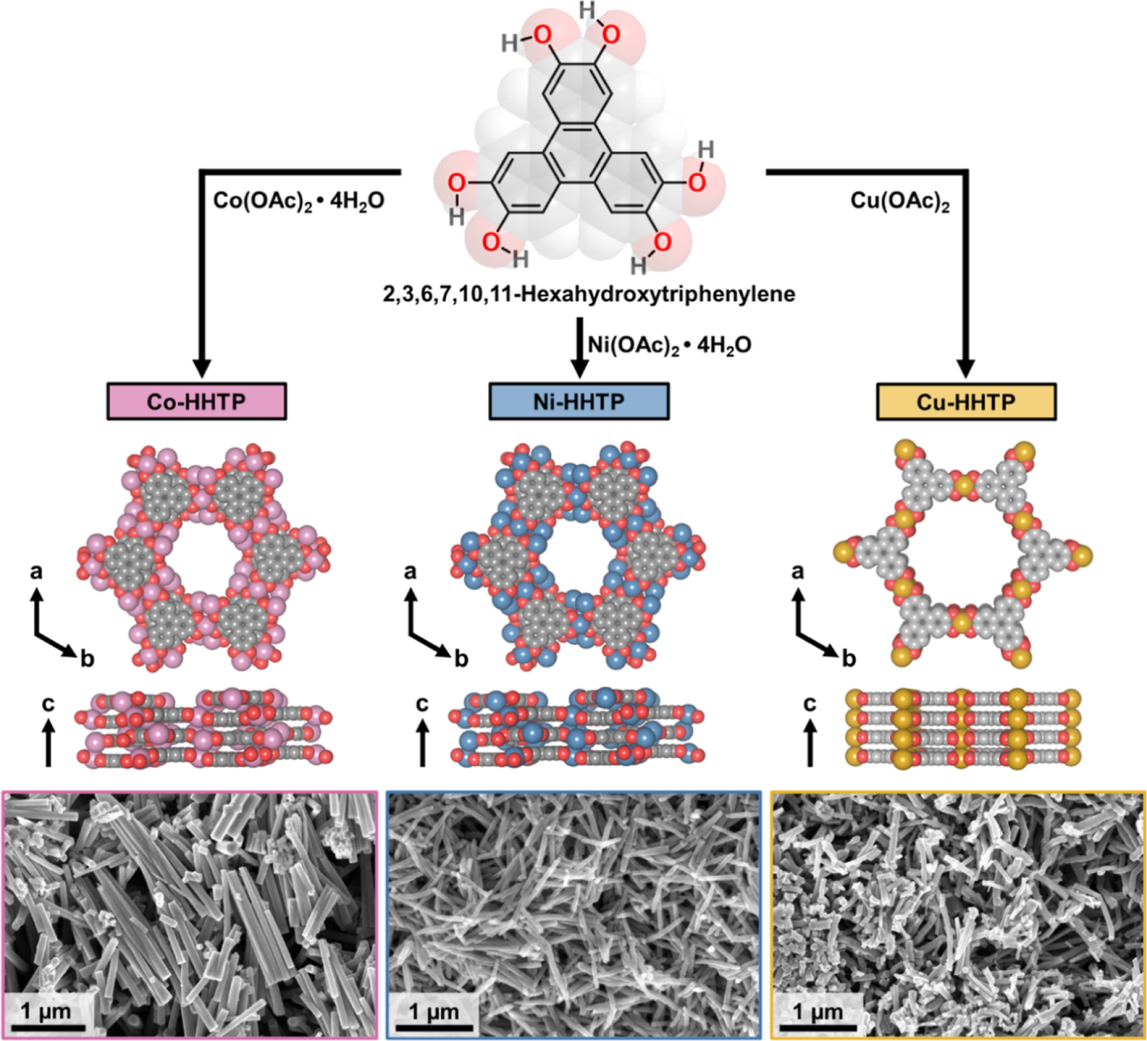
Synthetic illustration and crystal structures of the M-HHTP MOFs (M = Co, Ni, and Cu) employed in this study. The scanning electron microscopy images depict the nanorod-like morphology of the synthesized materials.

### 2.2 Choice of Heavy Metal Pollutants

Cd­(II), Hg­(II), and Pb­(II) rank among the most toxic heavy metal contaminants to humans and the environment, making them high-priority targets for environmental remediation and public health intervention.^36^ We selected these representative divalent cations for four main reasons. First, they exhibit potent neurotoxicity and cardiotoxicity, with well-documented links to impaired neurological development, disrupted cellular processes, and irreversible brain damage.^37^ Second, these metals are prone to bioaccumulation and possess long biological half-lives, up to 38 years for Cd­(II),^38^ 3 years for Pb­(II),^39^ and 50 days for Hg­(II),^40^ resulting in persistent toxic effects following chronic exposure. Third, recent cases of contamination have highlighted their continued presence in drinking water sources serving homes and schools worldwide, with reports from the US,^41^ Belgium,^42^ and Brazil.^43^ The global prevalence of these pollutants calls for urgent, cost-effective, and efficient remediation technologies. Fourth, establishing a detailed mechanism of interaction between 2D cMOFs and these heavy metal pollutants enables future design of hierarchical materials with tailored properties for heavy metal decontamination from water.

## 3 Materials and Methods

### 3.1 Synthesis Procedures

#### 3.1.1 Co-HHTP

32 mg of HHTP (0.1 mmol, 1 equiv) was added to 0.8 mL of 1,3-Dimethyl-2-imidazolidinone in a 20 mL glass vial and was sonicated for 15 min. Seventy five mg of Co­(OAc)_2_.4H_2_O (0.3 mmol, 3 equiv) dissolved in 4.8 mL of DI water were added dropwise to the ligand solution over a period of 2 min. The resulting mixture was shaken for a few seconds before being placed on a preheated hot plate at 85 °C and left overnight without stirring while the vial was loosely caped. The resulting black powder was filtered, washed with DI water (30 mL), methanol (25 mL) ethanol (25 mL), and acetone (25 mL), before being dried in a vacuum oven set at 75 °C for 32 h. Reaction yield: 45%.

#### 3.1.2 Ni-HHTP

Ni-HHTP was prepared according to a previously reported procedure with some modifications.^35^ In brief, 20 mg of Ni­(OAc)_2_.4H_2_O (0.08 mmol, 2 equiv) was dissolved in 15 mL DI water in a 20 mL scintillation vial. Thirteen mg of 2,3,6,7,10,11-hexahydroxytriphenylene (0.04 mmol, 1 equiv) was added to the aqueous solution, which was then left to sonicate for 15 min. The resulting mixture was loosely capped and heated without stirring on a hot plate set at 85 °C for 14 h. The resulting black powder was filtered, washed with DI water (20 mL), ethanol (40 mL), and acetone (40 mL), before being dried in a vacuum oven set at 75 °C for 32 h. Reaction yield: 67%.

#### 3.1.3 Cu-HHTP

Ten mg of anhydrous copper acetate (0.055 mmol, 1 equiv) was dissolved in 3 mL of DI water and sonicated for 5 min. Eighteen mg of HHTP (0.055 mmol, 1 equiv) was added to the copper solution and the resulting mixture was sonicated for 10 min to allow for a homogeneous suspension. 0.3 mL of DMF was then added dropwise and the resulting solution was heated without stirring on a hot plate set at 70 °C for 3 h while left exposed to air. The resulting black powder was filtered, washed with DI water (30 mL), ethanol (30 mL), and acetone (30 mL), before being dried in a vacuum oven set at 75 °C for 24 h. Reaction yield: 91%.

### 3.2 Activation of MOFs

All MOFs were activated following the same procedure. Briefly, the MOF crystals were soaked in ethanol for 2 days, with the solvent being exchanged with fresh ethanol every 12 h. The solvent was then exchanged with acetone following the same process. The resulting crystals were finally dried for 48 h in a vacuum oven set at 75 °C prior to structural and morphological characterization.

### 3.3 Batch Adsorption Studies

Stock solutions of 500 ppm of Cd­(II), Hg­(II), and Pb­(II) were first prepared by dissolving appropriate amounts of CdCl_2_, HgCl_2_, and Pb­(NO_3_)_2_ powders in deionized (DI) water. To achieve varying initial concentrations of heavy metals (10–500 ppm), these stock solutions were diluted with DI water. Adsorption experiments were conducted in 8 mL glass scintillation vials, where 2 mg of activated MOF adsorbent was added to 3 mL of heavy metal solution at the desired concentration. The mixtures were stirred at room temperature for 4 h, after which the MOF powders were separated from the solutions using 0.45 μm PTFE syringe filters. The supernatants were analyzed using inductively coupled plasma optical emission spectrometry (ICP–OES) to quantify the remaining concentrations of heavy metals in solution postadsorption. More information can be found in Section S3 of the Supporting Information.

## 4 Results and Discussion

### 4.1 Morphology and Structure of M-HHTP MOFs

In our effort to accurately assess the impact of metal node identity, redox activity, and stacking arrangement on the adsorption properties of M-HHTP MOFs, we strategically controlled experimental variables that could otherwise confound a direct comparison. We generated Co-, Ni-, and Cu-HHTP using metal salts with acetate counterions, thereby ensuring consistency in counterions and minimizing differences in surface charge, a factor previously shown to influence adsorption performance.^44^ To add, we followed established protocols to yield nanorod-like crystals with comparable morphologies and aspect ratios (5.1–8.7), thus reducing size- and shape-dependent variability in uptake of adsorbents (Figures S1–S4). We further activated the MOFs under similar procedures and performed batch adsorption studies in parallel to ensure consistent adsorbent–adsorbate contact times and ambient temperatures. We comprehensively characterized the structural, morphological, and electronic properties of the MOFs using powder X-ray diffraction (pXRD), scanning electron microscopy coupled to energy dispersive X-ray spectroscopy (SEM–EDX), transmission electron microscopy (TEM), attenuated total reflectance infrared spectroscopy (ATR-IR), Brunauer–Emmett–Teller (BET) surface area analysis, thermogravimetric analysis (TGA), and four-point probe measurements (Figures S5–S22). These analyses confirmed that all M-HHTP materials were crystalline, porous, thermally stable, and structurally consistent with literature-reported analogs.^45^

### 4.2 Adsorption Isotherms

We started our investigations by examining the adsorption capabilities of the three HHTP-MOFs for heavy metal cations across a range of initial concentrations. We varied the concentrations of Cd^2+^, Hg^2+^, and Pb^2+^ between 10 and 500 ppm, and estimated the experimental uptake capacities of the suite of HHTP-MOFs after 4 h of exposure to contaminants using eq 1

1 $$Q_{e}=\frac{C_{0}-C_{e}}{m}\times V$$

In this equation, *Q*_e_ represents the adsorption capacity, expressed as milligrams of contaminant adsorbed per gram of MOF (mg g^–1^), *C*_0_ and *C*_e_ (in ppm) refer to the concentrations of the contaminants in solution before and after adsorption, respectively, *m* refers to the mass of adsorbent (in mg), and *V* refers to the total volume of the solution (in mL).

The adsorption uptake for all MOFs, as illustrated in the isotherms of Figure 2a, showed a sharp increase with rising concentrations of heavy metals, before eventually plateauing as the adsorptive sites within the frameworks became saturated. We found that the adsorption capacities are influenced by both, the identity of the metal contaminant, and the structural characteristics of the MOF adsorbents. While all MOFs demonstrated a markedly higher *Q*_e_ for Hg­(II) and Pb­(II) compared to Cd­(II), likely due to the higher standard reduction potentials (*E*°) of the former, which thermodynamically favor redox-mediated interactions,^46^ Co-HHTP consistently exhibited superior adsorption performance relative to Ni-HHTP and Cu-HHTP for all tested metal cations. We hypothesized this trend, following the order of Co-HHTP > Ni-HHTP > Cu-HHTP, to be attributed to a combination of factors, including the (i) stacking configuration, (ii) redox activity, and (iii) intrinsic properties, such as BET surface area and surface charge of the frameworks. Both Co-HHTP and Ni-HHTP crystallize in a bilayered structure composed of intercalated layers of trinuclear M_9_(HHTP)_4_ complexes per unit cell, resulting in a high density of metal-catecholate coordination sites capped with aqua ligands.^47^ These oxygen-rich sites act as nucleophilic centers capable of engaging in charge transfer with electrophilic heavy metal cations, thereby, enabling a redox-coupled adsorption mechanism^48,49^ (more on this later). In contrast, Cu-HHTP adopts an eclipsed AA-stacked layered structure with a lower 3:2 Cu: HHTP stoichiometry, which lacks the intercalated clusters and offers fewer accessible adsorptive capping sites. This structural limitation results in a reduced density of redox-active surface sites and diminished capacity for redox-mediated interactions, thereby leading to inferior adsorption performance.

**Figure 2:**
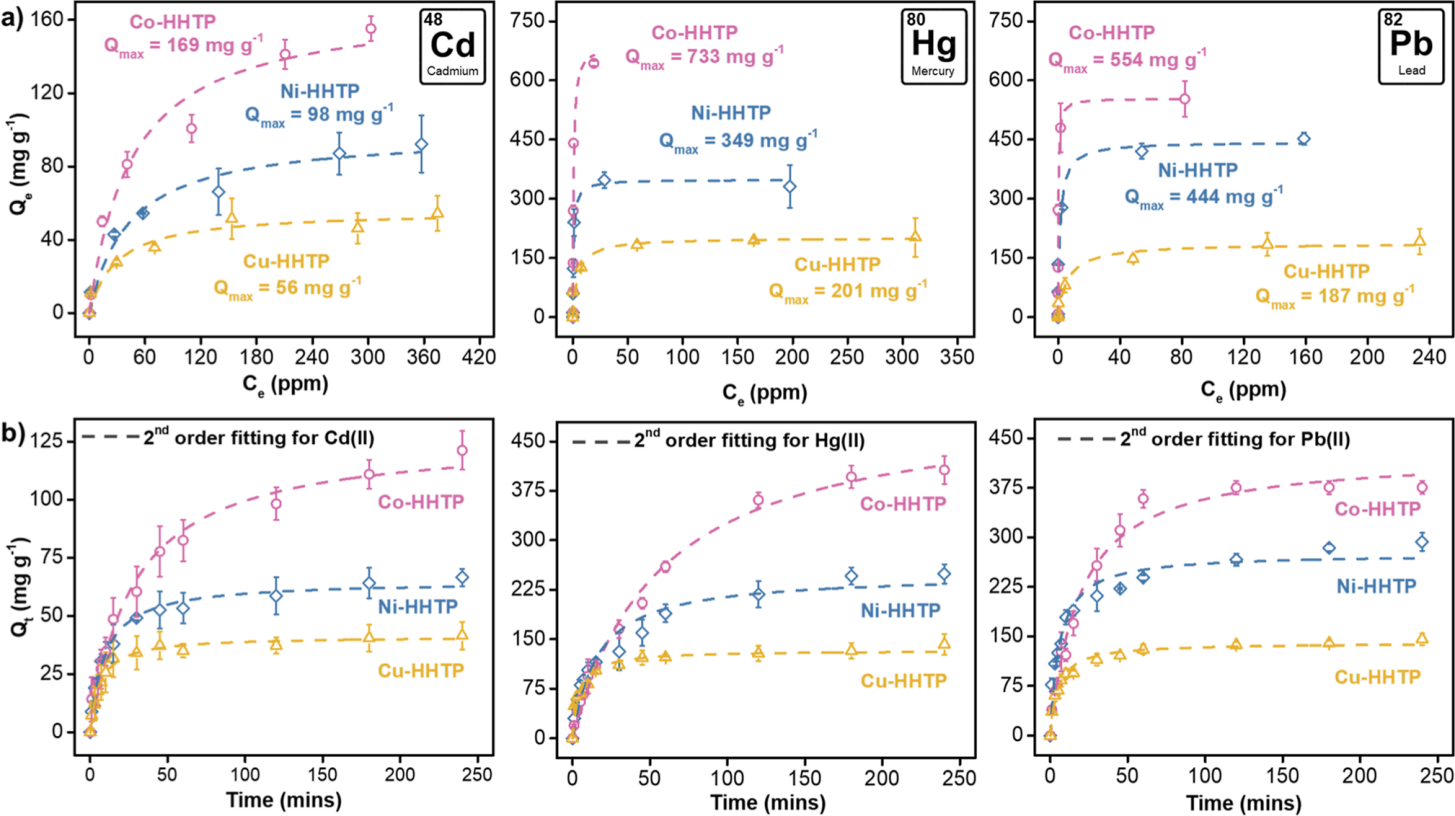
(a) Langmuir model fitting and (b) pseudo-second order fitting for the adsorption isotherms of Cd­(II), Hg­(II), and Pb­(II) heavy metals over M-HHTP adsorbents. The data points correspond to experimental adsorption data whereas the lines correspond to model fitting results. Error bars represent standard deviation from the mean value of three independent experiments. Conditions: *m*_MOF_ = 2 mg, *V*_Solution_ = 3 mL, *T* = 298 K. Note the initial concentration of the heavy metals in the kinetic experiments is 250 ppm.

To gain insights into the mode of adsorption, we fitted the experimental data to four equilibrium models,^50^ namely Langmuir, Freundlich, Temkin, and Dubinin–Radushkevich (D-R), as detailed in Section S3.1. Among these, the Langmuir model provided the best fit for heavy metal adsorption across most cMOFs, as evidenced by higher correlation coefficients (0.97–0.99) obtained from least-squares regression, compared to the other models, which yielded values as low as 0.48 (Figures S23–S31). This fitting profile suggested that (i) the adsorption sites on the surface of MOFs are energetically and structurally uniform, (ii) adsorption proceeds via monolayer coverage with a finite number of binding sites, and (iii) no adsorbate–adsorbate interactions are present, consistent with spatially discrete adsorption domains.^22^ Langmuir-derived maximum adsorption capacities (*Q*_max_) confirmed Co-HHTP as the highest-performing material, achieving capacities of 169, 733, and 554 mg g^–1^ for Cd^2+^, Hg^2+^, and Pb^2+^, respectively. These values are comparable to, or in some cases surpass, those reported for state-of-the-art MOF-based adsorbents (Figure S32). In contrast, Cu-HHTP displayed the lowest *Q*_max_ values of 56 mg g^–1^, 201 mg g^–1^, and 187 mg g^–1^ of Cd^2+^, Hg^2+^, and Pb^2+^ respectively.

To further elucidate the role of stacking configuration and aqua-capped intercalated complexes, we synthesized, characterized, and tested an analogous cobalt-based cMOF incorporating cobalt bis­(diimine) linkages, referred to as Co-HITP (HITP = 2,3,6,7,10,11-hexaiminotriphenylene) (Figures S33–S39). Unlike Co-HHTP, Co-HITP adopts a slipped-parallel stacking with square-planar Co^2+^ centers and lacks both intercalated layers and the trinuclear complex.^51^ Concentration-dependent adsorption experiments revealed that Co-HITP exhibited significantly lower *Q*_max_ values across all tested contaminants compared to its hydroxy-functionalized counterpart. Specifically, Co-HITP showed *Q*_max_ values of 58 mg g^–1^ for Cd^2+^, 279 mg g^–1^ for Hg^2+^, and 96 mg g^–1^ for Pb^2+^, comparable to those of Cu-HHTP, which features an eclipsed crystal structure and lacks intercalated layers (Figures S40–S43). To probe the role of surface charge, we performed dye adsorption experiments and zeta potential measurements, as detailed in Section S5 and Figures S44–S52. All HHTP-MOFs displayed (i) preferential adsorption of the cationic methylene blue dye over the anionic methyl orange dye, and (ii) consistently negative zeta potentials, confirming the presence of negatively charged surfaces in aqueous media. These findings highlighted the importance of both electrostatic attraction and structural features in enhancing ion uptake. While the intercalated architecture increases the density of accessible capping sites, which, in turn, facilitates electron transfer during redox-active adsorption and contributes to the superior performance of Co-HHTP over Co-HITP and Cu-HHTP, the higher negative surface charge and BET surface area of Co-HHTP relative to Ni-HHTP likely accounted for its higher adsorption capacity (Figures S15, S16, and S52).

### 4.3 Adsorption Kinetics

Having established the removal capabilities of the suite of M-HHTP MOFs toward the heavy metal cations, we examined their kinetic profiles at a fixed 250 ppm concentration of contaminants. All the MOF-contaminant systems exhibited a rapid initial increase in adsorption capacity (*Q*_t_) within the first few minutes, followed by a gradual plateauing as the concentration gradient decreased and binding sites approached saturation (Figure 2b).^52^ During the first 10 min, all MOFs displayed comparable adsorption rates, likely reflecting dominant external surface interactions at early time points. Among the tested materials, Co-HHTP consistently achieved the highest *Q*_t_ values, reaching 111 ± 6 mg g^–1^ for Cd^2+^, 396 ± 17 mg g^–1^ for Hg^2+^, and 375 ± 10 mg g^–1^ for Pb^2+^ within a 3 h contact time. In contrast, Cu-HHTP exhibited the lowest uptake under indistinguishable conditions, achieving *Q*_t_ values of 40 ± 6, 132 ± 12, and 139 ± 4 mg g^–1^ for Cd^2+^, Hg^2+^, and Pb^2+^, respectively. This kinetic trend mirrored the findings from the concentration-dependent studies and is likely attributed to the unique stacking arrangement of Co- and Ni-HHTP compared to Cu-HHTP.

To elucidate the dominant adsorption mechanism, we fitted the experimental kinetic data to the linearized forms of the pseudo-first-order, pseudo-second-order, and Temkin models, as detailed in Section S6.1. For all MOF-pollutant combinations, the pseudo-second-order model provided the best fit to the experimental data, as indicated by high correlation coefficients obtained from least-squares regression (Figures S53–S64). This result suggested that chemisorption is the dominant interaction mechanism, with adsorption rates governed by the binding of metal ions to active sites on the MOF surfaces.^53^ Further support for chemisorption came from the D-R model analysis, which yielded mean free energy (*E*) values exceeding 8 kJ mol^–1^ across the different MOFs (Tables S1–S4), consistent with energy ranges typical for chemical adsorption.^22^ To gain deeper insight into the adsorption process for Co-HHTP, we employed the intraparticle diffusion model based on the Weber-Morris Model (eq S12). The resulting kinetic profile revealed a triphasic process involving (i) an initial rapid uptake phase dominated by external surface interactions, followed by (ii) a slower diffusion-controlled phase reflecting intraparticle transport and interaction with internal adsorption sites, and (iii) a final equilibrium phase as active sites became saturated (Figure S65).

### 4.4 Mechanistic Insights into MOF-Analyte Interactions

To complement our adsorption and kinetic studies, which revealed preferential removal of Hg^2+^ and Pb^2+^ over Cd^2+^, with the highest affinities observed in Co-HHTP, followed by Ni-HHTP and Cu-HHTP, we employed *ex situ* XPS to monitor changes in the oxidation states of HHTP ligands, metal nodes, and the heavy metal ions following exposure to different concentrations of Cd­(II), Hg­(II), and Pb­(II). High-resolution XPS spectra of the O 1s region revealed redox-mediated interactions between the MOFs and Hg^2+^ ions (Figure 3a). At 500 ppm of Hg­(II), both Co-HHTP and Ni-HHTP acted as reductants, transferring electrons from the HHTP moieties to Hg^2+^, leading to its partial reduction and the concurrent oxidation of the ligand. This redox process emerged clearly in the decreased C–O to C=O ratio, which shifted from 1:1 in the pristine MOFs to 2:5 for Co-HHTP and 7:10 for Ni-HHTP after adsorption of Hg­(II) (Figure S66a,b). Correspondingly, high-resolution Hg 4f spectra and PXRD confirmed the formation of crystalline Hg_2_Cl_2_ in both Co-HHTP and Ni-HHTP, indicating reduction of Hg­(II) to Hg­(I) (Figures 3b and S67). Notably, the extent of HHTP oxidation correlated with increasing Hg^2+^ concentration, as indicated by the progressive growth of the C=O signal upon increasing [Hg^2+^] from 20 to 500 ppm (Figure S66). In contrast, Cu-HHTP exhibited negligible changes in the O 1s spectra and only minor formation of Hg­(I) species (Figures 3b and S66, S67), suggesting limited redox interaction with Hg­(II). Across all MOFs, the oxidation states of the metal nodes remained unchanged following Hg^2+^ exposure (Figure S68), indicating that redox processes occurred primarily at the ligand level rather than the metal centers. Exposure of MOFs to Pb­(II) led to similar trends, albeit with less pronounced HHTP oxidation. In Co-HHTP and Ni-HHTP, the HHTP ligand underwent partial oxidation, while Pb­(II) experienced a partial reduction to Pb^0^. In contrast, Cu-HHTP showed no detectable redox activity upon Pb^2+^ exposure. (Figures 3c,d and S69–S71).

**Figure 3:**
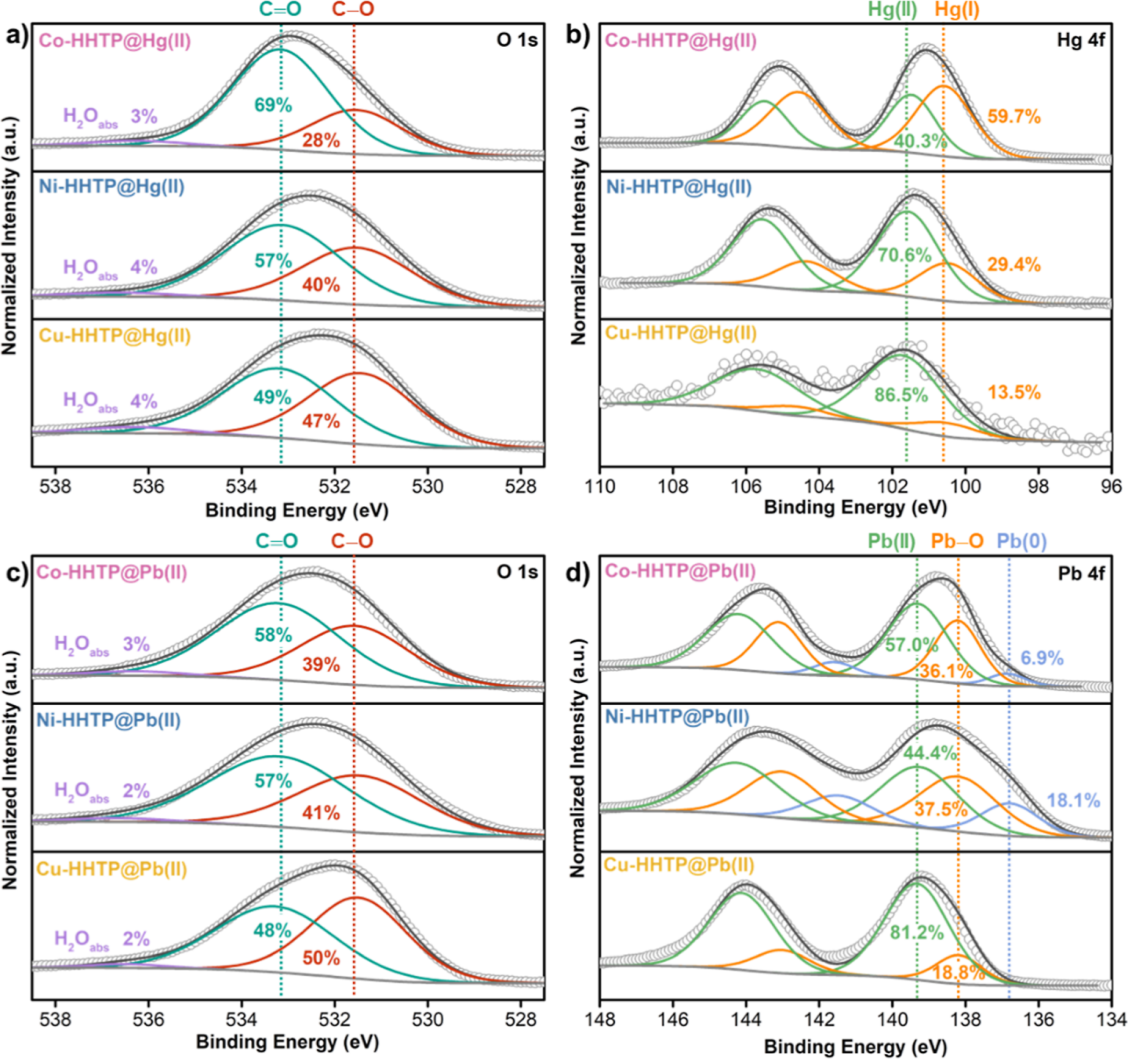
High-resolution XPS spectra of (a) oxygen (O 1s) and (b) mercury (Hg 4f) elements of the MOFs following exposure to 500 ppm of HgCl_2_. High-resolution XPS spectra of (c) oxygen (O 1s) and (d) lead (Pb 4f) elements of the MOFs following exposure to 500 ppm of Pb­(NO_3_)_2_ under ambient conditions.

Exposure to Cd^2+^ ions, on the other hand, induced no observable redox activity in any of the MOFs. XPS confirmed that Cd­(II) remained in the +2 oxidation state postadsorption, with no changes in the oxidation states of the ligands or metal nodes (Figures S72–S75). We attributed the observed redox selectivity to the standard reduction potentials (*E*°) of the heavy metal ions, shown in eqs 2–4.

2 $${2Hg}^{2+}+2e^{-}\to {Hg}_{2}^{2+},E^{0}=+0.91\;V$$

3 $${Pb}^{2+}+2e^{-}\to Pb\left(0\right),E^{0}=-0.13\;V$$

4 $${Cd}^{2+}+2e^{-}\to Cd\left(0\right),E^{0}=-0.403\;V$$

Hg^2+^, and to a lesser extent, Pb^2+^, serve as potent oxidizing agents, readily accepting electrons from the redox-active HHTP ligands. We hypothesized this redox interaction drove the ligand from its tris-semiquinone state [sq–sq–sq]^3–^ to a more oxidized configuration.^54,55^ In contrast, the reduction of Cd^2+^ is likely thermodynamically unfavorable under the experimental conditions, which explains both, the lack of redox interaction, and the lower removal efficiency of Cd^2+^ compared to the other contaminants. Taken together, these findings suggested that the selective redox activity in HHTP-MOFs likely arises from the accessibility of HHTP active sites and the reduction potentials of the heavy metal ions, with Hg^2+^ and, to a lesser extent, Pb^2+^ undergoing ligand-mediated reduction. At the same time, Cd^2+^ remains redox-inactive, explaining the observed low capture trend.

Beyond redox–driven interactions, additional spectroscopic and microscopic analyses on Co-HHTP revealed evidence of both physisorption and chemisorption. ATR-IR spectra, shown in Figure S76a, remained essentially unchanged after ion adsorption, suggesting the retention of the overall coordination network of the MOF. However, we noted a red shift in the C–O stretching band at 1173 cm^–1^ to lower wavenumbers of 1171, 1162, and 1153 cm^–1^ after Cd­(II), Pb­(II), and Hg­(II) exposure, respectively. Concurrently, Raman spectroscopy revealed a red shift in the G-band from 1577 cm^–1^ in the pristine Co-HHTP to 1573, 1572, and 1563 cm^–1^ for Co-HHTP@Cd­(II), Pb­(II), and Hg­(II), respectively (Figure S76b). These spectral shifts suggested noncovalent interactions between the heavy metals and Co-HHTP, which we attributed to physisorption via cation-dipole and cation–π interactions involving the polar C–O bonds and the electron-rich aromatic cores of the HHTP ligands.^56–58^ Further analyses confirmed that adsorption occurred both at the surface and within the porous network of Co-HHTP. SEM–EDX mapping images verified the uniform distribution of metal contaminants across the MOF crystal surfaces (Figures S77–S79). In parallel, BET surface area measurements demonstrated substantial pore filling, with the surface area of Co-HHTP decreasing from 341 m^2^ g^–1^ in the pristine state to 16, 14, and 17 m^2^ g^–1^ following adsorption of Cd­(II), Pb­(II), and Hg­(II), respectively (Figure S80).

Overall, the combination of these interactions limited the regeneration and recyclability performance of Co-HHTP, evidenced by three main factors. First, the morphology of the MOF changed significantly following adsorption of 100 ppm cations, as observed in the SEM micrographs in Figure S81, from nanorod-like crystals to block-like structures in the case of Pb^2+^ and Cd^2+^, and nanowires for Hg^2+^, indicative of the formation of Hg_2_Cl_2_.^59^ Second, attempts to desorb heavy metals via acid treatment were largely ineffective, preventing the reuse of the MOF in subsequent adsorption–desorption cycles (Figure S82). Third, the framework underwent moderate degradation during adsorption and complete degradation following acid treatment (Figure S83). Collectively, these observations indicate that, under the studied conditions, Co-HHTP exhibits limited recyclability and reusability, emphasizing the need for further optimization to improve its practical applicability.

### 4.5 Molecular Modeling

To provide atomic-level details of the mechanisms occurring during the diffusion of heavy metal ions within the MOF channels, we employed computational chemistry calculations at the DFT level of theory, as already done in ref 32. We have employed an approach made of a combination of PW (Plane-Wave) periodic models for the estimation of the interaction energies between the heavy metal ions and the MOF channels, and of finite models with localized basis sets for the derivation of the Electrostatic Potential Iso-surfaces; a deeper description of the computational approach is provided in the Supporting Information. We focused on the combination of Co-HHTP and Hg^2+^ metal cation. A few portions of the unit cells are displayed in panels (C–D) of Figure 4, where Hg^2+^ are close to the MOF walls and surrounded by Cl^–^ counterions and water molecules.^60,61^ As suggested by the literature, the MOFs with the best performance are those containing nucleophilic functional groups carrying sulfur, nitrogen, or oxygen atoms,^62^ where the metal cations can establish a Coulombic interaction with negative heteroatoms or regions with diffuse (aromatic) negative charge, as shown, for example, in the investigation of the adsorption of Hg^2+^ on a Zr-based MOF or of Pb^2+^ on a UiO-66 MOF loaded with single and double amino and thiol-functionalities to enhance sorption properties.^63,64^ The Co-HHTP MOF, with its nucleophilic walls and negative zeta potential, is an ideal candidate. Its negative charge character is confirmed by the electrostatic density map of Figure 4A, which reveals that the most negative potential regions are around the catechol oxygens, both from the extended 2D sheets, and the 0D cobalt–catechol complexes. Instead, the aromatic areas above and below the catechol linkers display moderate negative potentials. However, due to the stacking of the MOF layers, the intercalation of adsorbate species is hindered in such configurations.

**Figure 4:**
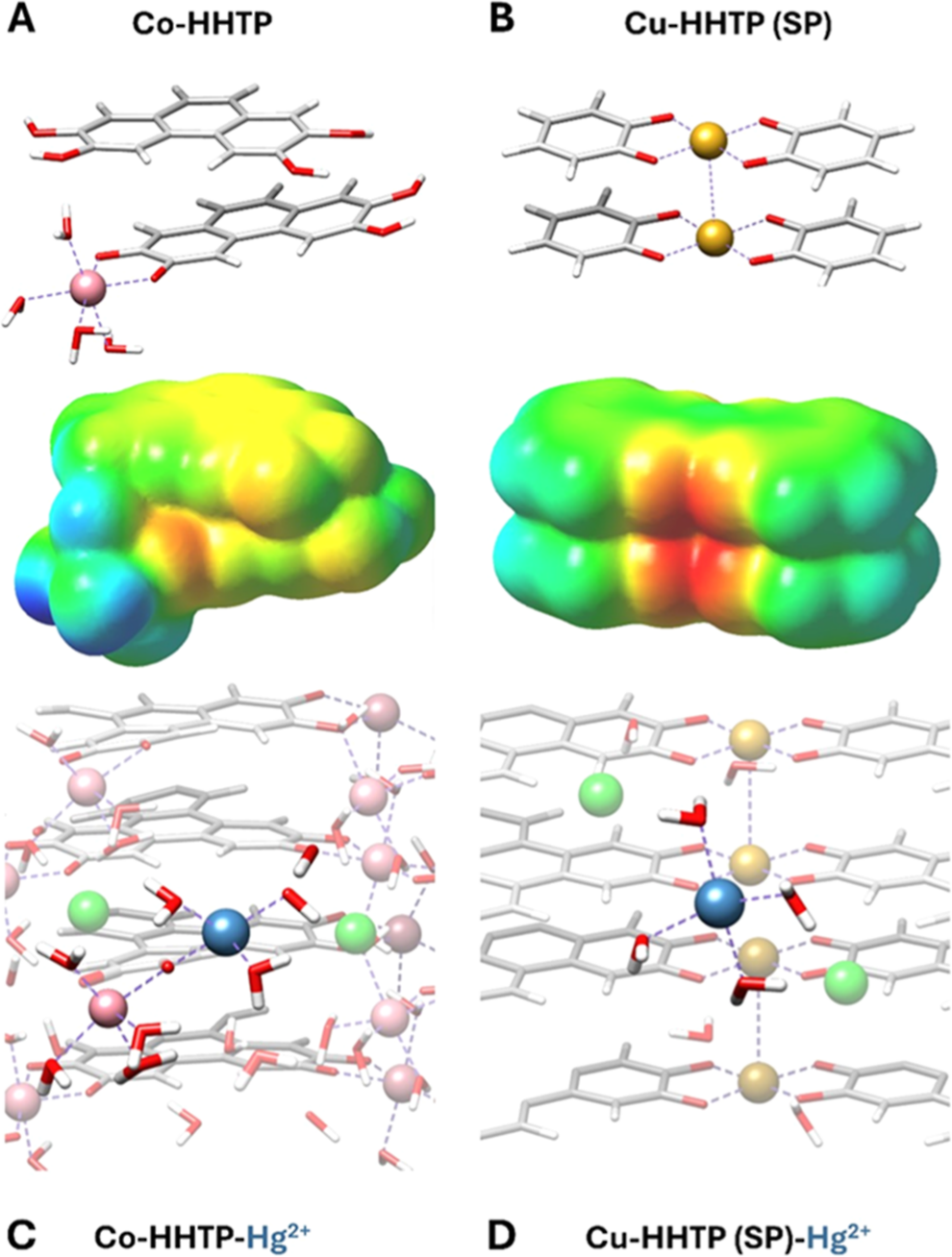
Structure of Co-HHTP (A) and Cu-HHTP (B) MOFs. For the Cu-HHTP system, only the slipped-parallel (SP) arrangement has been considered. Top and/or side views of the structure of hydrated Hg cations interacting with the walls of the Co-HHTP (C) and Cu-HHTP (D) MOFs. Color code: C gray, O red, H white, Cu light yellow, Co pink, Hg blue.

The lowest-energy configuration, shown in Figure 4C, corresponds to the adsorption of Hg^2+^ on a catechol oxygen belonging to the 0D complexes of Co-HHTP, as every attempt to induce the adsorption next to catechol oxygens belonging to the 2D sheets was unsuccessful. The heavy metal ion completed its coordination shell with water molecules. We have observed that the interaction of Hg^2+^ with the MOF walls induces the dissociation of a water molecule nearby, resulting in the adsorption of OH^–^ on the metal cation and H^+^ on a neighboring catechol oxygen of the 2D sheets (Figure 4C). This process precedes the redox activity that accompanies the adsorption of Hg^2+^ on the Co-HHTP system. Indeed, water dissociation indirectly carries a negative charge via OH^–^ to Hg^2+^ ions (hence inducing its reduction), and the hole held by the proton, transferred to the 2D sheets, can cause the oxidation of the neighboring HHTP ligands.

The comparison with our previous investigation,^65^ where the proton transfer to the 2D sheets of the Cu_3_(HHTP)_2_ system during the oxidation of SO_2_ induced the reduction of Cu­(II) to Cu­(I), revealed that in this case the cobalt nodes of the MOF did not change their chemical environment and kept their native charge, in agreement with the experimental observation (e.g., the MOF metal nodes are not involved in the redox process). To distinguish the reactive and unreactive events, we analyzed another configuration (C1, shown in Figure S85), characterized by the adsorption of undissociated water molecules on Hg^2+^. Analyzing the Cu-HHTP system (configuration D in Figures 4 and S86), we found a similar scenario, because the nucleophilic walls terminated by catechol oxygens also favored the adsorption of heavy metal cations, as highlighted by the electrostatic potential map of Figure 4B. Indeed, the cation was adsorbed in the neighborhood of the catechol oxygens belonging to extended 2D sheets, completing its coordination shell with water molecules. In this case, Hg^2+^ did not induce any dissociation of the surrounding water molecules, in agreement with the lack of redox activity (experimental observation).

To provide a semiquantitative description of the interaction between the metal cations and the MOF walls, we have reported some structural parameters of the equilibrium geometries and some energy descriptors corresponding to “two-body” interactions between complementary portions of the whole system (Table 1). Comparing the competition between hydration and interaction with the MOF walls, we observed that Hg^2+^ favors interaction with Co-HHTP rather than with Cu-HHTP, in agreement with the larger ion uptake values for the former system. In fact, in the case of Co-HHTP, for both reactive and unreactive configurations, Hg^2+^ has a smaller hydration energy and a larger interaction with the MOF in dry conditions (same geometry of the hydrated case but without the explicit insertion of water molecules in the evaluation of the interaction). Examination of the interaction of the MOF walls with the hydrated metal ion (first line) reveals that the values for the Co-HHTP systems are much larger than those for Cu-HHTP. The geometrical descriptors support this analysis, as the metal ion was adsorbed farther in Cu-HHTP than in Co-HHTP (2.45 vs 2.13 Å).

**Table 1:** Energetic and Structural Analysis of Some configurations Shown in Figure 4A^a^

	Co-HHTP		Cu-HHTP
interaction type	C	C1	D
MOF–Hg^2+^ (2 Cl-)/WAT (eV)	11.22	8.66	2.38
Hg^2+^ (2 Cl-)–WAT (eV)	2.12	1.82	4.54
Hg^2+^(2 Cl-)–MOF (eV) dry conditions	7.19	7.19	1.91
D(M-O) (Å)	2.13	2.13	2.45
DAVE(M-OWAT) (Å)*	2.41(3)	2.41(3)	2.36 (4)

a The number of water molecules used to make the average is indicated in parentheses and corresponds to a M–O distance within 3.0 Å.

Furthermore, the number of water molecules in the first hydration shell is larger for Cu-HHTP, confirming the tendency to optimize the interaction with the solvent rather than with the MOF walls in this system. We hypothesize that the difference in hydration is due to the increased interaction of the heavy metal cations with the 0D complexes, where the catechol oxygens carry a more negative charge (as estimated via an NBO analysis) than in the case of the 2D sheets. Further confirmation comes from the tendency of the captured metal ion to “migrate” from the 2D catechol oxygens to the 0D ones, observed in the optimization of Hg^2+^ near the walls of the Co-HHTP system. In the case of Cu-HHTP, we also performed some local optimizations by using the alternative (ABC) stacking with OMS (Open Metal Sites). However, the resulting lowest energy configurations still corresponded to the adsorption of the metal ion in the neighborhood of the catechol oxygens, with a very similar energy landscape to that observed for the slipped-parallel configuration. Finally, we note that the difference between the reactive and unreactive configurations in Co-HHTP MOF stems from the increased values of both the hydration energy and the interaction of the hydrated cation with the MOF walls. The first comes from the negative charge carried by the OH^–^ group after dissociation. In contrast, the second comes from (i) the choice of a (higher) different energy reference (comprising a dissociated water molecule) and (ii) the added interaction between H^+^ and a second catechol oxygen from the 2D sheets.

### 4.6 Matrix-dependent Adsorption Efficacy of Co-HHTP

To assess the applicability of these layered materials in potential water treatment scenarios, we evaluated the adsorption performance of Co-HHTP in various aqueous environments. First, we examined the removal efficiency of the MOF adsorbent in tap water, river water (from Connecticut), and ocean water (from the Atlantic), each spiked with 100 ppm of Cd^2+^, Hg^2+^, and Pb^2+^ cations. As shown in Figure 5a, Co-HHTP maintained robust adsorption uptake for all cations in both tap and river waters, suggesting that common surface water interferents exert minimal impact on the MOF performance. Nonetheless, the MOF exhibited a remarkable decrease in *Q*_e_ in spiked ocean water, which we attributed to its high salinity exceeding 35,000 ppm.^66^ PXRD analysis of Co-HHTP after soaking in these matrices revealed the formation of new salt phases in ocean water, while the MOF preserved partial crystallinity in tap and river water (Figure S84). Next, we assessed the ability of Co-HHTP to capture heavy metals at parts per billion (ppb) levels. As shown in Figure 5b, the MOF adsorbent achieved removal efficiencies of up to 97%, 79%, and 100% of 200 ppb of Cd^2+^, Hg^2+^, and Pb^2+^, respectively. This performance reduced Pb­(II) and Cd­(II) concentrations well below the safe drinking water thresholds,^67^ while lowering Hg­(II) levels to 42 ppb. Furthermore, Co-HHTP maintained outstanding removal efficiencies in the presence of 200 ppm of cointerfering ions, suggesting that the presence of oxoions (nitrate, sulfate, acetate, phosphate, and carbonate) and halides (Cl^–^) of distinct ionic radii, basicity, and charge densities have minimal effect on the adsorption capacity of the MOF (Figure 5c). Overall, these findings highlight the high sensitivity, selectivity, and matrix tolerance of Co-HHTP for the removal of divalent heavy metals in diverse aqueous environments, supporting its potential for deployment for practical water purification.

**Figure 5:**
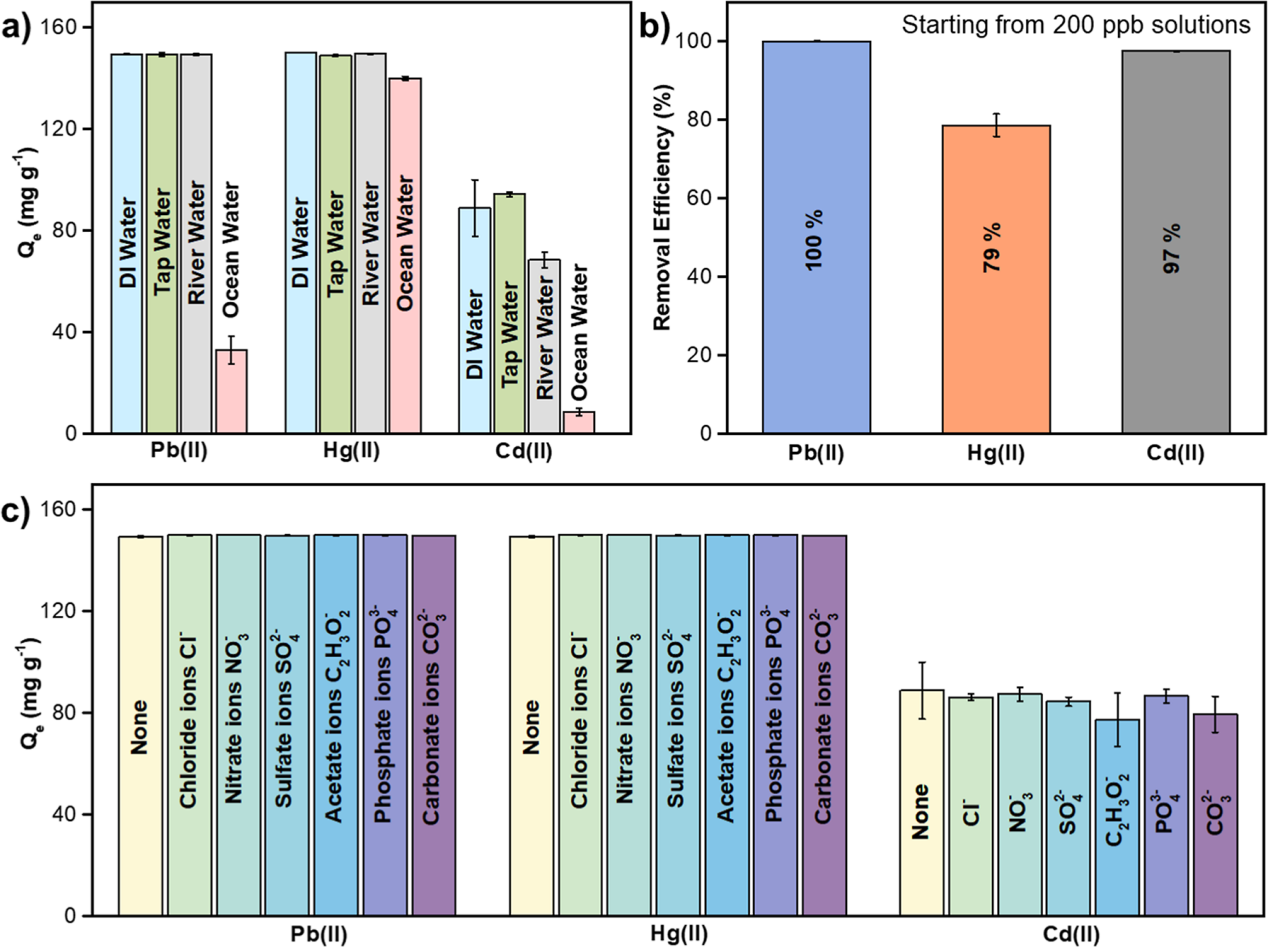
(a) Uptake capacity of Co-HHTP in different water sources spiked with 100 ppm of Pb^2+^, Hg^2+^, and Cd^2+^ ions. (b) Removal efficiencies of Co-HHTP toward 200 ppb of heavy metal pollutants and (c) effect of coexisting ionic species (200 ppm each) on the removal of 100 ppm of Pb­(II), Hg­(II), and Cd­(II) from DI water. Conditions: m_MOF_ = 2 mg, V_solution_ = 3 mL, and *T* = 298 K. Note that the error bars represent the standard deviation from the mean of three independent experiments.

### 4.7 Deposition of Co-HHTP on Textile Fabrics

MOF powders often present limitations for practical deployment due to their fine particle size and tendency to aggregate in solution. To improve their handling and recovery process, we deposited Co-HHTP onto textile substrates via a one-pot solvothermal method (Figure 6a and Section S9). Optimization studies revealed that reacting 50 mM cobalt acetate with 70 mM HHTP in a 4:1 water to 1,3-dimethyl-2-imidazolidinone (DMI) solvent mixture at 75 °C overnight, in the presence of 1.5 × 1.5 cm textile swatches, yielded the most uniform and crystalline MOF coatings (Figures S87–S90 and Table S8). PXRD patterns of the resulting MOF@textile composites, shown in Figures 6b and S91, confirmed the successful integration of Co-HHTP on cotton, polyester, and silk fabrics, with diffraction patterns matching those of bulk Co-HHTP. SEM micrographs and elemental mapping, displayed in Figures 6a and S92–S95, revealed uniform coatings of rod-like Co-HHTP crystals across the textile fibers. Additional solvothermal deposition cycles increased total MOF mass loading, reaching up to 33 ± 8 mg of MOF per cm^2^ of textile after four cycles. However, repeated deposition significantly diminished adhesion, likely due to poor interfacial bonding between MOF layers. Therefore, we selected the single-deposition protocol for subsequent adsorption experiments.

**Figure 6:**
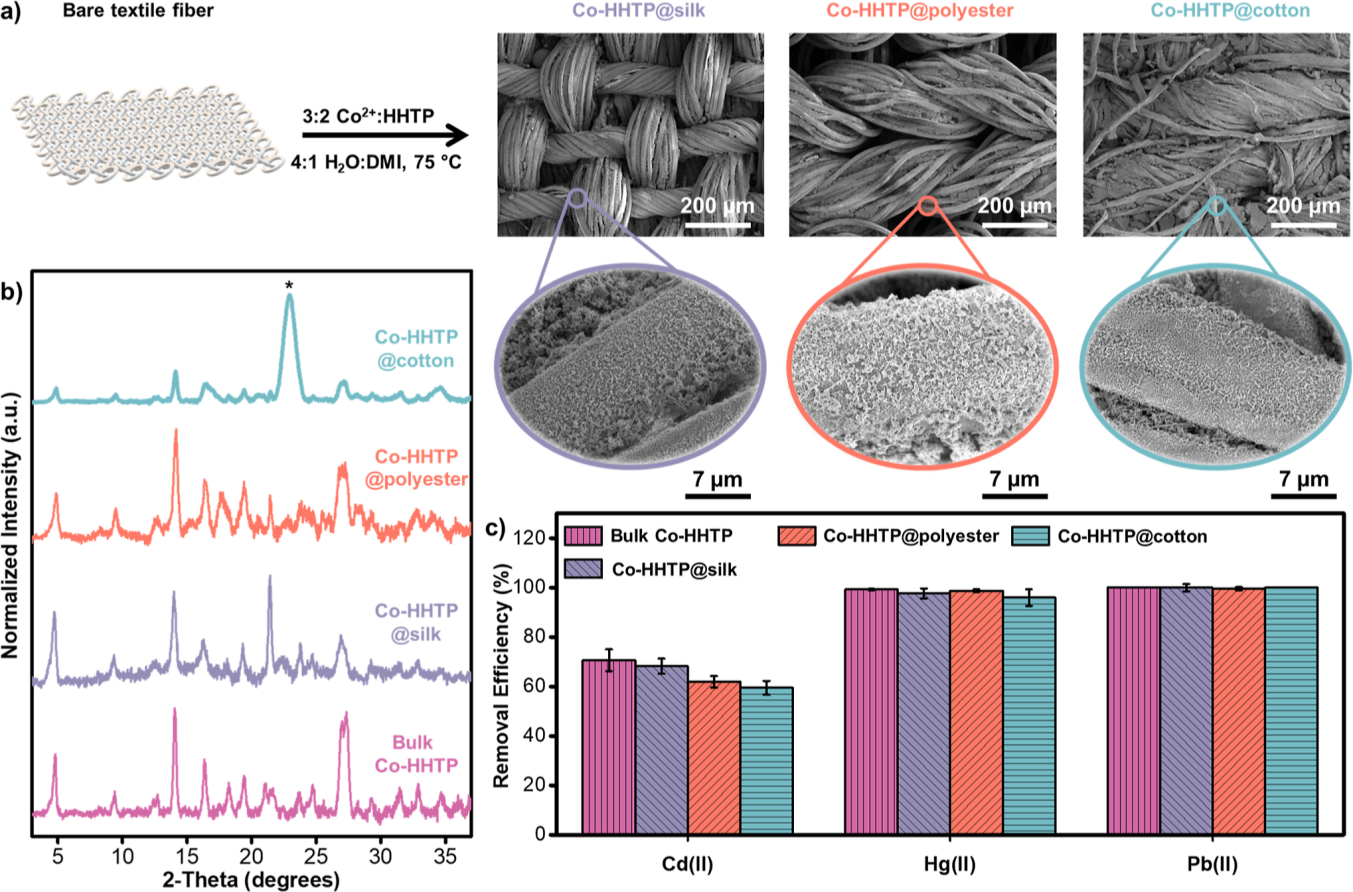
Fabrication of Co-HHTP on textile swatches using a solvothermal method for the removal of heavy metal pollutants from water. (a) Synthetic procedure and SEM micrographs of Co-HHTP prepared on silk, polyester, and cotton swatches. (b) PXRD patterns of Co-HHTP on textile swatches. The asterisk marks the diffraction peak of the cotton fabric. (c) Removal efficiency (%) of Co-HHTP on textiles toward 50 ppm of Cd­(II), Hg­(II), and Pb­(II) in DI water.

Batch adsorption experiments using Co-HHTP@textile composites, conducted under conditions analogous to those used for bulk Co-HHTP, demonstrated comparable removal efficiencies at 50 ppm concentrations of heavy metal ions (Figure 6c). These results suggested that the adsorptive and redox-active sites of Co-HHTP remain accessible following textile integration, supporting the potential of these composites for water decontamination applications. Notably, Co-HHTP@textiles offered two distinct advantages over bulk MOF powders. First, the MOF crystals retained some crystallinity after heavy metal adsorption, in contrast to bulk Co-HHTP, which exhibited significant structural degradation (Figure S96). Second, the composites exhibited a 3-fold lower leaching of cobalt compared to their powdered counterpart following exposure to 50 ppm heavy metal solutions. Specifically, Co leaching from MOF@textile composites ranged between 0.95 and 1.7%, whereas bulk Co-HHTP released up to 4.9% cobalt in solution, as quantified by ICP-OES analysis of the postadsorption filtrate (Figure S97). We attributed the reduced leaching to the physical immobilization of MOF crystals on the textile surface, which mitigates particle dispersion and structural collapse during aqueous exposure.^68^

### 4.8 Detection of Heavy Metals in Water

Encouraged by the intrinsic conductivity, high adsorption capacity, and redox-activity of Co-HHTP, we investigated its potential for electrically transduced detection of Cd^2+^, Hg^2+^, and Pb^2+^ in water using amperometry. We fabricated Co-HHTP@textile swatches (0.5 cm × 4 cm) following our previously established procedure^32^ (Figure S98). After immersing the swatch in DI water, we connected its ends to a potentiostat via alligator clips and applied a 1 V bias voltage to equilibrate the system (Figure S99). Subsequent addition of 10 μL aliquots of heavy metal solutions at increasing concentrations (1–50 ppm) resulted in immediate increases in output current (μA), which we hypothesized are arising primarily from changes in the ionic strength of the solution. (Figure 7a). The current increases followed a linear, concentration-dependent trend (Figures 7b and S100–S103), consistent with the signal profile reported for other chemiresistive sensing platforms.^69^ Estimating the theoretical limit of detection (LoD) from these experiments using the protocol described in Section S10.4 yielded values in the low-ppm range (5.7–6.3 ppm), underscoring the effective signal transduction capability of Co-HHTP, while also emphasizing that detection is driven by ionic strength rather than high selectivity (Figure S104). Compared to previously reported MOF-based materials for combined adsorption and detection, Co-HHTP offers one of the highest adsorption capacities with moderate LoDs suitable for practical monitoring at the low-ppm level (Table S13). In tests with common interfering ions (30 ppm potassium and sodium salts containing chloride, nitrate, carbonate, phosphate, acetate, formate, and sulfate), Co-HHTP@textile retained moderate detection function over three successive heavy-metal additions with modest signal loss (Figure S105), supporting its robustness in moderately saline aqueous environments.

**Figure 7:**
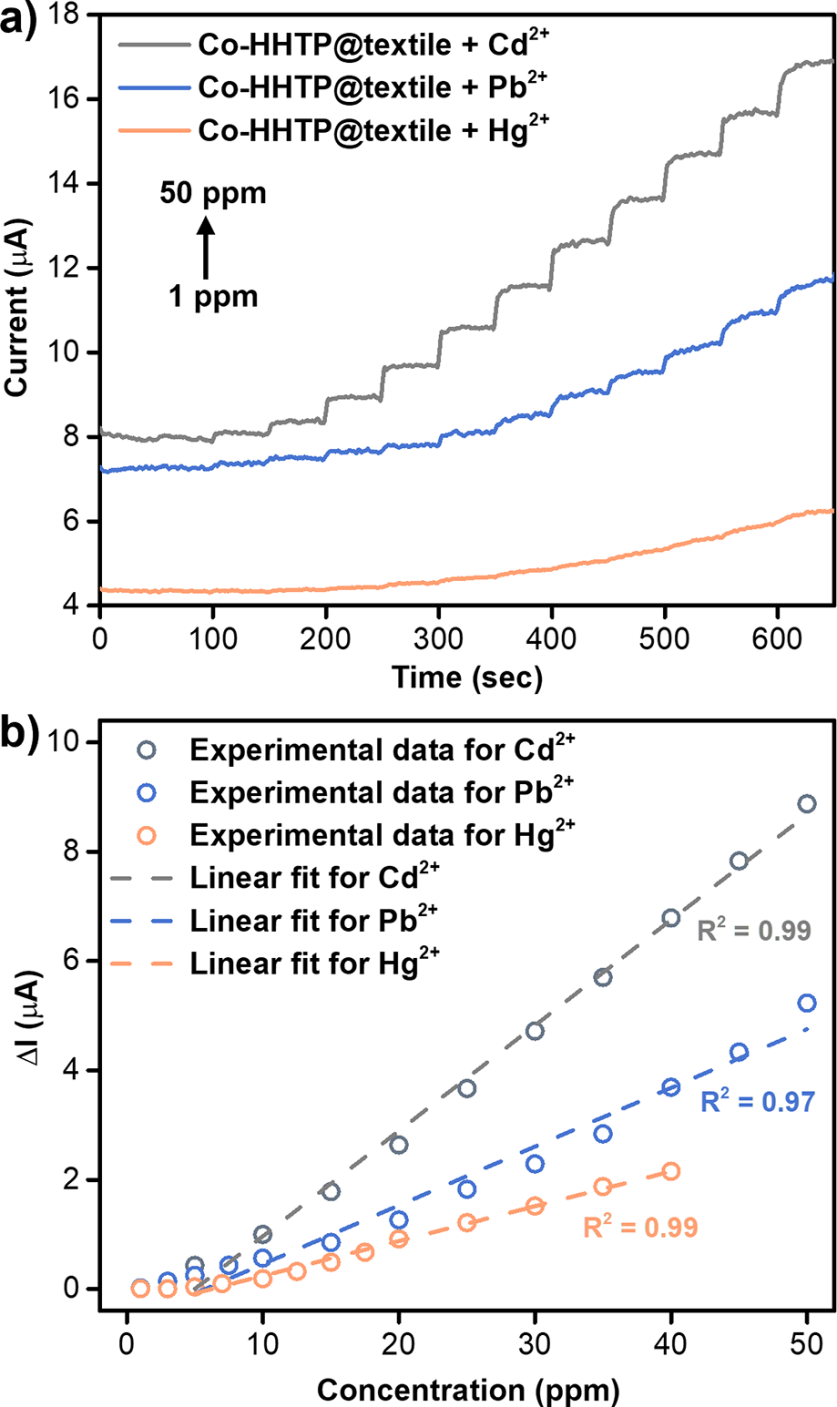
Detection of heavy metal cations in water using Co-HHTP@textile. (a) Amperometric sensing traces and (b) response (change in current) vs concentration curves of Co-HHTP@textile at 1.0 V upon successive additions of 10 μL aliquots of heavy metals (1–50 ppm) in DI water. No buffer was used in the detection experiments.

## 5 Conclusion

In summary, we reported the first systematic investigation of a series of redox-active M-HHTP cMOFs for the simultaneous capture, *in situ* reduction, and detection of Cd^2+^, Hg^2+^, and Pb^2+^ cations from water. Through a comparative analysis of Co-, Ni-, and Cu-HHTP frameworks, we establish clear structure–function relationships that link metal node identity, stacking arrangement, and ligand redox accessibility to heavy metal adsorption performance. Co- and Ni-HHTP, which feature intercalated layers of metal–catechol coordination complexes capped with water molecules, demonstrated pronounced redox activity due to their 9:4 metal-to-HHTP stoichiometry, promoting electron transfer from the HHTP ligand and enabling the partial reduction of Hg­(II) to Hg­(I) and Pb­(II) to Pb(0). In contrast, Cu-HHTP, with a more tightly packed, eclipsed stacking arrangement and a 3:2 Cu:HHTP stoichiometry, showed no detectable redox activity and the lowest uptake capacity. Spectroscopic investigations revealed a multimechanistic, synergistic removal pathway for Co- and Ni-HHTP, involving chemisorption, physisorption, and redox interactions that contributed to their superior removal efficiency. In addition to its high removal efficiencies, Co-HHTP displayed rapid adsorption kinetics, removing up to 80% of contaminants (250 ppm) within 120 min of contact time. Notably, its performance remained largely consistent across (i) different water matrices, (ii) the presence of 100 ppm coexisting ions, and (iii) at low ppb concentrations, lowering Cd^2+^ and Pb^2+^ levels below drinking water safety limits.

The deposition of Co-HHTP onto cotton, silk, and polyester fabrics yielded flexible MOF@textile composites that preserved bulk performance while enabling real-time detection at low ppm concentrations. Taken together, this work introduces a new class of intrinsically redox-active, multifunctional cMOFs for water remediation. Unlike prior redox-active cMOFs that require postsynthetic modifications or offer limited detection capabilities,^27,70^ Co-HHTP integrates intrinsic permanent porosity, redox-responsive ligands, and conductivity into a single platform. To the best of our knowledge, this is the first demonstration of a class of MOFs that enable simultaneous filtration, detoxification, and amperometric detection of divalent heavy metal cations, offering a straightforward, cost-effective solution for developing next-generation materials suited for point-of-use water purification and rapid-response environmental remediation technologies.

## Supplementary Material



## Notes

Experimental methods, additional characterization, adsorption capacities, kinetic models, computational data, electrochemical sensing measurements (PDF)
