# Supplementary material for: Redox-Active Metal–Organic Framework Nanocrystals for the Simultaneous Adsorption, Detection, and Detoxification of Heavy Metal Cations
Source: ACS Appl Mater Interfaces. 2025 Dec 22;18(1):1218–30. doi: 10.1021/acsami.5c18562 (PMC12781065; doi:10.1021/acsami.5c18562)
Supplement: Supplementary file 1 [file am5c18562_si_001.pdf]

Supporting Information

Redox-Active Metal–Organic Framework Nanocrystals for the Simultaneous Adsorption, Detection, and Detoxification of Heavy Metal Cations

Patrick Damacet,[a]* Elissa O. Shehayeb,[a] Susanna Monti,[b] Giovanni Barcaro,[c]* and Katherine A. Mirica[a]*

[a]Department of Chemistry, Burke Laboratory, Dartmouth College, Hanover, New Hampshire 03755, United States

[b]CNR-ICCOM, Institute of Chemistry of Organometallic Compounds, Pisa I56124, Italy.

[c]CNR-IPCF, Institute for Chemical and Physical Processes, Pisa I-56124, Italy.

Correspondence to:

Patrick Damacet: Patrick.damacet.gr@dartmouth.edu

Giovanni Barcaro: Giovanni.barcaro@cnr.it

Katherine A. Mirica: Katherine.a.mirica@dartmouth.edu

Contents

1. Materials and methods 3

2. Characterization of M-HHTP (M = Co, Ni, and Cu) MOFs 4

2.1 Scanning electron microscope (SEM) 4

2.2 Powder X-ray Diffraction (PXRD) 6

2.3 Energy dispersive X-ray analysis (EDX) 7

2.4 Transmission electron microscope (TEM) 9

2.5 Attenuated total reflectance-infrared spectroscopy (ATR-IR) 12

2.6 Brunauer-Emmett-Teller (BET) analysis 12

2.7 Thermogravimetric analysis (TGA) 14

2.8 Electrical conductivity measurements 14

3. Concentration-dependent adsorption studies 16

3.1 Adsorption isotherm models 17

3.1.1 Langmuir adsorption model 17

3.1.2 Freundlich adsorption model 18

3.1.3 Temkin adsorption model 18

3.1.4 Dubinin-Radushkevich (D-R) adsorption model 19

3.2 Adsorption isotherms of lead ions (Pb^{2+}) 20

3.3 Adsorption isotherms of cadmium ions (Cd^{2+}) 24

3.4 Adsorption isotherms of mercury ions (Hg^{2+}) 28
3.5 Uptake capacities for Co-HHTP compared to reported MOF-based adsorbents 32
4. Synthesis, characterization, and adsorptive performance of Co-HITP 33
4.1 Bottom-up synthetic procedure of Co-HITP 33
4.2 Structural and morphological features of Co-HITP 34
4.3 Adsorption isotherms of Co-HITP 38
4.3.1 Adsorption isotherms of Pb^{2+} ions 38
4.3.2 Adsorption isotherms of Cd^{2+} ions 39
4.3.3 Adsorption isotherms of Hg^{2+} ions 40
4.4 Comparison in performance with Co-HHTP 41
5. Surface charge properties of MOFs 42
5.1 Dye uptake experiments 42
5.2 Zeta potential measurements 44
6. Time-dependent adsorption studies 48
6.1 Adsorption isotherm models 48
6.1.1 Pseudo-first order kinetic model 48
6.1.2 Pseudo-second order kinetic model 49
6.1.3 Elovich kinetic model 49
6.2 Kinetic isotherms of Pb^{2+} ions 51
6.3 Kinetic isotherms of Cd^{2+} ions 56
6.4 Kinetic isotherms of Hg^{2+} ions 61
6.5 Intra-particle diffusion model for Co-HHTP 65
7. Mechanistic insights into MOF–Metal Ion Interactions 67
7.1 MOF-Hg(II) interactions 67
7.2 MOF-Pb(II) interactions 69
7.3 MOF-Cd(II) interactions 71
7.4 Additional characterization for Co-HHTP 74
8. Molecular modeling 79
9. Fabrication of Co-HHTP on textile fabrics 82
9.1 Synthetic procedures for the preparation of Co-HHTP on textiles 82
9.2 Optimization efforts for depositing Co-HHTP on textiles 83
9.3 Characterization of Co-HHTP on textiles 88
9.4 Stability of Co-HHTP on textiles after adsorption 90

10. Chemiresistive detection of heavy metals with Co-HHTP@textile 91
10.1 Fabrication of Co-HHTP@textile swatches 91
10.2 Chemiresistive sensing setup and methods 92
10.3 Replicates of chemiresistive detection experiments 94
10.4 Estimation of the theoretical limit of detection (LoD) 97
10.5 Comparison of multifunctional performance with literature 98
10.6 Chemiresistive detection of heavy metals in the presence of interferences 99
11. References 99

1. Materials and methods

Glassware and magnetic bars used throughout this study were oven-dried at 100 °C prior to the start of every experiment. All reagents and solvents were purchased from commercial sources and used as received without purification: nickel(II) acetate tetrahydrate (>95 %), N,N-Dimethylformamide (>99.5 %, GC grade), 1,3-Dimethyl-2-imidazolidinone (>99.0%, GC), and sodium acetate (>98.5 %) were acquired from TCI. Copper(II) acetate anhydrous (98%), cobalt(II) acetate tetrahydrate (98%), cobalt(II) nitrate hexahydrate (99%), mercury(II) chloride (>98%), cadmium(II) chloride (99%), lead(II) nitrate (>99%), ammonium hydroxide (ACS reagent, 28-30%), and acetone (ACS reagent, ≥99.5 %) were purchased from Thermofisher scientific. 2,3,6,7,10,11-hexahydroxytriphenylene Hydrate (HHTP) and triphenylene-2,3,6,7,10,11-hexaamine hexahydrochloride (HATP·6HCl) were acquired from Ambeed.

Powder X-ray diffraction (PXRD) spectra were collected on a Rigaku MiniFlex Powder X-ray Diffractometer equipped with a Cu 600 W (40 kV, 15 mA, I = 1.54 Å) radiation source following background subtraction. The range between 3° and 40° 2θ was scanned with a step size of 0.02° and a scan rate of 4° per minute. Scanning electron microscopy (SEM) was performed on a Thermo Scientific Helios 5 CX DualBeam scanning electron microscope. X-ray photoelectron spectroscopy (XPS) experiments were carried out on Kratos Analytical AXIS Supra X-ray Photoelectron Spectrometer under ultrahigh vacuum (base pressure 10^{-7} Torr) equipped with a

monochromatic Al (Kα) X-ray source. Both survey and high-resolution spectra were obtained using a beam diameter of 200 μm. Thermogravimetric analysis (TGA) measurements were carried out on a TGA 55 instrument with 20°C/min ramp rate and air as the purge gas. Brunauer-Emmett-Teller (BET) nitrogen gas measurements were performed with 3Flex (Micromeritics, Norcross, Georgia) instrument at 77 K. Attenuated total reflectance infrared spectroscopy (ATR-IR) was performed on a Nicolet IS50 Spectrometer with a DLATGS detector. Raman spectroscopy measurements were carried out on a Horiba labRAM HR Evolution using a wavelength of 532 nm. Electrical resistance measurements were collected on a KAIWEETS ST600Y digital multimeter smart auto range voltmeter.

2. Characterization of M-HHTP (M = Co, Ni, and Cu) MOFs

2.1 Scanning electron microscope (SEM)

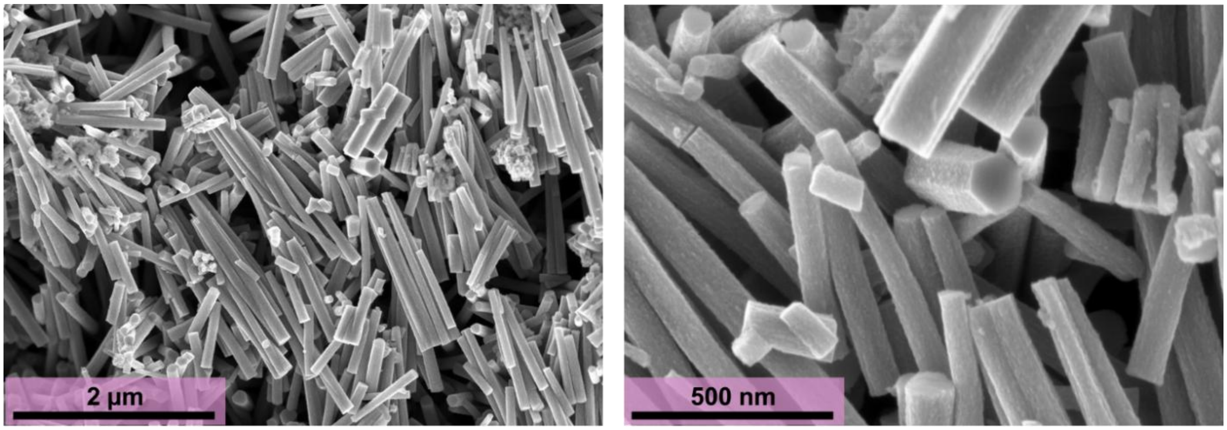

Figure S1. SEM micrographs of Co-HHTP at different magnifications.

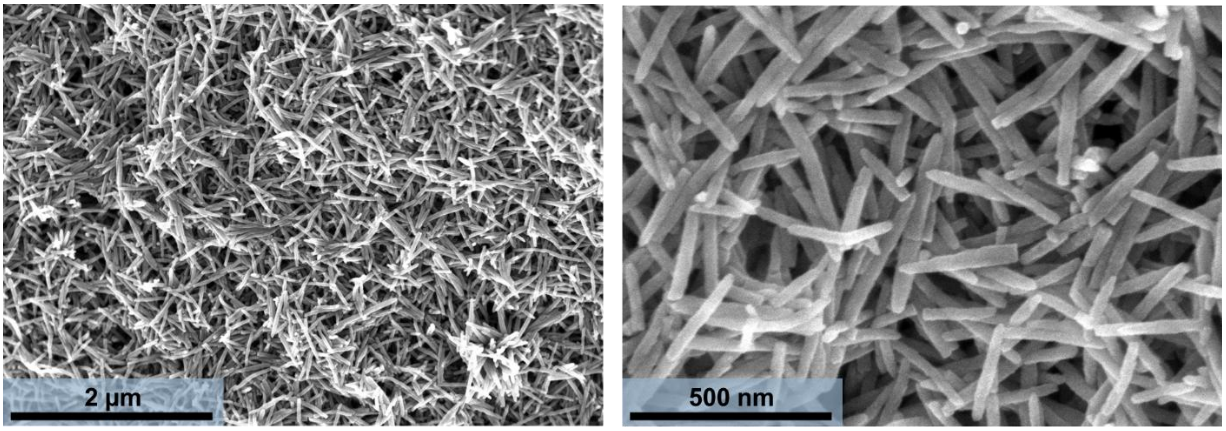

Figure S2. SEM micrographs of Ni-HHTP at different magnifications.

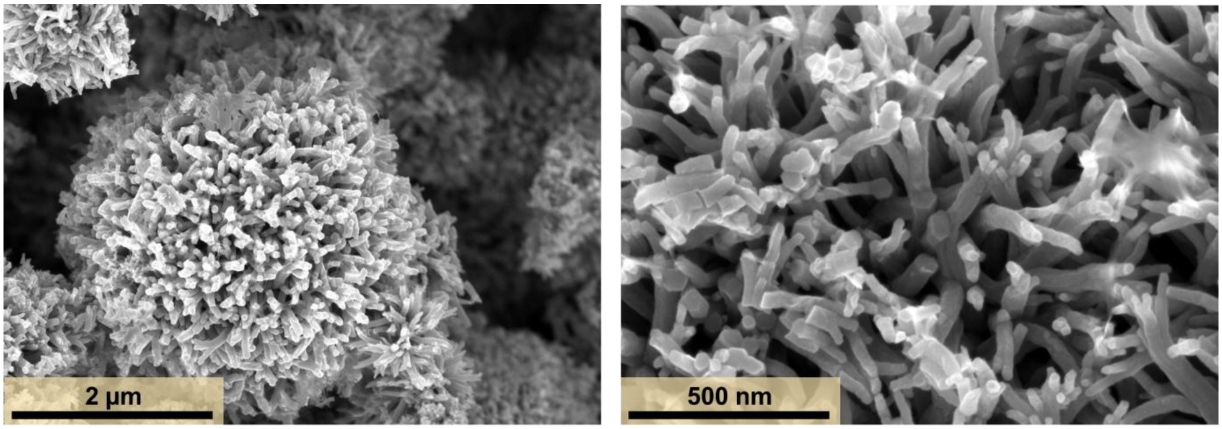

Figure S3. SEM micrographs of Cu-HHTP at different magnifications.

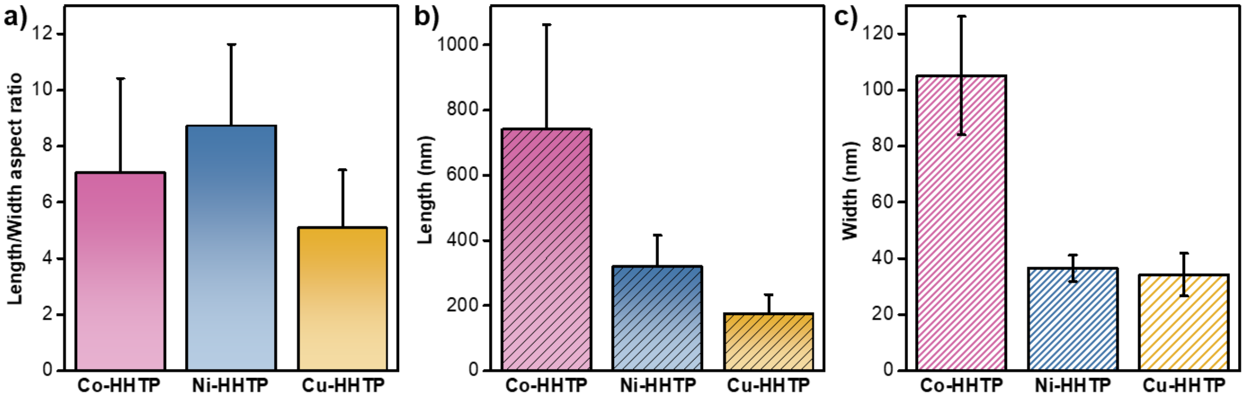

Figure S4. a) Length-to-width aspect ratio, b) length, and c) width of the MOF crystals determined from the SEM images using ImageJ software.

2.2 Powder X-ray Diffraction (PXRD)

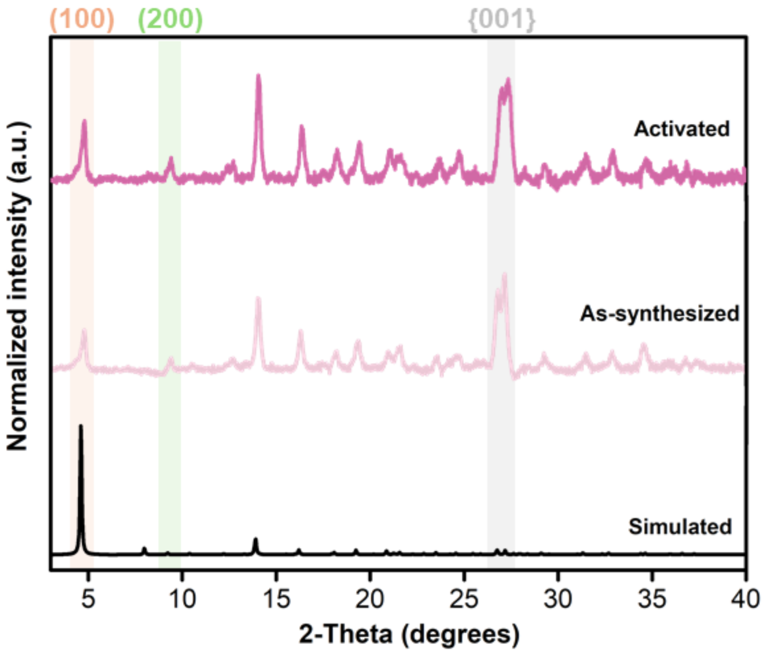

Figure S5. Powder X-ray diffraction patterns of Co-HHTP before and after activation.

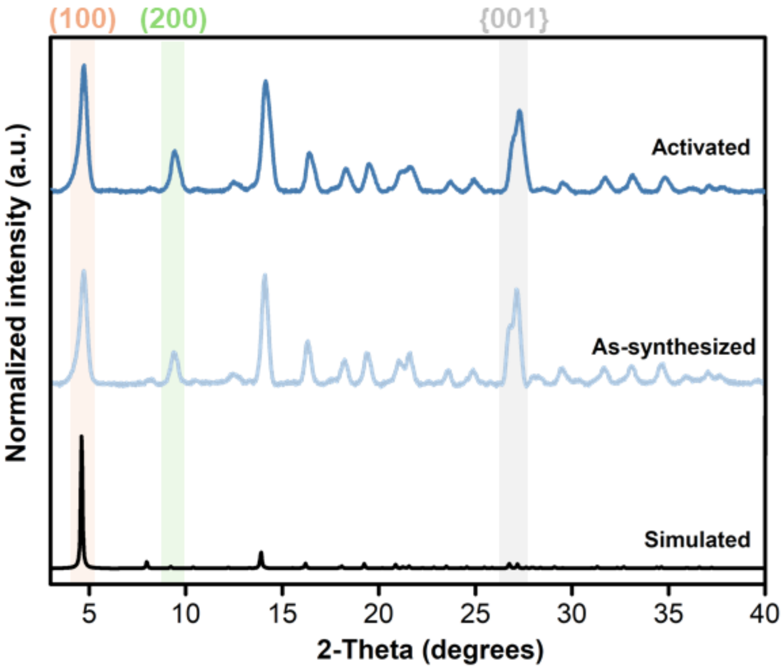

Figure S6. Powder X-ray diffraction patterns of Ni-HHTP before and after activation.

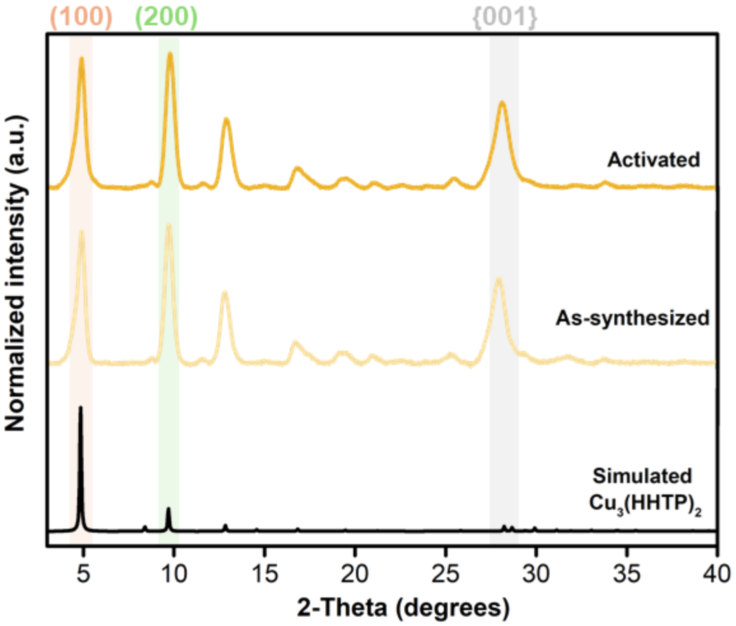

Figure S7. Powder X-ray diffraction patterns of Cu-HHTP before and after activation.

2.3 Energy dispersive X-ray analysis (EDX)

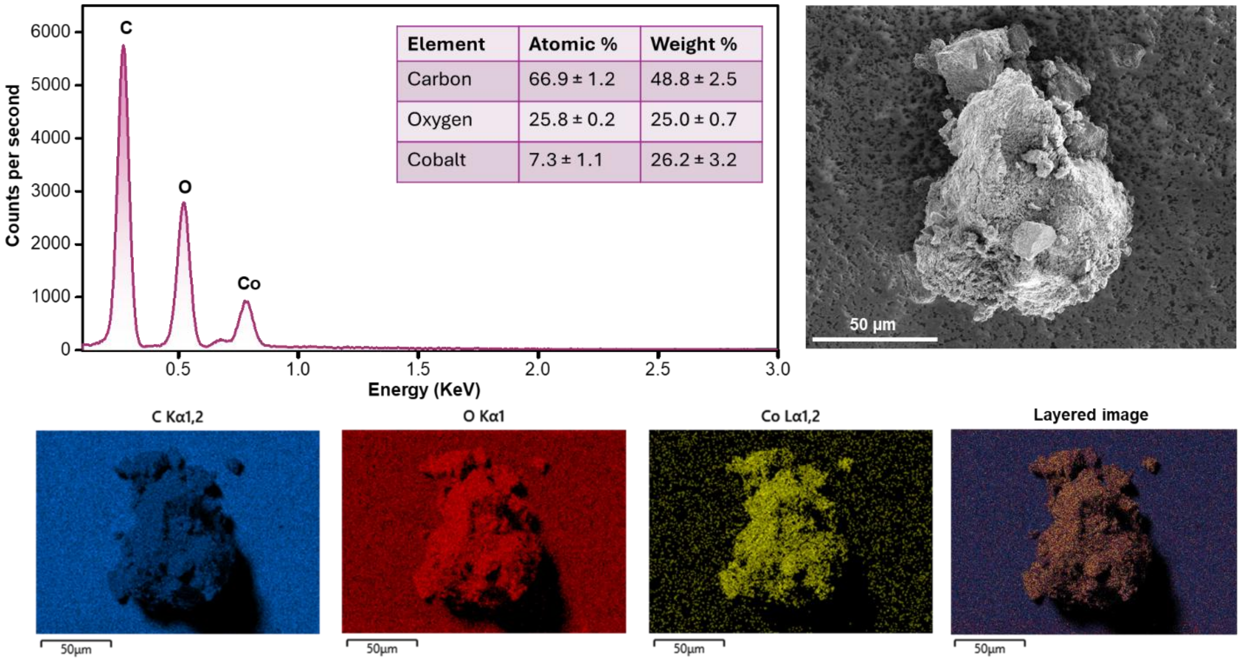

Element	Atomic %	Weight %
Carbon	66.9 ± 1.2	48.8 ± 2.5
Oxygen	25.8 ± 0.2	25.0 ± 0.7
Cobalt	7.3 ± 1.1	26.2 ± 3.2

Figure S8. EDX spectrum and elemental mapping images of Co-HHTP.

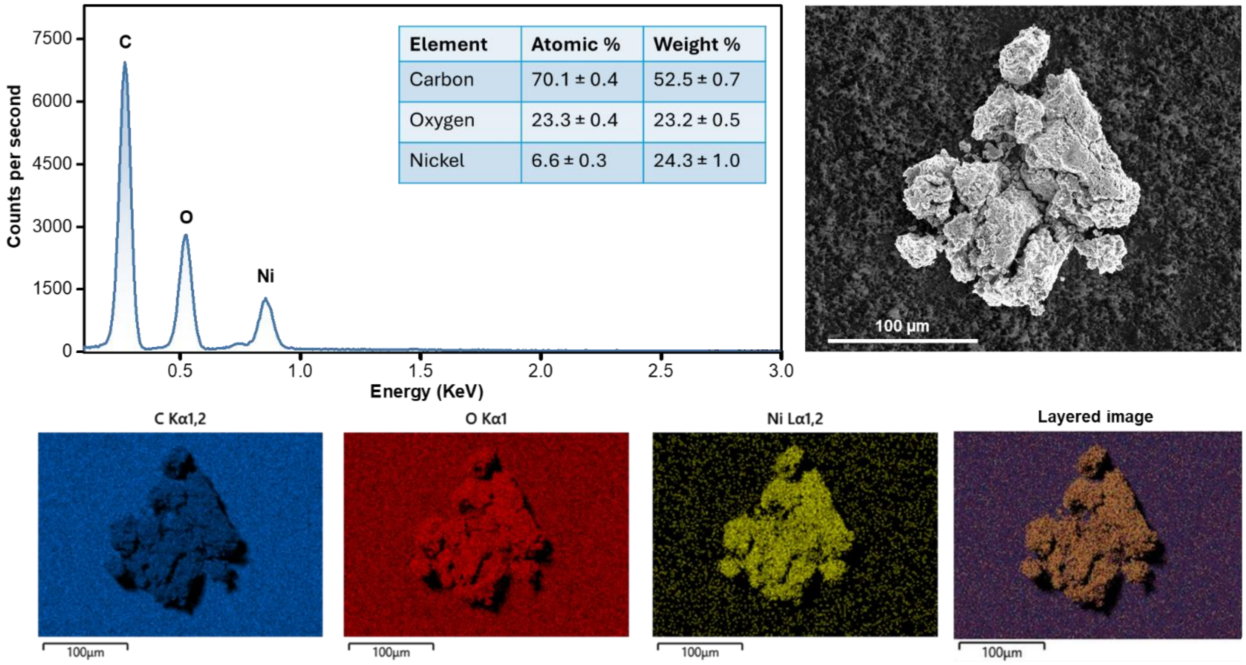

Element	Atomic %	Weight %
Carbon	70.1 ± 0.4	52.5 ± 0.7
Oxygen	23.3 ± 0.4	23.2 ± 0.5
Nickel	6.6 ± 0.3	24.3 ± 1.0

Figure S9. EDX spectrum and elemental mapping images of Ni-HHTP.

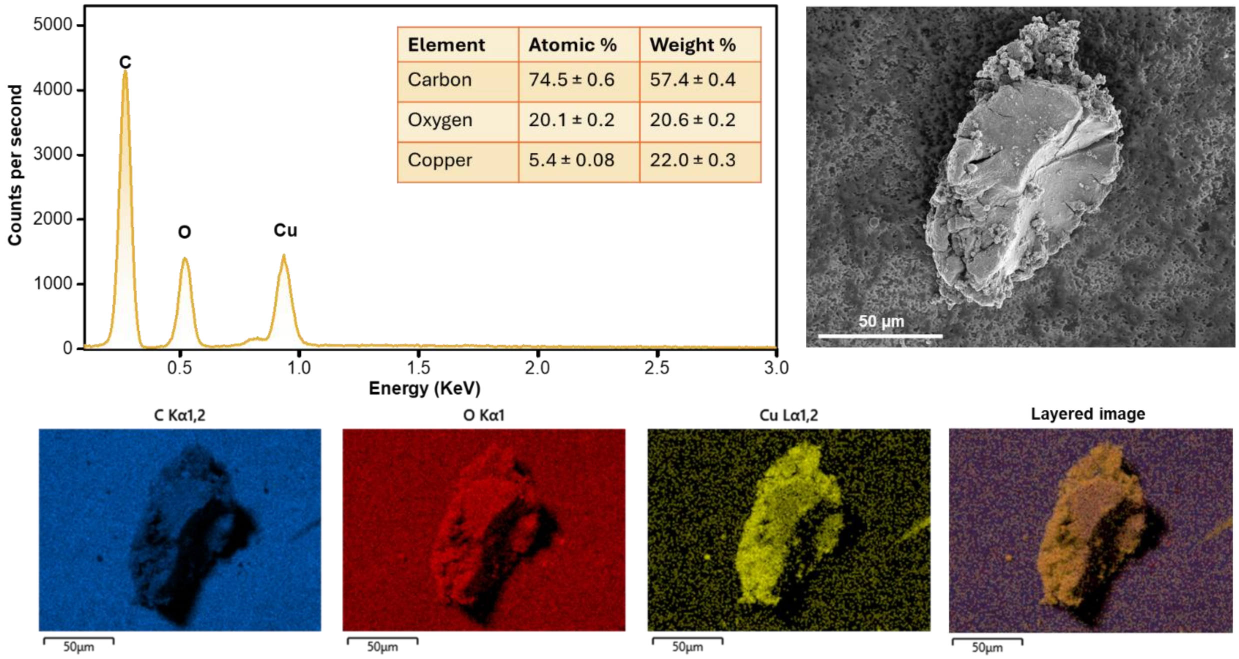

Element	Atomic %	Weight %
Carbon	74.5 ± 0.6	57.4 ± 0.4
Oxygen	20.1 ± 0.2	20.6 ± 0.2
Copper	5.4 ± 0.08	22.0 ± 0.3

Figure S10. EDX spectrum and elemental mapping images of Cu-HHTP.

2.4 Transmission electron microscope (TEM)

Transmission electron microscope (TEM) measurements were carried out on a Thermo Scientific Talos F200i instrument. MOF suspensions were first prepared by adding 0.8 mg of the corresponding MOF material to 0.6 mL of acetone in a microcentrifuge tube, followed by sonication for two hours. 20 μL of the MOF suspensions were then drop-casted onto copper grids (300 mesh, 3.0 mm O.D). The grids were placed in a vacuum oven set at 73 °C for 12 hours to remove acetone. Imaging was carried out with an operating voltage of 120 kV and working distances of 20 and 50 nm.

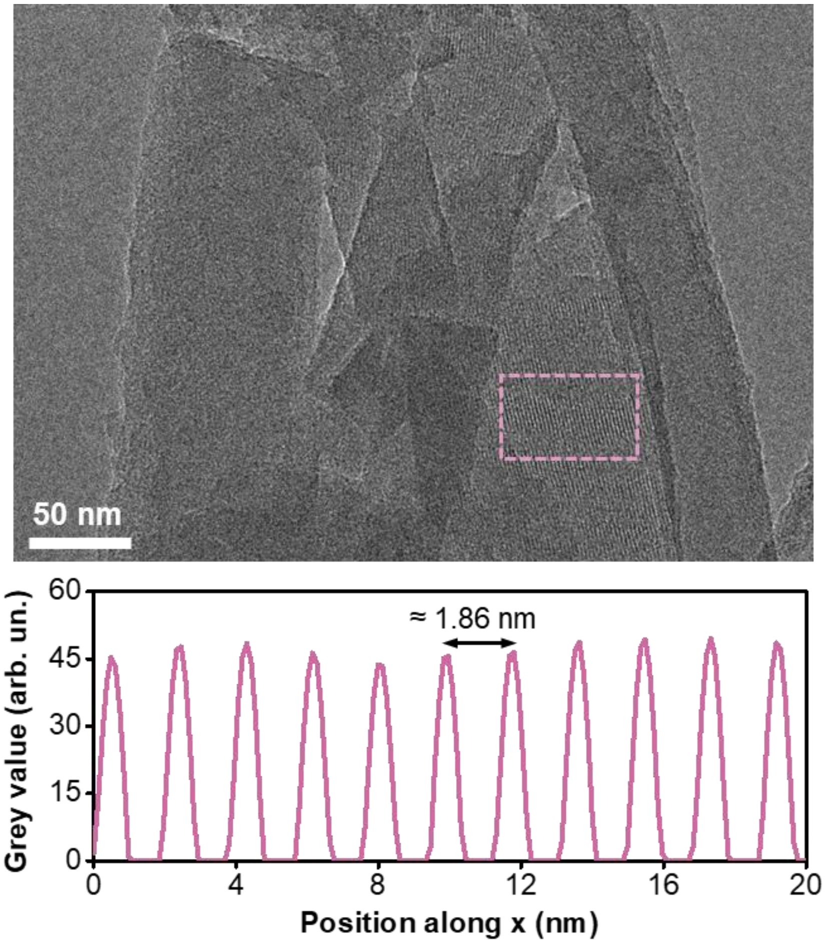

Figure S11. HR-TEM micrograph of Co-HHTP along the (100) direction (top) and line intensity profile of the lattice planes (bottom). The interplanar distance is calculated to be 1.86 ± 0.05 nm.

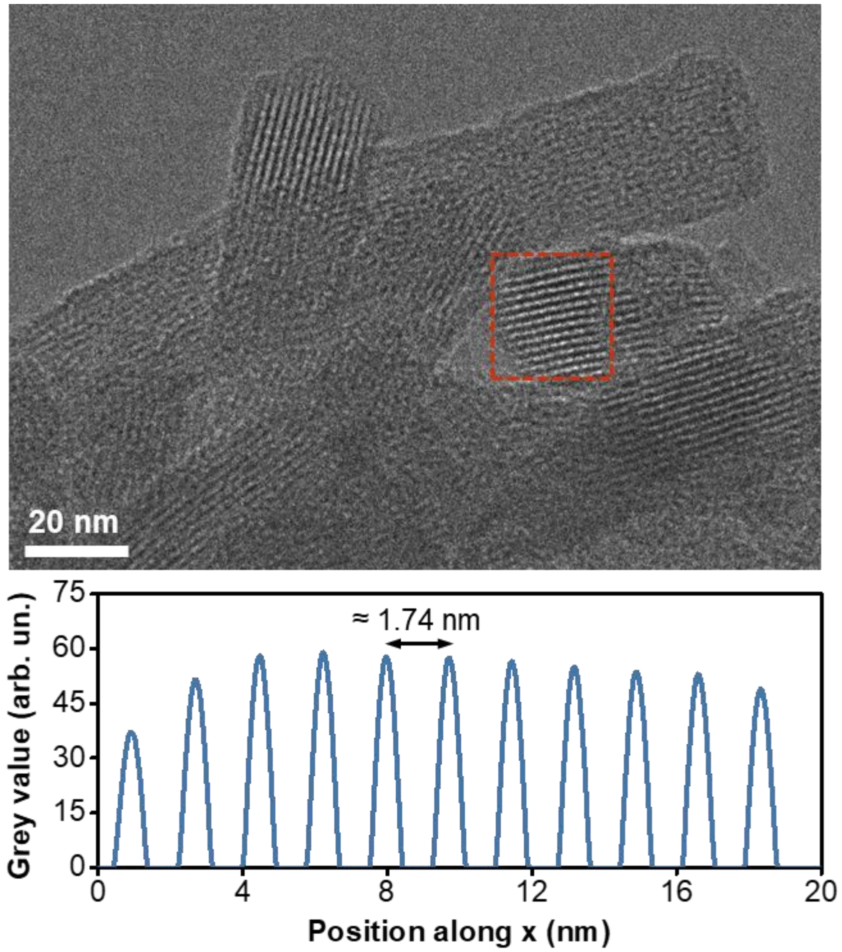

Figure S12. HR-TEM micrograph of Ni-HHTP along the (100) direction (top) and line intensity profile of the lattice planes (bottom). The interplanar distance is calculated to be 1.74 ± 0.03 nm.

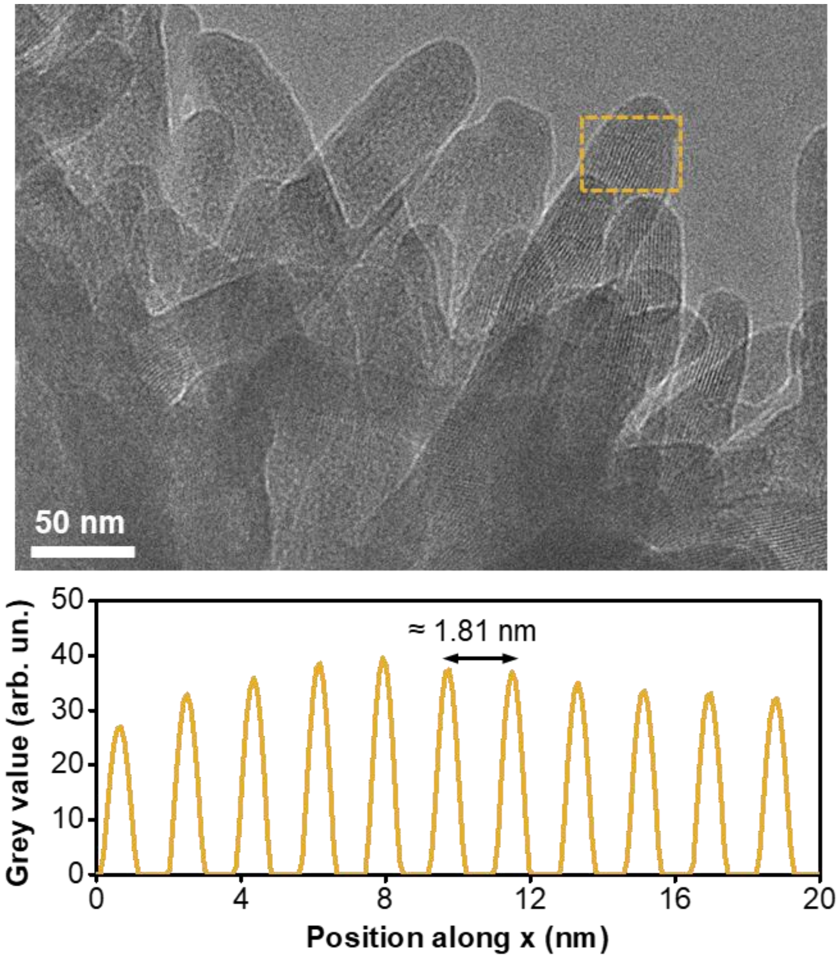

Figure S13. HR-TEM micrograph of Cu-HHTP along the (100) direction (top) and line intensity profile of the lattice planes (bottom). The interplanar distance is calculated to be 1.81 ± 0.04 nm.

2.5 Attenuated total reflectance-infrared spectroscopy (ATR-IR)

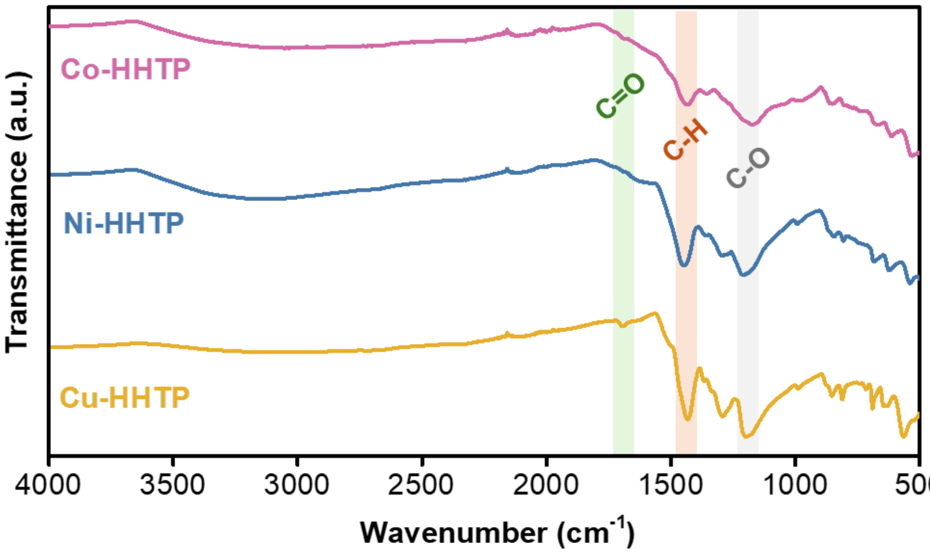

Figure S14. ATR-IR spectra of M-HHTP (M= Co, Ni, and Cu) MOF particles.

2.6 Brunauer-Emmett-Teller (BET) analysis

To assess the porosity of the frameworks, we carried out gas adsorption measurements on a 3Flex (Micromeritics, Norcross, Georgia) instrument at 77 K. All MOFs were activated using the procedure described in the main text and subsequently degassed at 100 °C under vacuum for one day prior to surface area analysis.

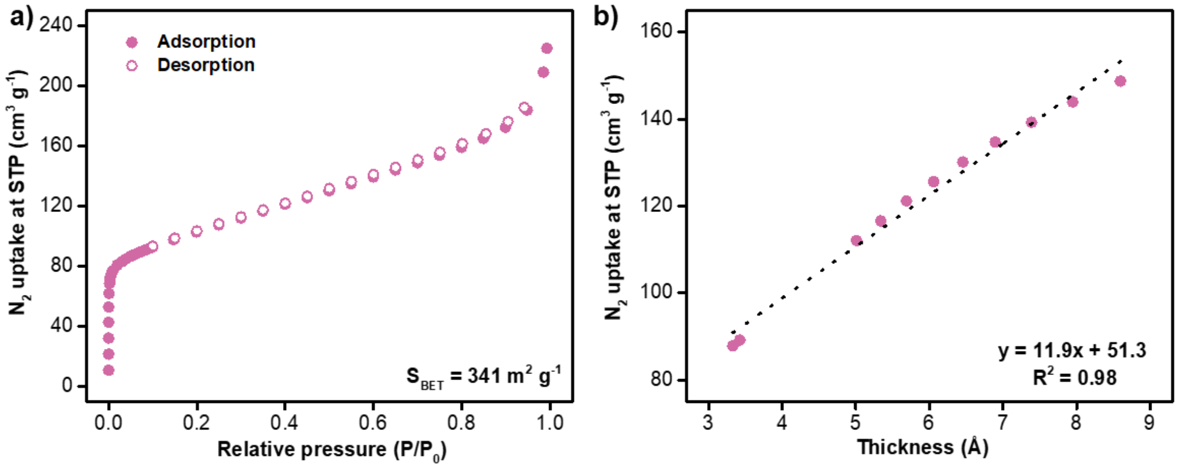

Figure S15. a) Nitrogen sorption curves (filled circles: N_2 adsorption, open circles: N_2 desorption) for Co-HHTP at standard temperature pressure (STP) and b) t-Plot for nitrogen adsorbed at 77 K by Co-HHTP. The BET surface area by the gas adsorption analysis is found to be 341 m^2 g^{-1}

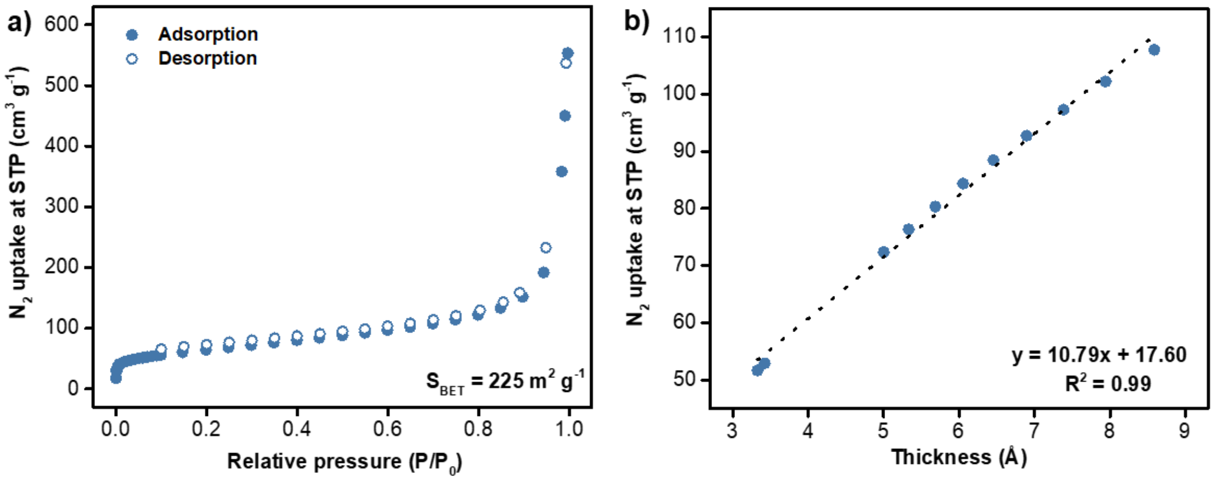

Figure S16. a) Nitrogen sorption curves (filled circles: N_2 adsorption, open circles: N_2 desorption) for Ni-HHTP at STP and b) t-Plot for nitrogen adsorbed at 77 K by Ni-HHTP. The BET surface area by the gas adsorption analysis is found to be 225 m^2 g^{-1}

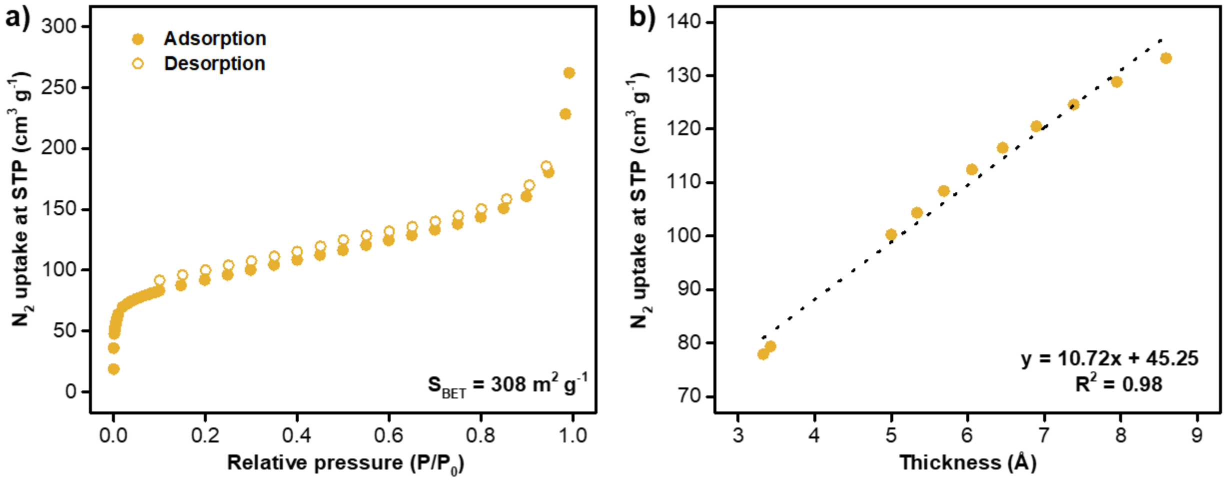

Figure S17. a) Nitrogen sorption curves (filled circles: N_2 adsorption, open circles: N_2 desorption) for Cu-HHTP at STP and b) t-Plot for nitrogen adsorbed at 77 K by Cu-HHTP. The BET surface area by the gas adsorption analysis is found to be 308 m^2 g^{-1}.

2.7 Thermogravimetric analysis (TGA)

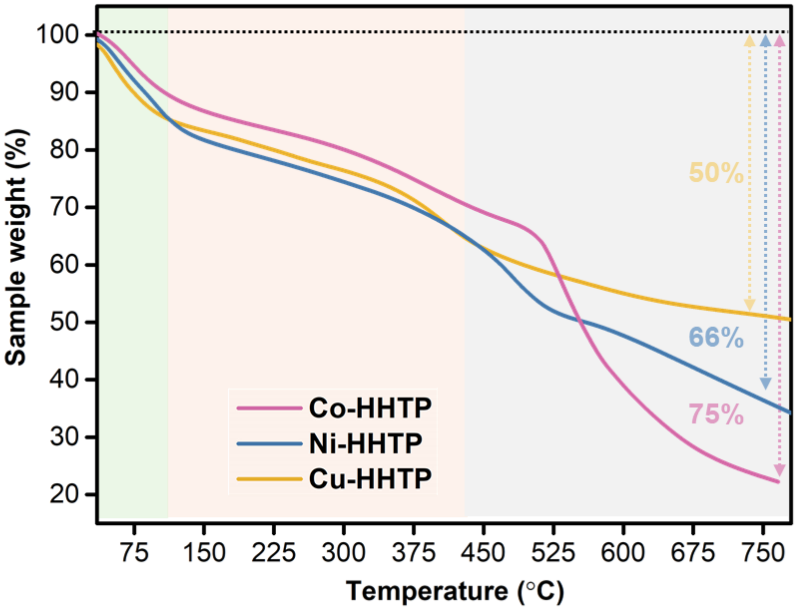

Figure S18. TGA curves of Co-HHTP, Ni-HHTP, and Cu-HHTP under nitrogen atmosphere.

2.8 Electrical conductivity measurements

We estimated the electrical conductivities of Co-HHTP, Ni-HHTP, and Cu-HHTP particles using a Signatone tungsten carbide four-point probe head with 1.25 mm spacings between each of the four tips. For that, we pressed pellets of 22 mg of each MOF material using a 6 mm inner-diameter split sleeve pressing die under a pressure of approximately 1000 psi for 15 minutes. The bulk conductivity was calculated using the equation below:

$$\sigma = \frac{I}{V}\frac{1}{2\pi sF}$$

Where σ is the bulk electrical conductivity, *I* is current (in A), *V* is the voltage across the probes, *s* is distance between probes (0.125 cm in this case), and *F* is the correction factor accounting for the diameter, height, width, and probe spacing of the pellet.[1,2]

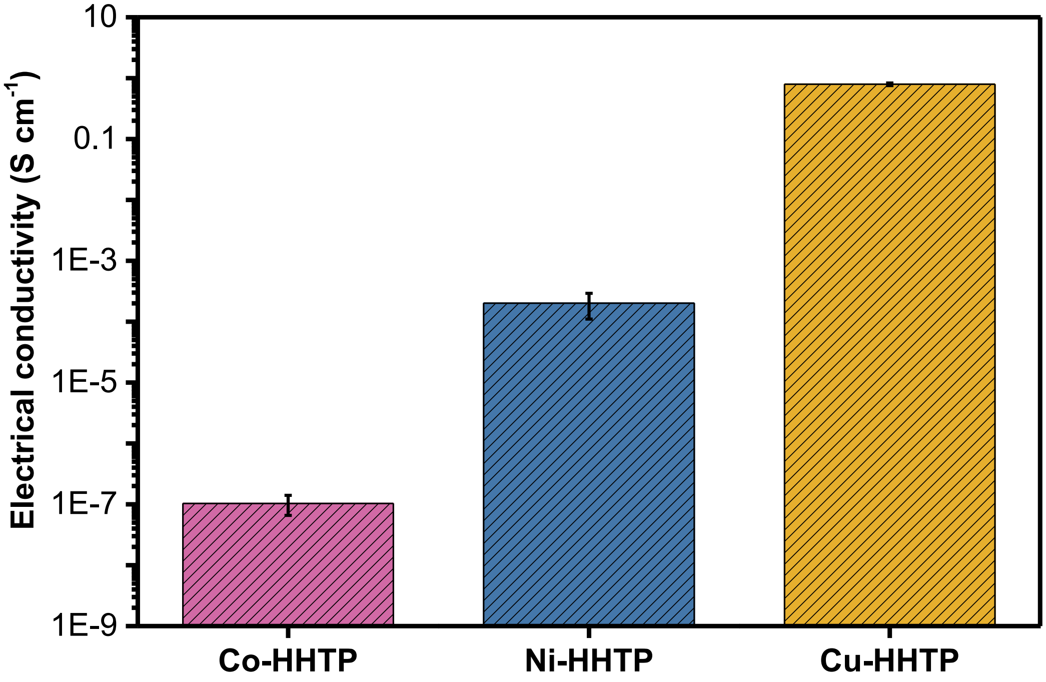

Figure S19. Bulk electrical conductivity measurements of Co-HHTP, Ni-HHTP, and Cu-HHTP pellets by the four-point probe method.

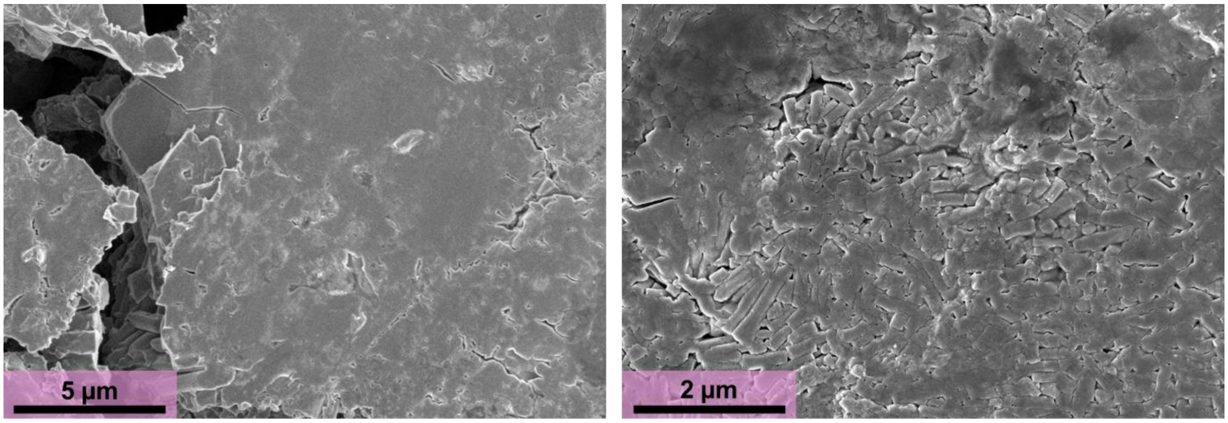

Figure S20. SEM micrographs of the Co-HHTP pellet following 4-point probe measurements.

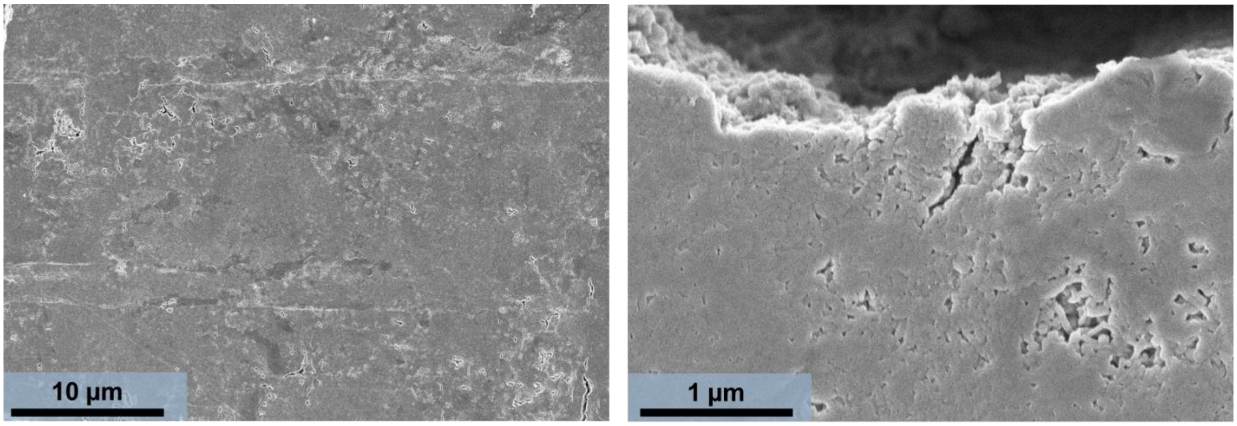

Figure S21. SEM micrographs of the Ni-HHTP pellet following 4-point probe measurements.

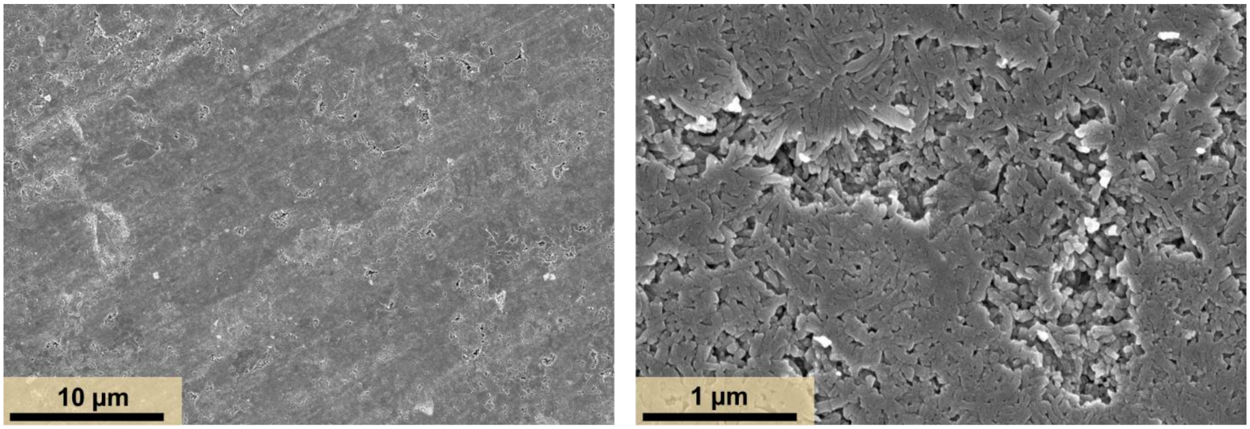

Figure S22. SEM micrographs of the Cu-HHTP pellet following 4-point probe measurements.

3. Concentration-dependent adsorption studies

Stock solutions of 500 ppm of Cd(II), Hg(II), and Pb(II) were first prepared by dissolving appropriate amounts of $CdCl_2$, $HgCl_2$, and $Pb(NO_3)_2$ powders in deionized (DI) water. To achieve varying initial concentrations of heavy metals (10–500 ppm), these stock solutions were diluted with DI water. Adsorption experiments were conducted in 8 mL glass scintillation vials, where 2 mg of activated MOF adsorbent was added to 3 mL of contaminant solution at the desired concentration. The mixtures were stirred at room temperature for 4 hours, after which the MOF powders were separated from the solutions using 0.45 μm PTFE syringe filters. The supernatants

were analyzed using inductively coupled plasma mass spectrometry (ICP-MS) to quantify the remaining concentrations of heavy metals in solution post-adsorption.

The equilibrium adsorption capacity Q_e, representing the amount of contaminants (adsorbate) adsorbed by one gram of the MOF material ($mg_{contaminant}$ $g^{-1}{}_{MOF}$) at equilibrium was determined using **Equation S1** as follows:

$$Q_e(mg\ g^{-1}) = \frac{C_0 - C_e}{m} \times V \qquad \textbf{(Equation S1)}$$

Where C_0 is the initial concentration of contaminant (in ppm), C_e is the concentration of contaminant remaining in solution after adsorption (in ppm), *V* is the volume of the solution in which the pollutant is dissolved in (in mL), and *m* is the mass of the MOF sample used (in mg).

3.1 Adsorption isotherm models

3.1.1 Langmuir adsorption model

The Langmuir adsorption model assumes a homogeneous surface where the available adsorption active sites within the MOF material are equal in energy. The ionic contaminants in this case get adsorbed on the surface of the MOF through a monolayer adsorption process with no interactions between adsorbed molecules. Therefore, the adsorbent has a limited capacity to adsorb ions, where no further adsorption can take place once the adsorption site is fully occupied.

We fitted the experimental adsorption data to the linear Langmuir adsorption model according to **Equation S2**.

$$\frac{C_e}{Q_e} = \frac{C_e}{Q_{max}} + \frac{1}{K_L Q_{max}} \qquad \textbf{(Equation S2)}$$

Where Q_{max} is the theoretical saturation capacity, representing the maximum amount of adsorbate (lead, mercury, and cadmium) that can be adsorbed by one gram of the MOF materials (in mg g-

^{1}), and K_L (L mg^{-1}) is the Langmuir adsorption constant, which reflects the affinity between the adsorbent and adsorbate.

3.1.2 Freundlich adsorption model

As opposed to the Langmuir model, the Freundlich adsorption model assumes a heterogeneous surface where the adsorbent possesses a range of binding sites with distinct energies. Given the model describing a multilayer adsorption process, it is possible to form multiple adsorption layers of adsorbate on the adsorbent surface, suggesting no saturation point and therefore no ability to determine Q_{max} from the model.

We fitted the experimental adsorption data to the linear Freundlich adsorption model according to **Equation S3**.

$$ln\,(Q_e) = ln\,(K_F) + \frac{1}{n} ln\,(C_e) \qquad \textbf{(Equation S3)}$$

Where K_F ((mg g^{-1})(L mg^{-1})$^{1/n}$) is the Freundlich adsorption constant, which reflects the effectiveness of the adsorption capacity of the adsorbent and 1/n (unitless)n is the Freundlich exponent, which reflects the surface heterogeneity of the adsorbent.

3.1.3 Temkin adsorption model

Similar to the Freundlich model, the Temkin adsorption model assumes a heterogeneous surface. However, it is characterized by a uniform distribution of binding sites and describes the interactions between adsorbed molecules on the surface of the adsorbate.

We fitted the experimental adsorption data to the linear Temkun adsorption model according to **Equation S4**.

$$Q_e = \frac{RT}{b}\ln(C_e) + \frac{RT}{b}\ln\,(K_t) \qquad \textbf{(Equation S4)}$$

Where R is the universal gas constant (8.314 j mol^{-1} K^{-1}), T is the temperate (in K), b is a constant related to the heat of adsorption, and A is the Temkin equilibrium binding constant, which reflects the strength of the interactions between both the contaminants and the MOF.

3.1.4 Dubinin-Radushkevich (D-R) adsorption model

While initially developed to describe the adsorption process of gaseous analytes onto microporous sorbents,[3] the D-R adsorption model has been recently applied to solid-liquid interfaces to provide insights into the adsorption mechanism (physical vs chemical adsorption) by considering both, the adsorption potential and the nature of the adsorption sites on the adsorbate.[4]

We fitted the experimental adsorption data to the linear D–R adsorption model according to **Equation S5**.

$$\ln(Q_e) = \ln(Q_{max}) - K\varepsilon^2 \qquad \textbf{(Equation S5)}$$

Where K is the activity coefficient, which reflects the mean free energy of adsorption on the adsorbate, and ε is the Polanyi potential, which reflects the potential energy of adsorption, and is given by **Equation S6** as follows:

$$\varepsilon = RTln\left(1 + \frac{1}{C_e}\right) \qquad \textbf{(Equation S6)}$$

To determine whether the ion adsorption behavior is governed by chemisorption or physisorption, we calculated the mean free energy of adsorption per molecule of adsorbate (E) according to **Equation S7**.

$$E = \frac{1}{\sqrt{2K}} \qquad \textbf{(Equation S7)}$$

Where an E < 8 Kj mol^{-1} is indicative of a physical adsorption process while an E > 8 Kj mol^{-1} is indicative of a chemical adsorption process.

3.2 Adsorption isotherms of lead ions (Pb^{2+})

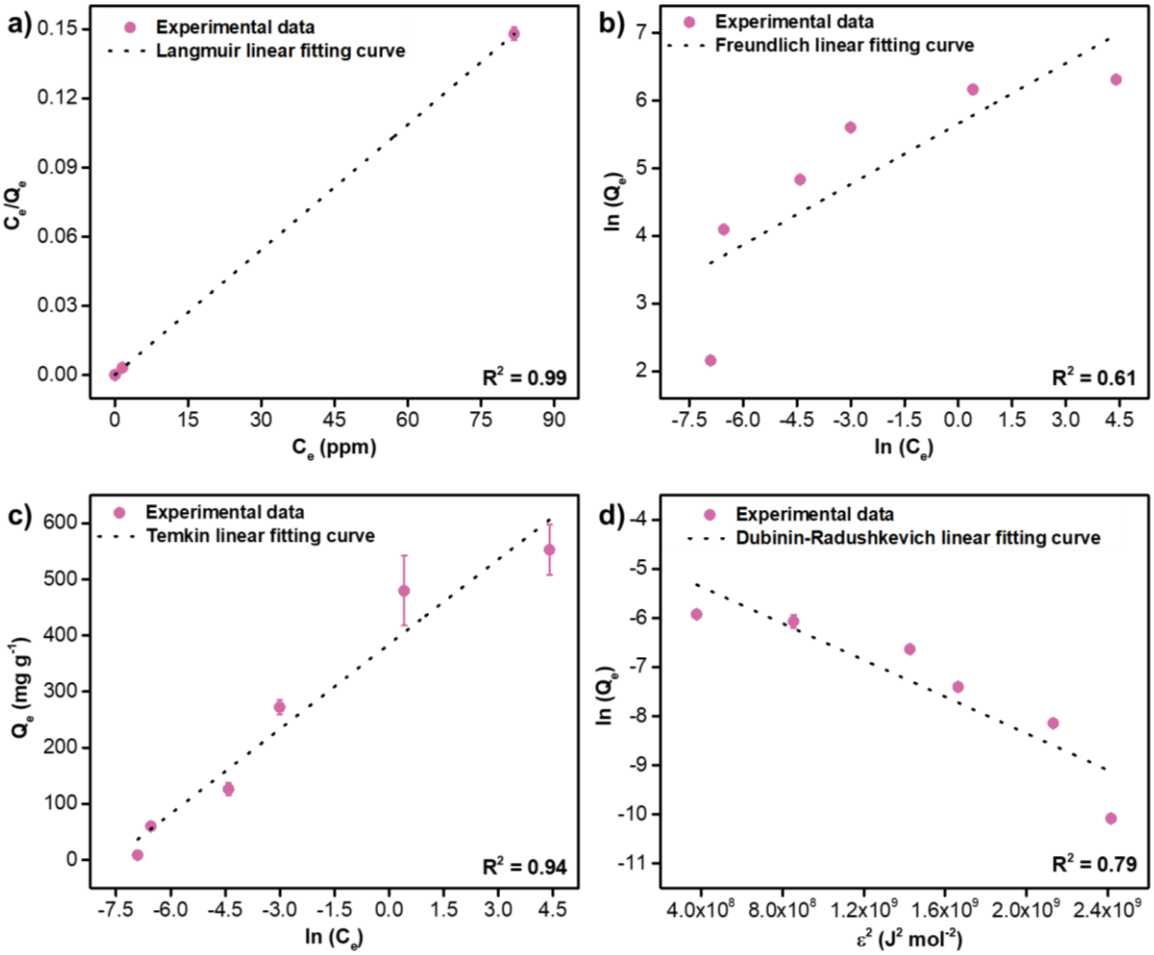

Figure S23. Experimental adsorption data fitting using a) Langmuir (**Equation S2**), b) Freundlich (**Equation S3**), c) Temkin (**Equation S4**), and d) Dubinin-Radushkevich (D-R) (**Equation S5**) linear models of Pb(II) adsorption isotherms onto Co-HHTP. Conditions: m_{MOF} = 2 mg, $V_{solution}$ = 3 mL, and T = 298 K under a contact time of 4 hours. Error bars represent standard deviation from the mean value of three independent experiments.

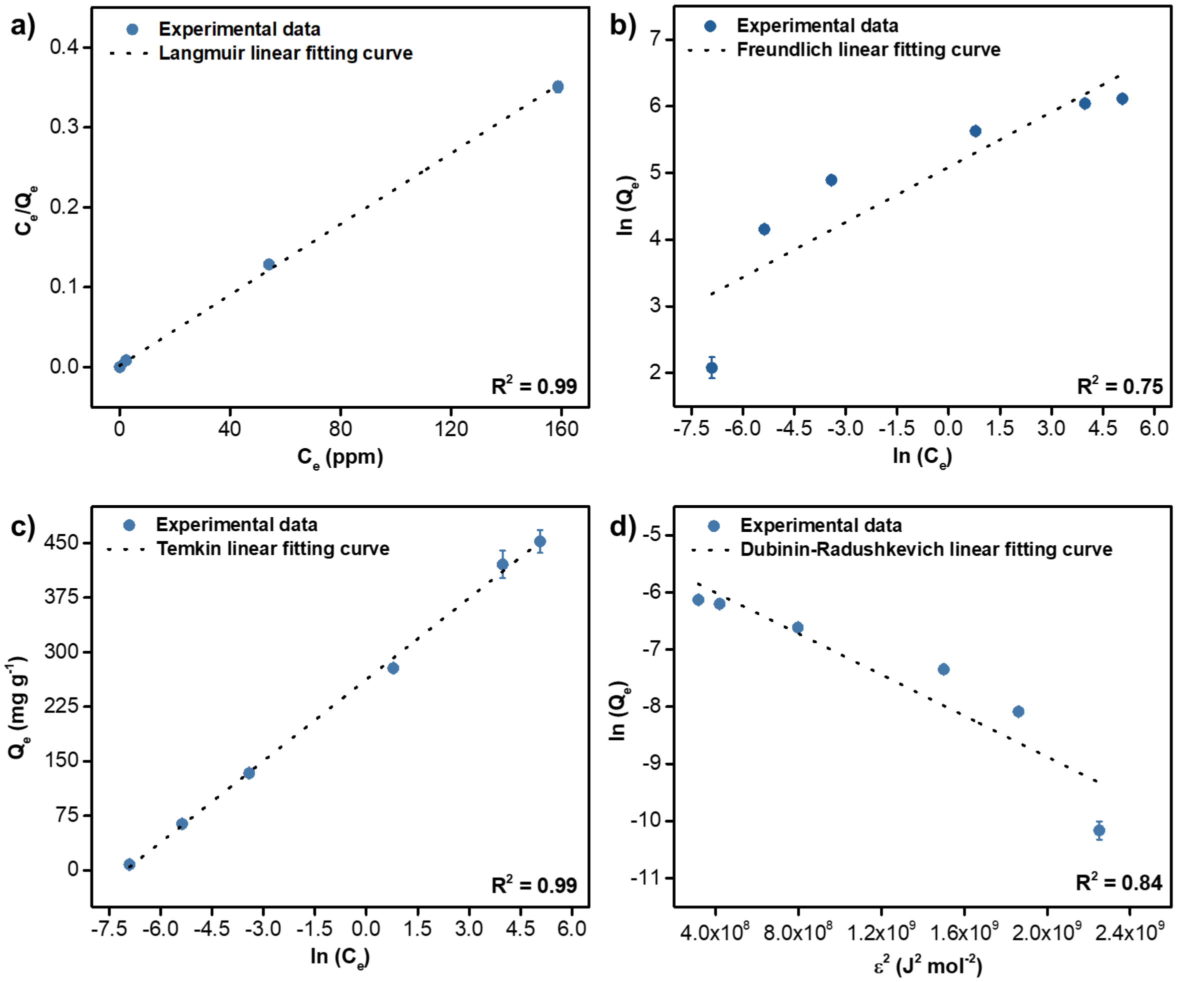

Figure S24. Experimental adsorption data fitting using a) Langmuir (**Equation S2**), b) Freundlich (**Equation S3**), c) Temkin (**Equation S4**), and d) Dubinin-Radushkevich (D-R) (**Equation S5**) linear models of Pb(II) adsorption isotherms onto Ni-HHTP. Conditions: m_{MOF} = 2 mg, $V_{solution}$ = 3 mL, and T = 298 K under a contact time of 4 hours. Error bars represent standard deviation from the mean value of three independent experiments.

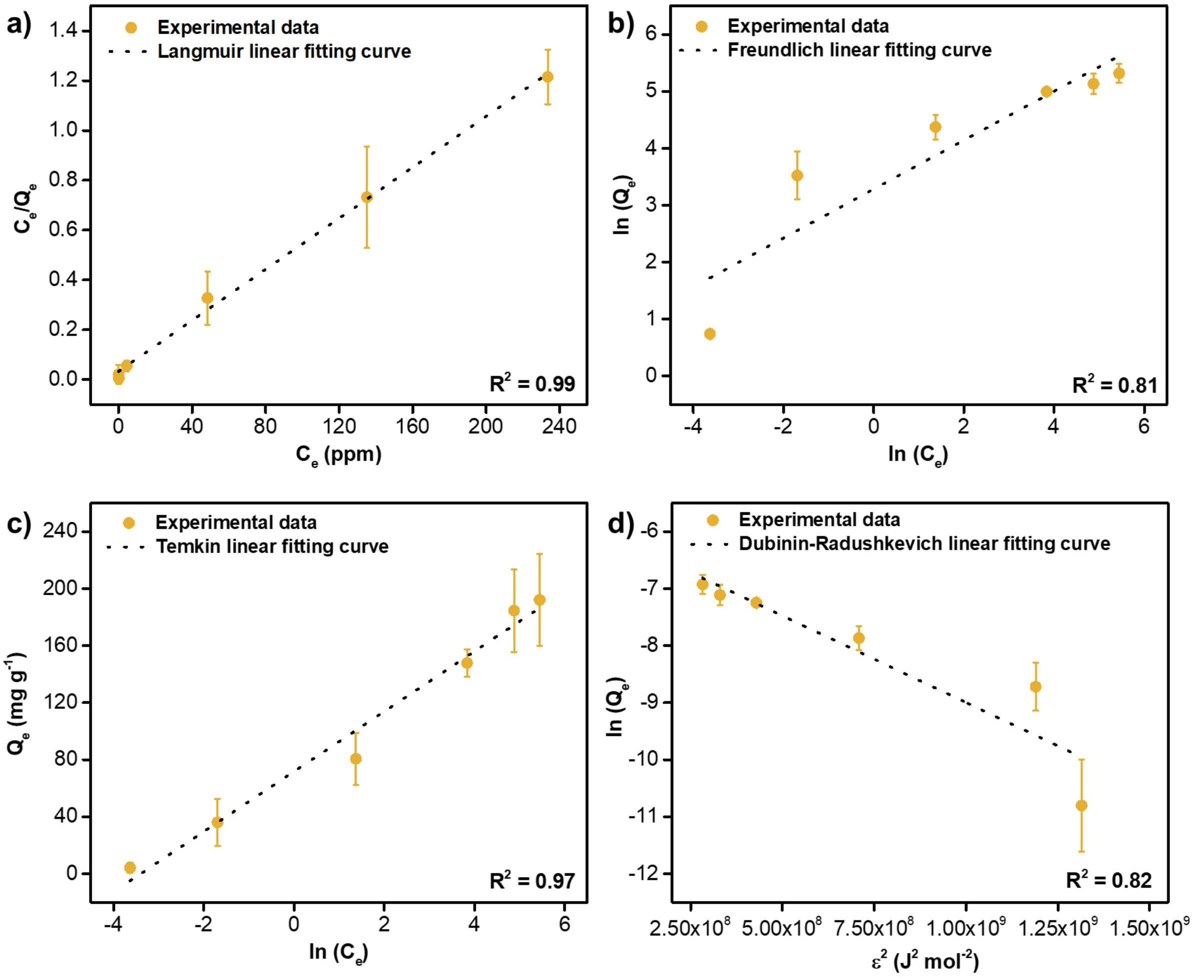

Figure S25. Experimental adsorption data fitting using a) Langmuir (**Equation S2**), b) Freundlich (**Equation S3**), c) Temkin (**Equation S4**), and d) Dubinin-Radushkevich (D-R) (**Equation S5**) linear models of Pb(II) adsorption isotherms onto Cu-HHTP. Conditions: m_{MOF} = 2 mg, $V_{solution}$ = 3 mL, and T = 298 K under a contact time of 4 hours. Error bars represent standard deviation from the mean value of three independent experiments.

Parameters	Co-HHTP	Ni-HHTP	Cu-HHTP
**Langmuir Isotherm**			
**Q_{max} (mg g^{-1})**	552.5	452.5	195.3
**K_L (L/mg)**	12.1	1.1	0.2
**Freundlich isotherm**			
**K_F (mg g^{-1})**	286.9	162.0	26.6
**n**	3.3	3.6	2.3
**Temkin isotherm**			
**B (J mol^{-1})**	20.2	56.9	123.4
**K_T (L mg^{-1})**	15.3	36.1	100.0
**Dubinin-Radushkevich isotherm**			
**K (mol^2 j^{-2})**	1.9 x 10^{-9}	1.8 x 10^{-9}	3.0 x 10^{-9}
**E (Kj mol^{-1})**	16.3	16.7	12.9

Table S1. Linear equilibrium modelling parameters for the adsorption of Pb(II) onto Co-HHTP, Ni-HHTP, and Cu-HHTP MOFs.

3.3 Adsorption isotherms of cadmium ions (Cd^{2+})

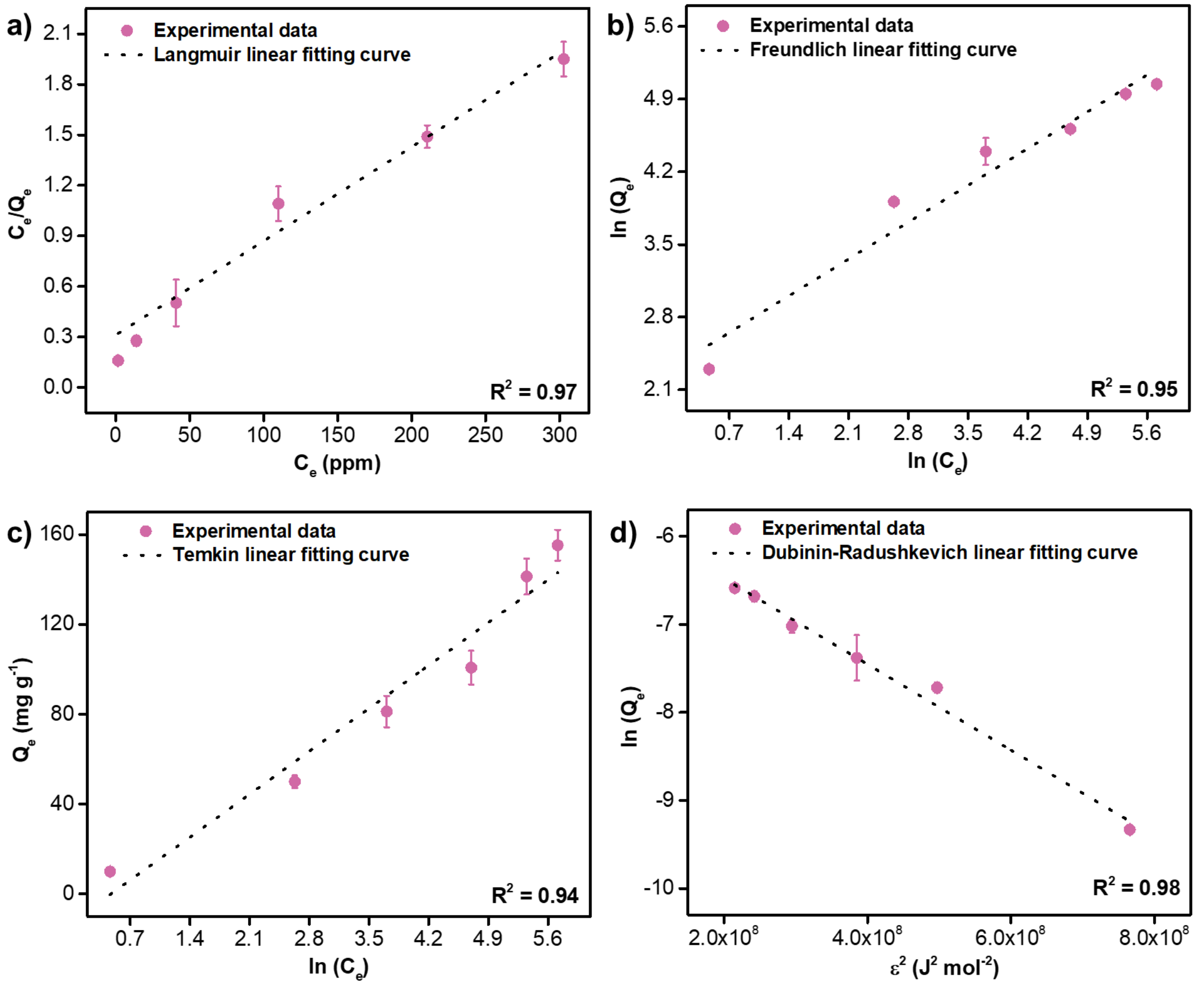

Figure S26. Experimental adsorption data fitting using a) Langmuir (**Equation S2**), b) Freundlich (**Equation S3**), c) Temkin (**Equation S4**), and d) Dubinin-Radushkevich (D-R) (**Equation S5**) linear models of Cd(II) adsorption isotherms onto Co-HHTP. Conditions: m_{MOF} = 2 mg, $V_{solution}$ = 3 mL, and T = 298 K under a contact time of 4 hours. Error bars represent standard deviation from the mean value of three independent experiments.

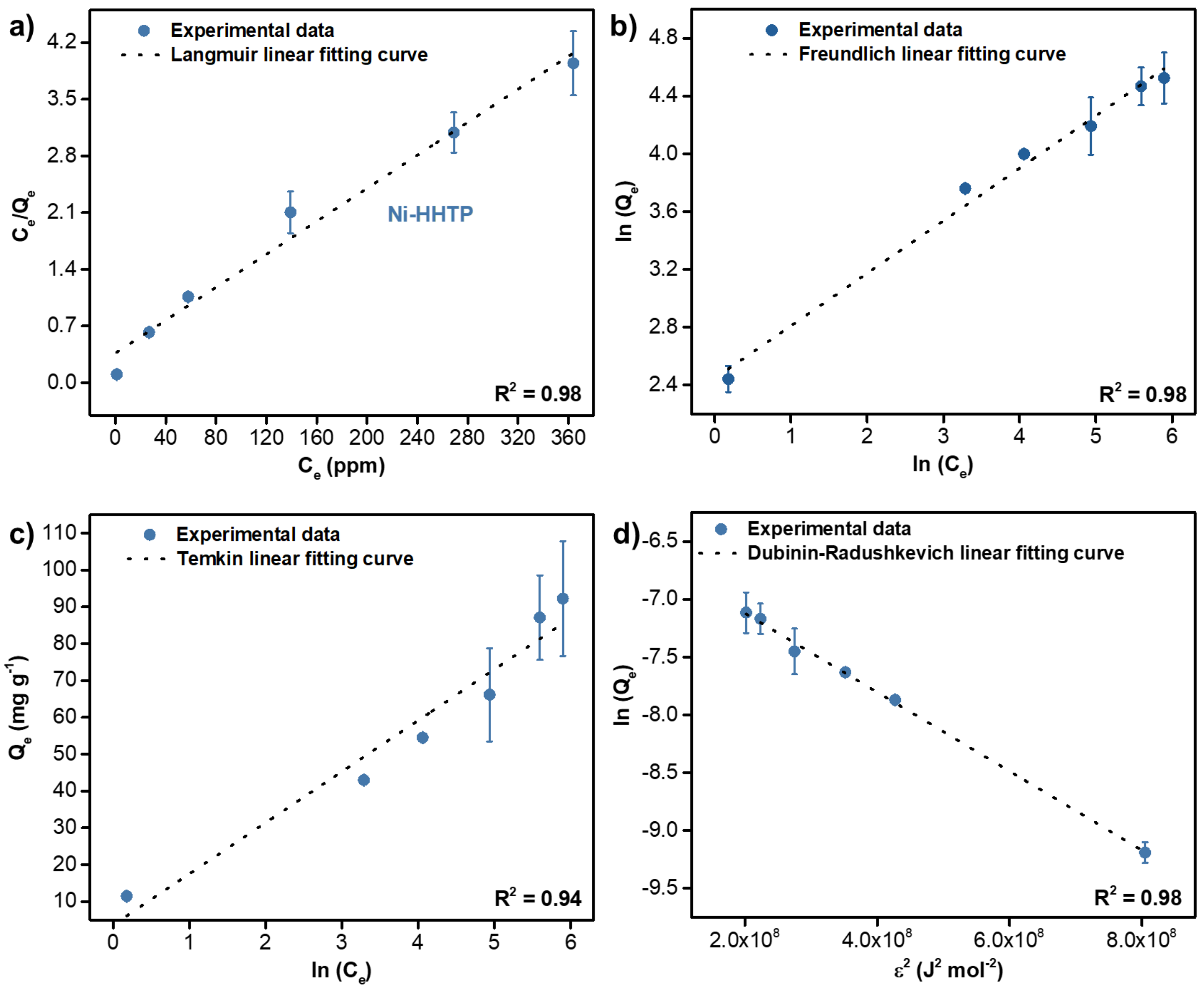

Figure S37. Experimental adsorption data fitting using a) Langmuir (**Equation S2**), b) Freundlich (**Equation S3**), c) Temkin (**Equation S4**), and d) Dubinin-Radushkevich (D-R) (**Equation S5**) linear models of Cd(II) adsorption isotherms onto Ni-HHTP. Conditions: m_{MOF} = 2 mg, $V_{solution}$ = 3 mL, and T = 298 K under a contact time of 4 hours. Error bars represent standard deviation from the mean value of three independent experiments.

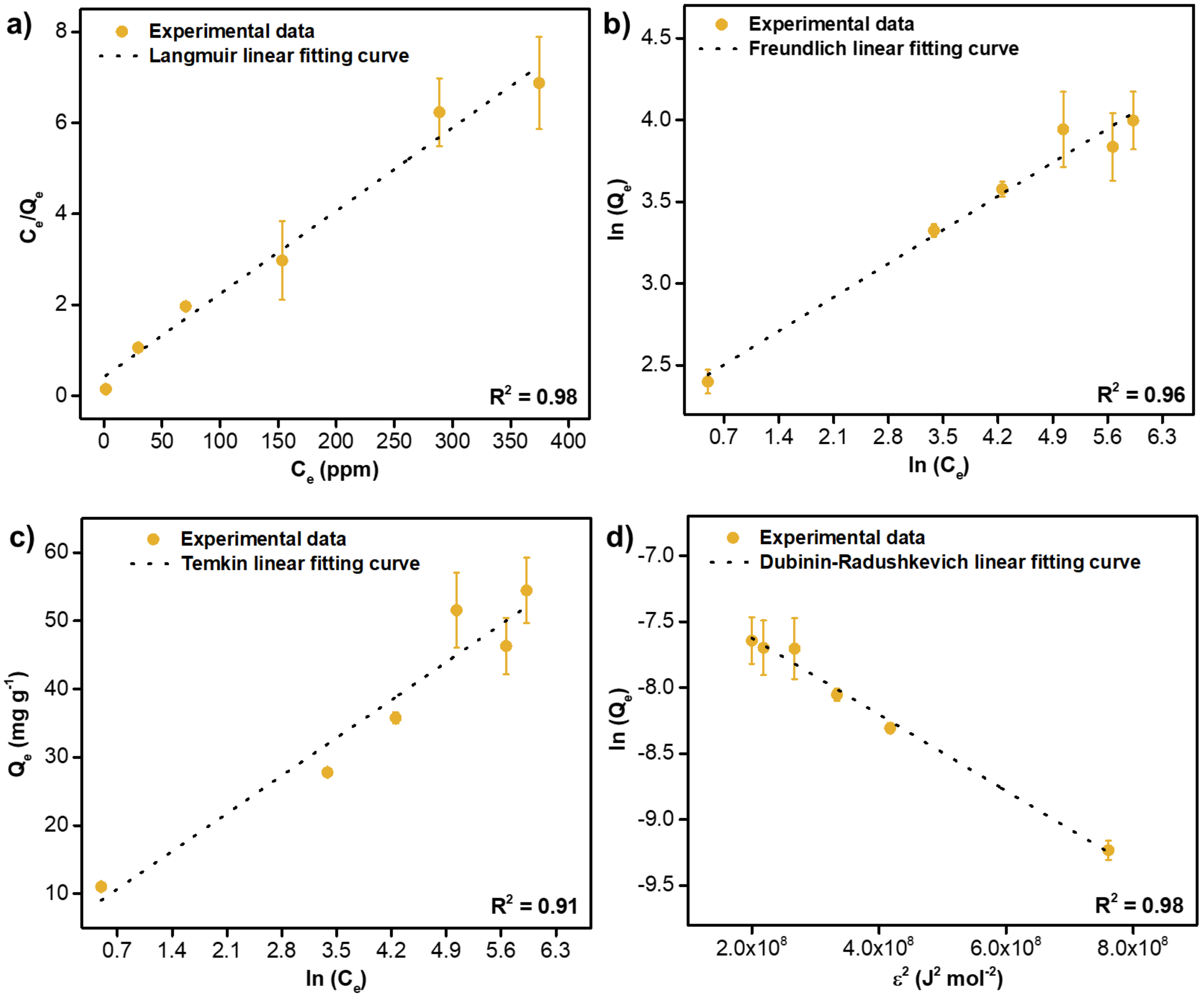

Figure S28. Experimental adsorption data fitting using a) Langmuir (**Equation S2**), b) Freundlich (**Equation S3**), c) Temkin (**Equation S4**), and d) Dubinin-Radushkevich (D-R) (**Equation S5**) linear models of Cd(II) adsorption isotherms onto Cu-HHTP. Conditions: m_{MOF} = 2 mg, $V_{solution}$ = 3 mL, and T = 298 K under a contact time of 4 hours. Error bars represent standard deviation from the mean value of three independent experiments.

Parameters	Co-HHTP	Ni-HHTP	Cu-HHTP
**Langmuir Isotherm**			
**Q_{max} (mg g^{-1})**	179.2	98.2	54,7
**K_L (L/mg)**	0.02	0.03	0.04
**Freundlich isotherm**			
**K_F (mg g^{-1})**	9.9	11.5	9.9
**n**	2.0	2.7	3.4
**Temkin isotherm**			
**B (J mol^{-1})**	90.8	178.4	312.7
**K_T (L mg^{-1})**	0.6	1.3	1.9
**Dubinin-Radushkevich isotherm**			
**K (mol^2 j^{-2})**	4.9×10^{-9}	3.4×10^{-9}	2.9×10^{-9}
**E (Kj mol^{-1})**	10.2	12.1	13.1

Table S2. Linear equilibrium modelling parameters for the adsorption of Cd(II) onto Co-HHTP, Ni-HHTP, and Cu-HHTP MOFs.

3.4 Adsorption isotherms of mercury ions (Hg^{2+})

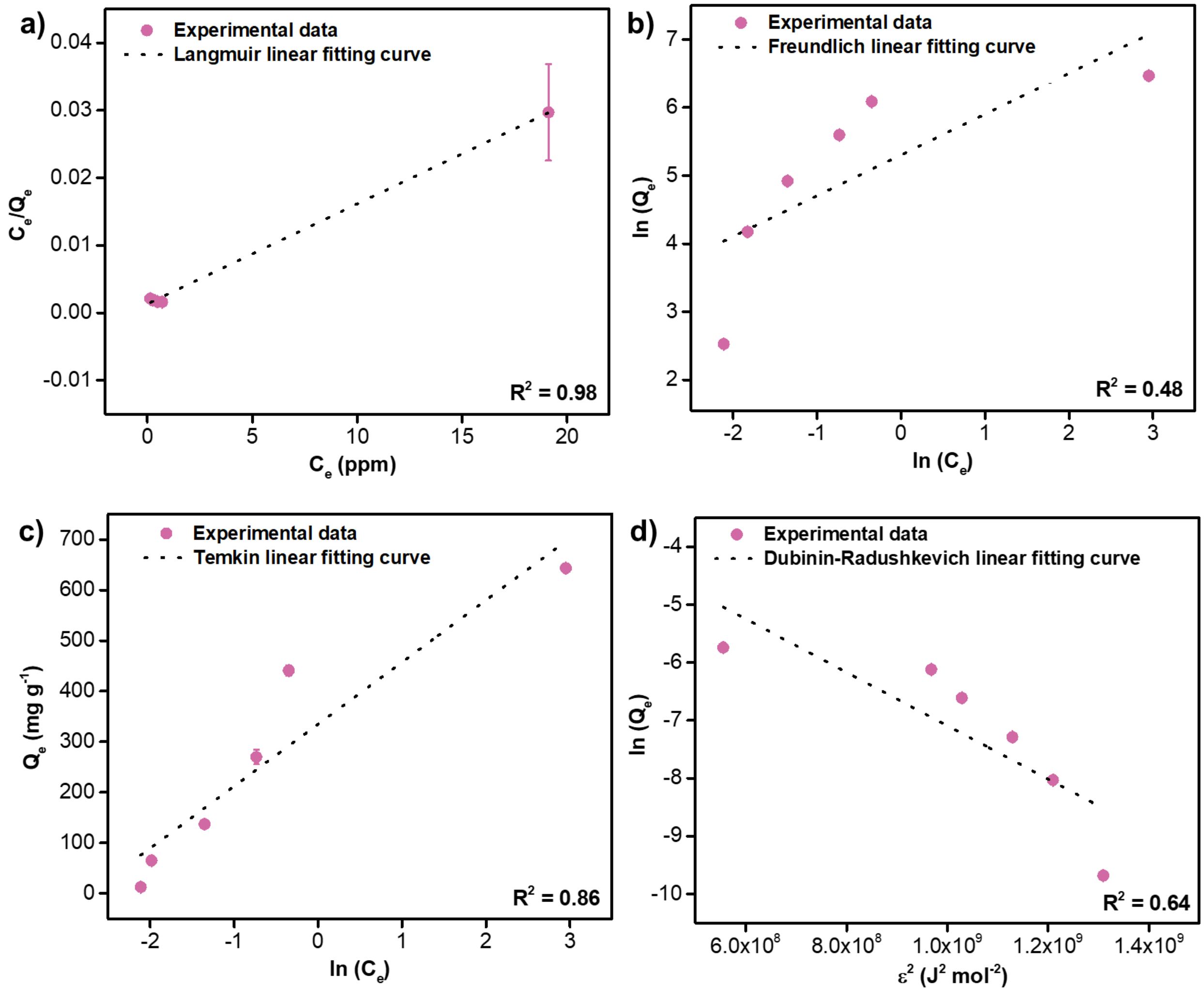

Figure S29. Experimental adsorption data fitting using a) Langmuir (**Equation S2**), b) Freundlich (**Equation S3**), c) Temkin (**Equation S4**), and d) Dubinin-Radushkevich (D-R) (**Equation S5**) linear models of Hg(II) adsorption isotherms onto Co-HHTP. Conditions: m_{MOF} = 2 mg, $V_{solution}$ = 3 mL, and T = 298 K under a contact time of 4 hours. Error bars represent standard deviation from the mean value of three independent experiments.

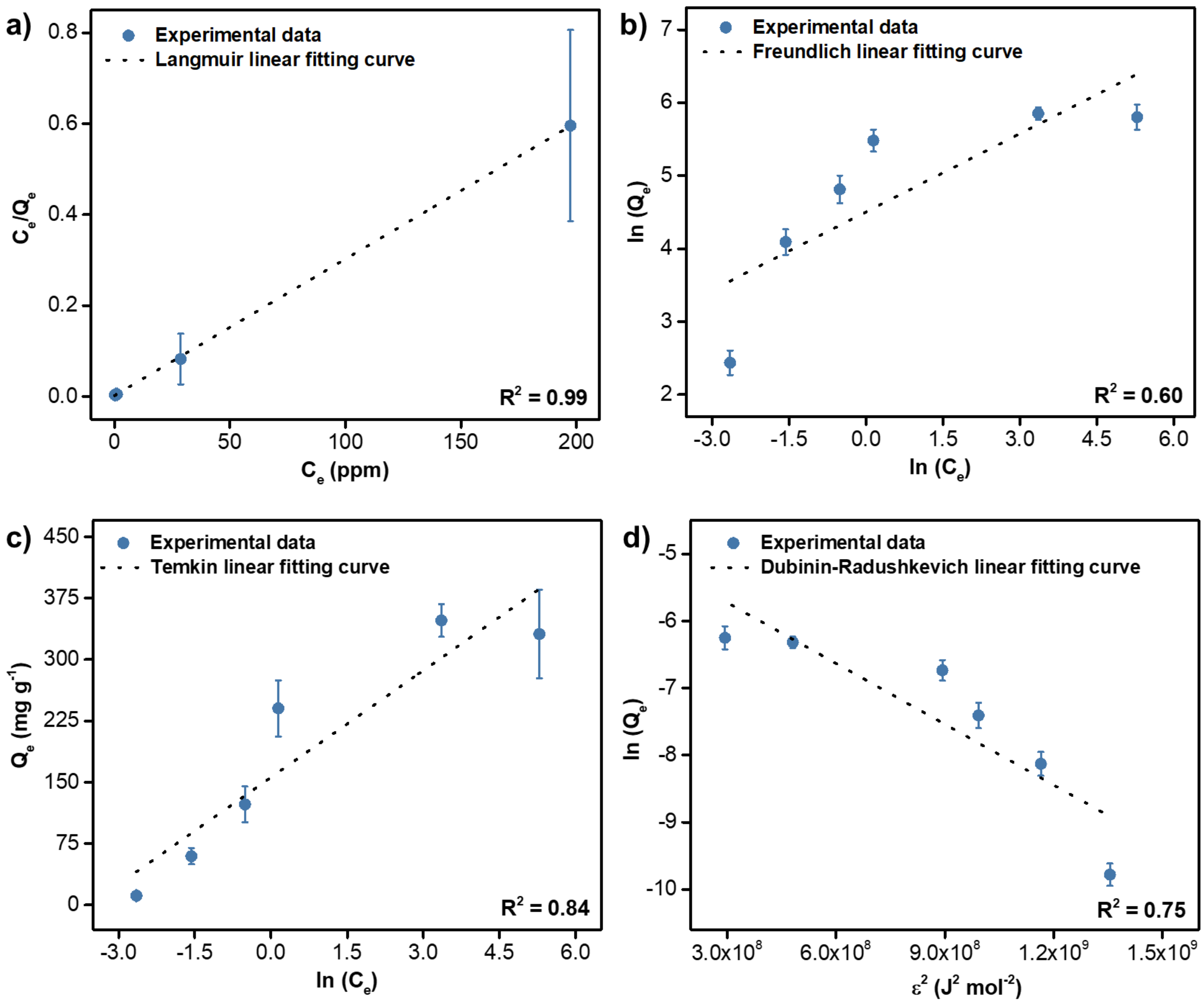

Figure S30. Experimental adsorption data fitting using a) Langmuir (**Equation S2**), b) Freundlich (**Equation S3**), c) Temkin (**Equation S4**), and d) Dubinin-Radushkevich (D-R) (**Equation S5**) linear models of Hg(II) adsorption isotherms onto Ni-HHTP. Conditions: m_{MOF} = 2 mg, $V_{solution}$ = 3 mL, and T = 298 K under a contact time of 4 hours. Error bars represent standard deviation from the mean value of three independent experiments.

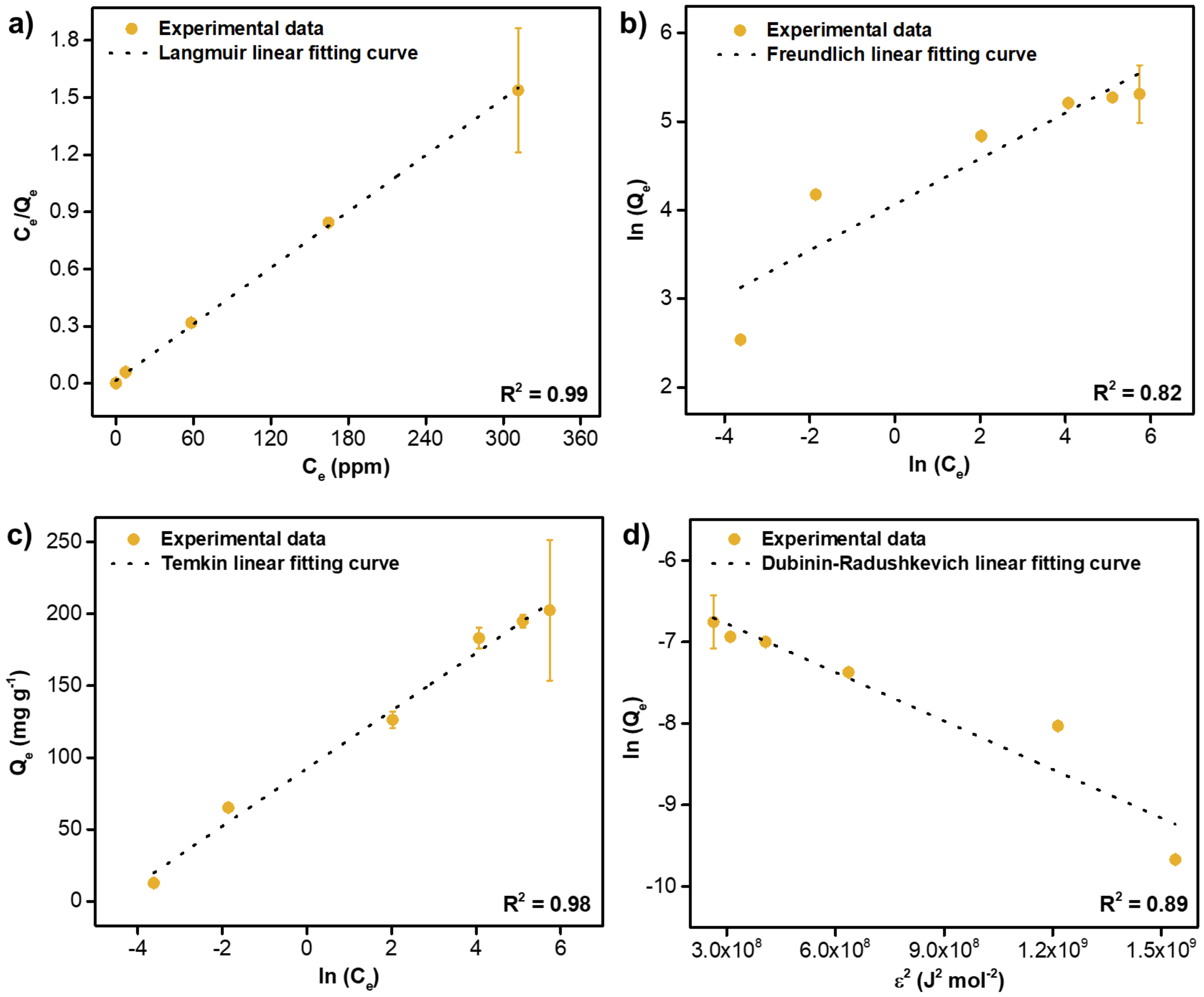

Figure S31. Experimental adsorption data fitting using a) Langmuir (**Equation S2**), b) Freundlich (**Equation S3**), c) Temkin (**Equation S4**), and d) Dubinin-Radushkevich (D-R) (**Equation S5**) linear models of Hg(II) adsorption isotherms onto Cu-HHTP. Conditions: m_{MOF} = 2 mg, $V_{solution}$ = 3 mL, and T = 298 K under a contact time of 4 hours. Error bars represent standard deviation from the mean value of three independent experiments.

Parameters	Co-HHTP	Ni-HHTP	Cu-HHTP
		**Langmuir Isotherm**	
**Q_{max} (mg g^{-1})**	675.7	334.0	202.8
**K_L (L/mg)**	1.1	1.4	0.3
		**Freundlich isotherm**	
**K_F (mg g^{-1})**	201.5	90.4	58.2
**n**	1.7	2.8	3.9
		**Temkin isotherm**	
**B (J mol^{-1})**	20.2	56.9	123.4
**K_T (L mg^{-1})**	15.3	36.1	100.0
		**Dubinin-Radushkevich isotherm**	
**K (mol^2 j^{-2})**	4.6 x 10^{-9}	3.0 x 10^{-9}	2.0 x 10^{-9}
**E (Kj mol^{-1})**	10.4	12.9	15.8

Table S3. Linear equilibrium modelling parameters for the adsorption of Hg(II) onto Co-HHTP, Ni-HHTP, and Cu-HHTP MOFs

3.5 Uptake capacities for Co-HHTP compared to reported MOF-based adsorbents

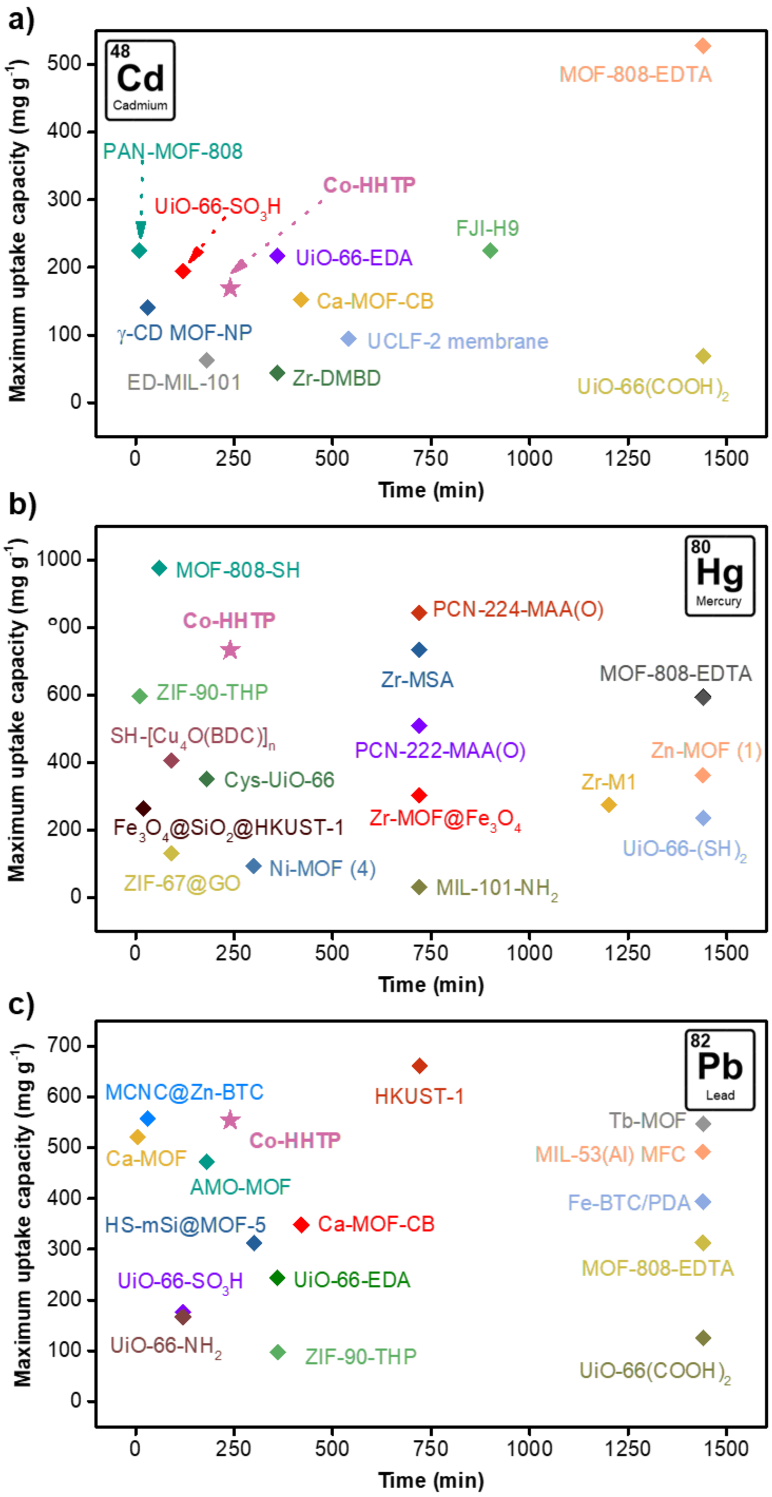

Figure S32. Comparison of maximum adsorption capacities achieved for a) Cd(II), b) Hg(II), and c) Pb(II) using MOFs-based adsorbents reported in the literature.

While Co-HHTP does not exhibit the highest absolute adsorption capacities reported for any individual heavy metal, this work distinguishes itself from other studies in several key aspects that collectively provide a clear advantage over other MOF-based adsorbents. First, it demonstrates that a single class of adsorbents (HHTP- and HITP-based cMOFs) can effectively capture both, toxic anions (oxyanions) and cations (heavy metals), with varying charge densities and sizes, using the same framework, and achieve relatively high removal efficiencies with fast adsorption kinetics. This multifunctionality is in contrast to most other MOFs, which typically require distinct functionalization strategies to target specific contaminants.[5] Second, Co-HHTP combines multiple functionalities in a single material, enabling simultaneous adsorption, detoxification (via redox-active MOF-heavy metal interactions), and detection of pollutants, whereas most other MOFs are limited to one or two of these capabilities. Third, the triphenylene-based MOFs are accessible through a simple, one-pot synthetic route using commercially available and synthetically accessible precursors, without the need for post-synthetic modifications or pre-treatment. This straightforward approach enhances scalability and facilitates practical implementation. Fourth, the MOF adsorbents can be directly deposited onto textiles using a one-pot solvothermal approach, retaining comparable adsorption performance to the bulk material, while enabling easy deployment, recovery, and reuse in practical water treatment settings. Taken together, the combination of broad-spectrum adsorption, multifunctionality, facile synthesis, and textile integration positions Co-HHTP, and triphenylene-based cMOFs in general, as uniquely promising candidates for practical, next-generation water purification technologies.

4. Synthesis, characterization, and adsorptive performance of Co-HITP

4.1 Bottom-up synthetic procedure of Co-HITP

Co-HITP was prepared according to a previously reported procedure.[6] In brief, 1.5 mL of a cobalt(II) nitrate hexahydrate solution (62.1 mmol, 10 eq) in DMF was preheated on a hot plate

set at 65 °C for 15 minutes. 328 mg of NaOAc, dissolved in 2 mL of DI water was added to the solution, followed immediately by 5 mg (0.0093 mmol, 1 eq) of 2,3,6,7,10,11-Hexaaminotriphenylene (0.04 mmol, 1 eq). the resulting mixture was loosely capped and heated while stirring on a hot plate set at 65 °C for 3 hours. The resulting black powder was filtered, washed with DI water (20 mL), methanol (40 mL), and acetone (20 mL), before being dried in a vacuum oven set at 75 °C for 32 hours.

4.2 Structural and morphological features of Co-HITP

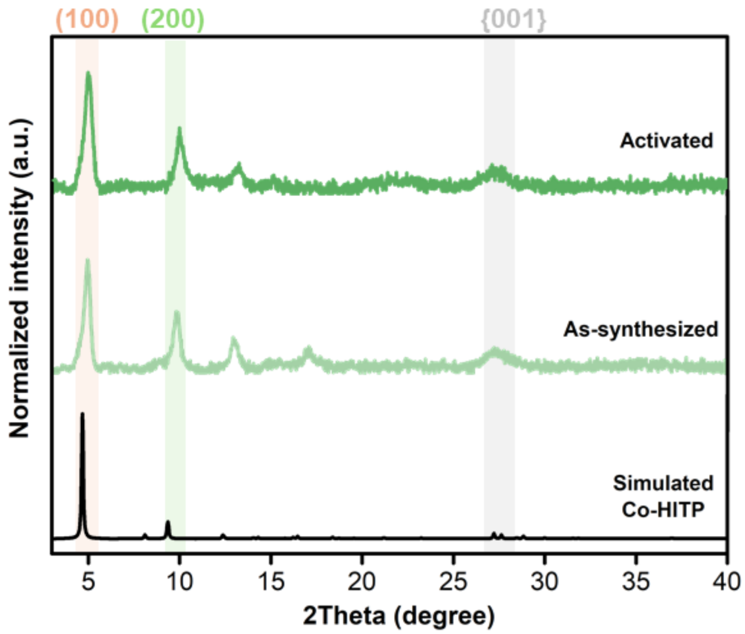

Figure S33. Powder X-ray diffraction patterns of Co-HITP before and after activation.

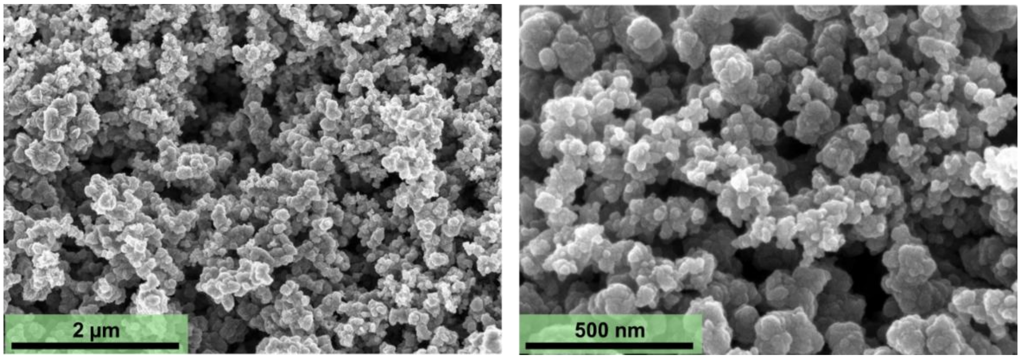

Figure S34. SEM micrographs of Co-HITP at different magnifications.

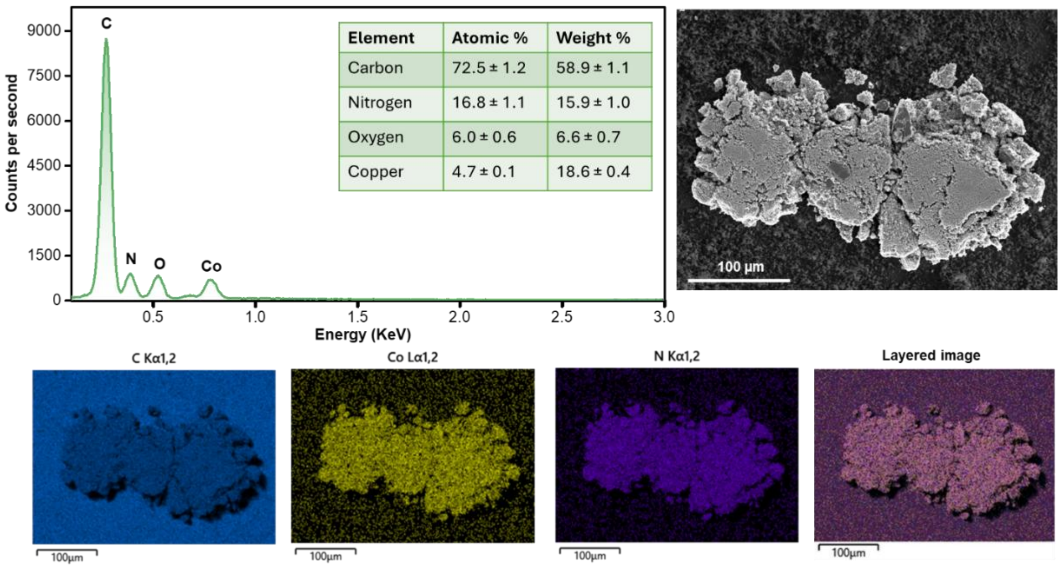

Figure S35. EDX spectrum and elemental mapping images of Co-HITP.

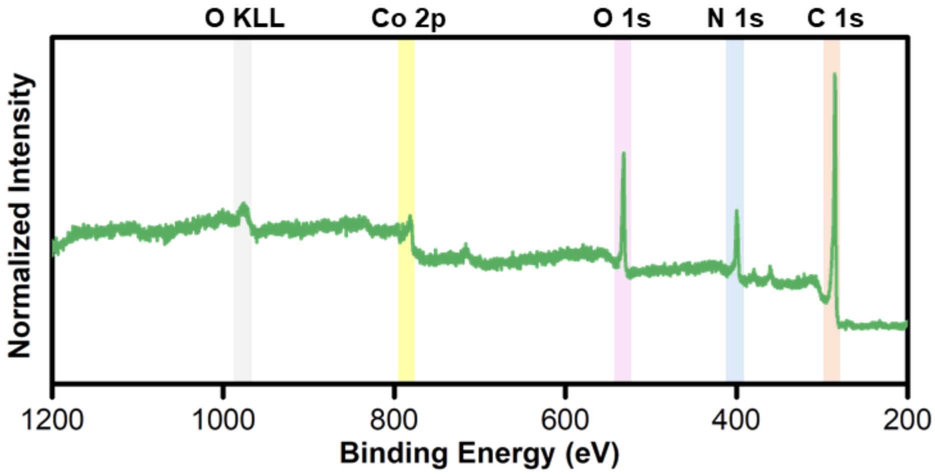

Figure S36. XPS survey spectra of Co-HITP MOF particles displaying signals related to oxygen (O 1s), nitrogen (N 1s), carbon (C 1s), and cobalt (Co 2p) elements.

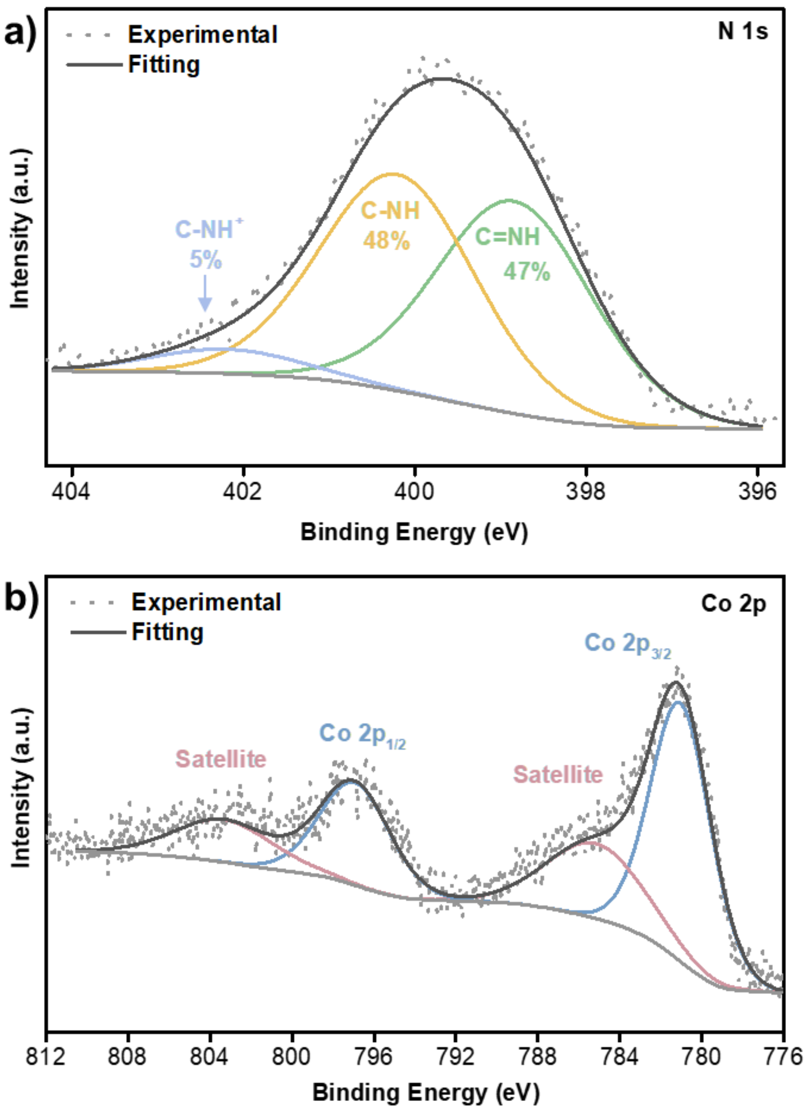

Figure S37. High-resolution XPS spectra of a) nitrogen (N 1s) and b) cobalt (Co 2p) elements for Co-HITP MOF particles.

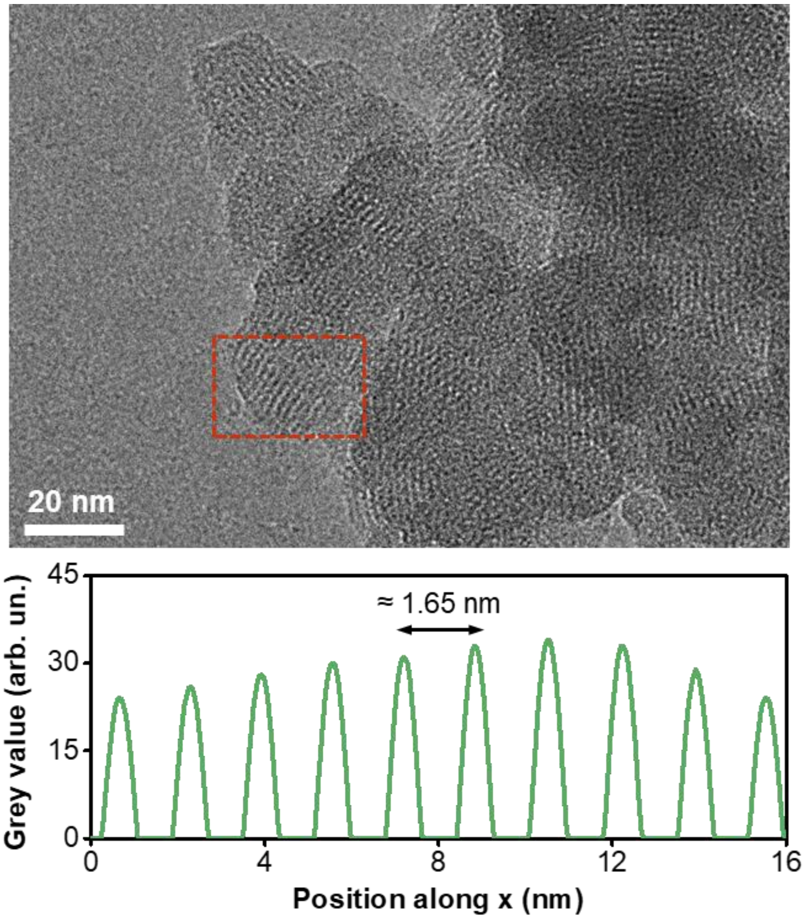

Figure S38. HR-TEM micrograph of Co-HITP along the (100) direction (top) and line intensity profile of the lattice planes (bottom). The interplanar distance is calculated to be 1.65 ± 0.04 nm.

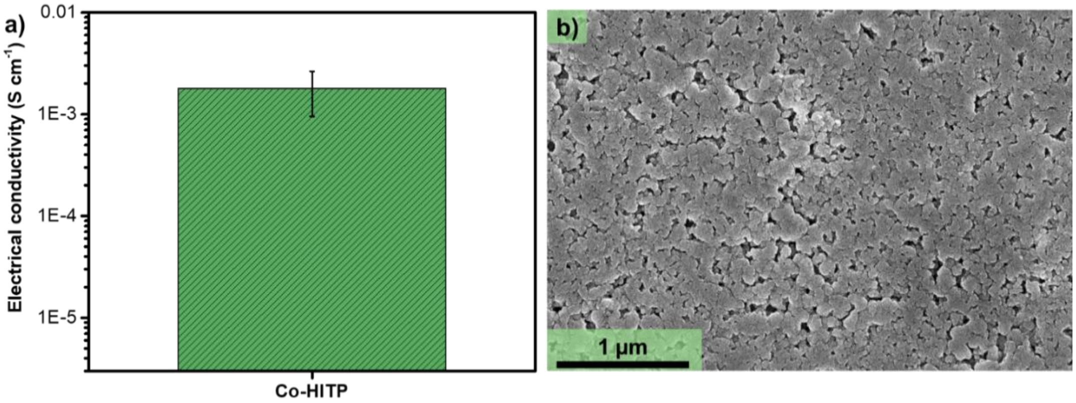

Figure S39. a) bulk electrical conductivity and b) SEM micrographs of the Co-HITP pellet following 4-point probe measurements.

4.3 Adsorption isotherms of Co-HITP

4.3.1 Adsorption isotherms of Pb^{2+} ions

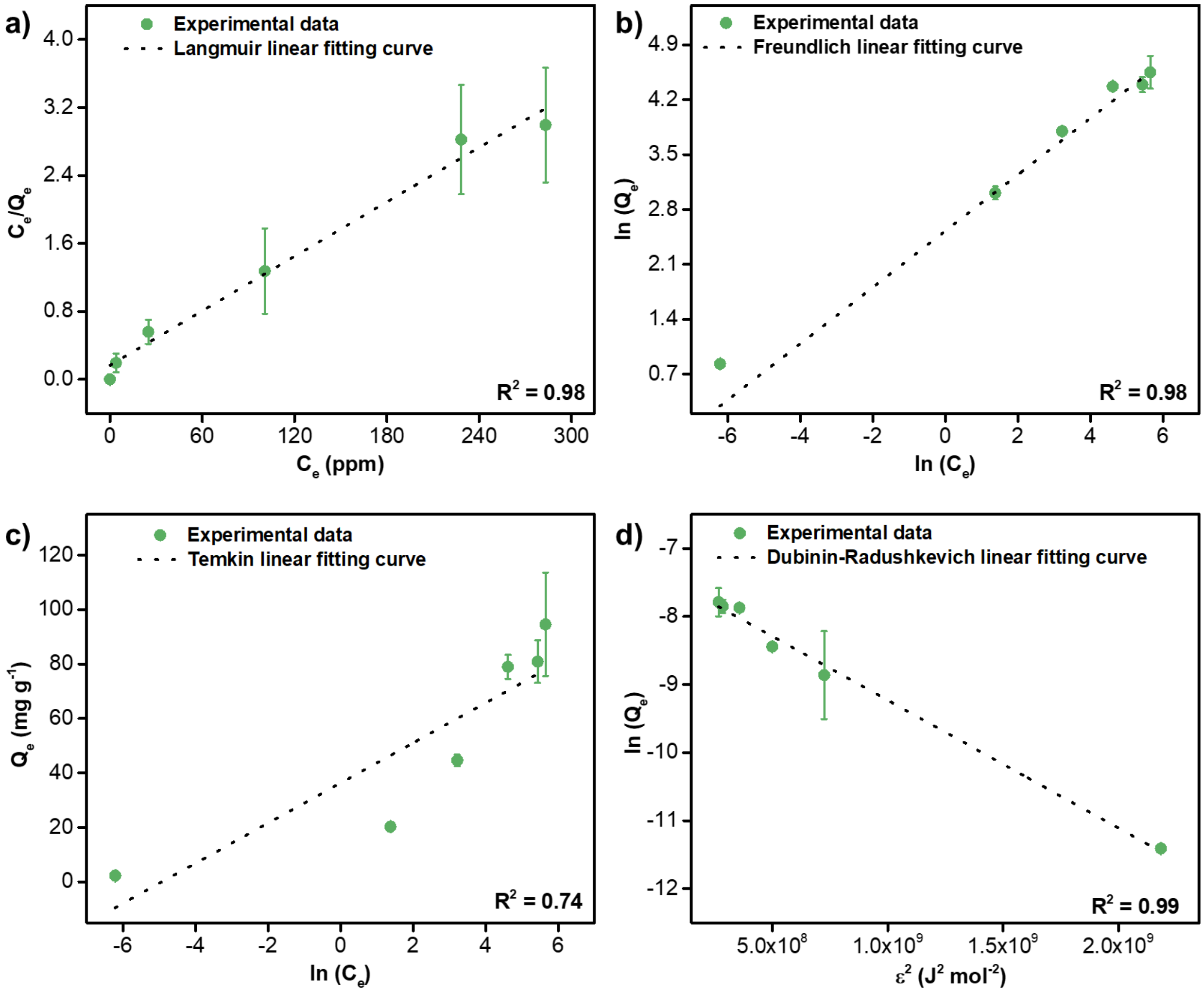

Figure S40. Experimental adsorption data fitting using a) Langmuir (**Equation S2**), b) Freundlich (**Equation S3**), c) Temkin (**Equation S4**), and d) Dubinin-Radushkevich (D-R) (**Equation S5**) linear models of Pb(II) adsorption isotherms onto Co-HITP. Conditions: m_{MOF} = 2 mg, $V_{solution}$ = 3 mL, and T = 298 K under a contact time of 4 hours. Error bars represent standard deviation from the mean value of three independent experiments.

4.3.2 Adsorption isotherms of Cd^{2+} ions

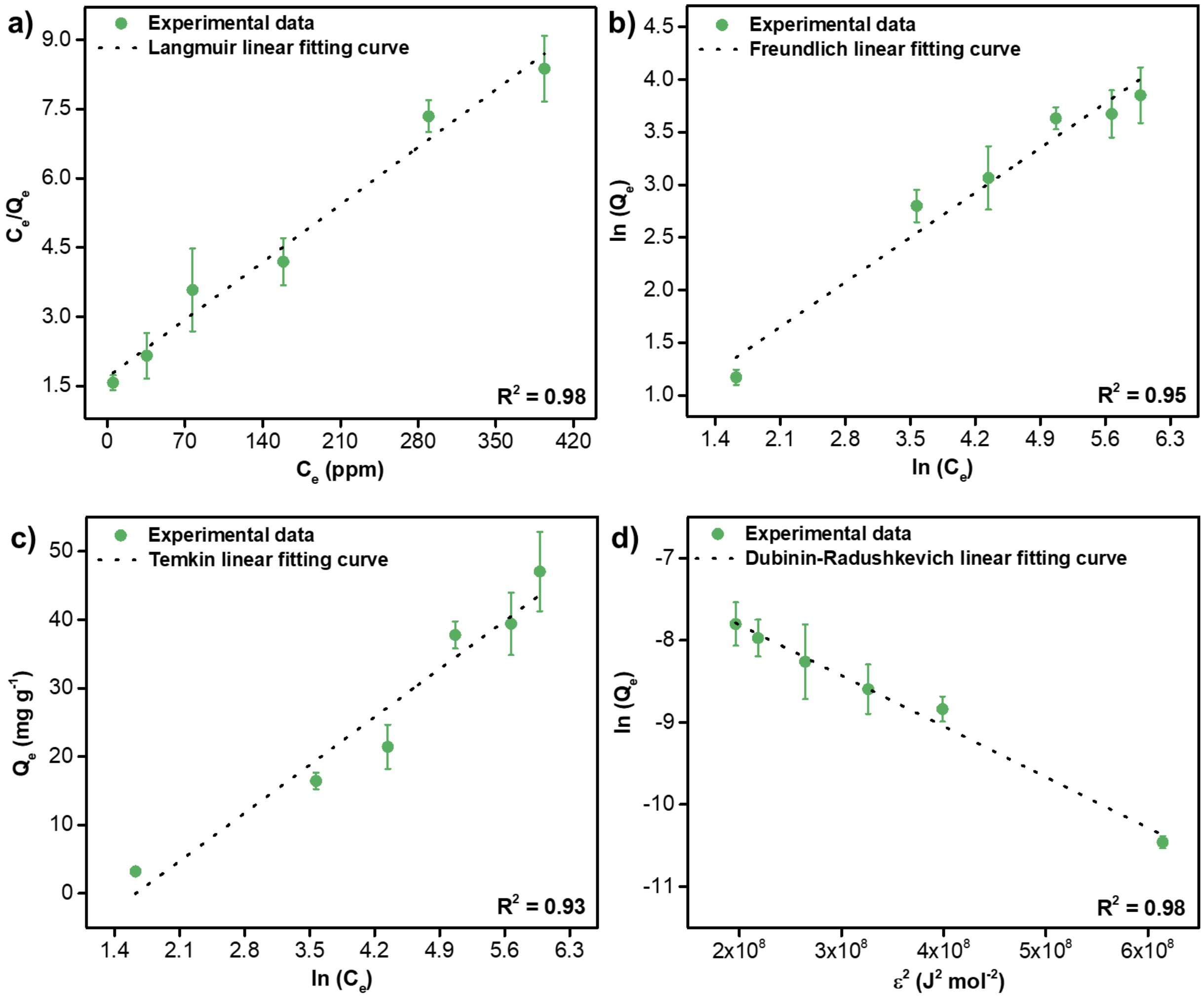

Figure S41. Experimental adsorption data fitting using a) Langmuir (**Equation S2**), b) Freundlich (**Equation S3**), c) Temkin (**Equation S4**), and d) Dubinin-Radushkevich (D-R) (**Equation S5**) linear models of Cd(II) adsorption isotherms onto Co-HITP. Conditions: m_{MOF} = 2 mg, $V_{solution}$ = 3 mL, and T = 298 K under a contact time of 4 hours. Error bars represent standard deviation from the mean value of three independent experiments.

4.3.3 Adsorption isotherms of Hg^{2+} ions

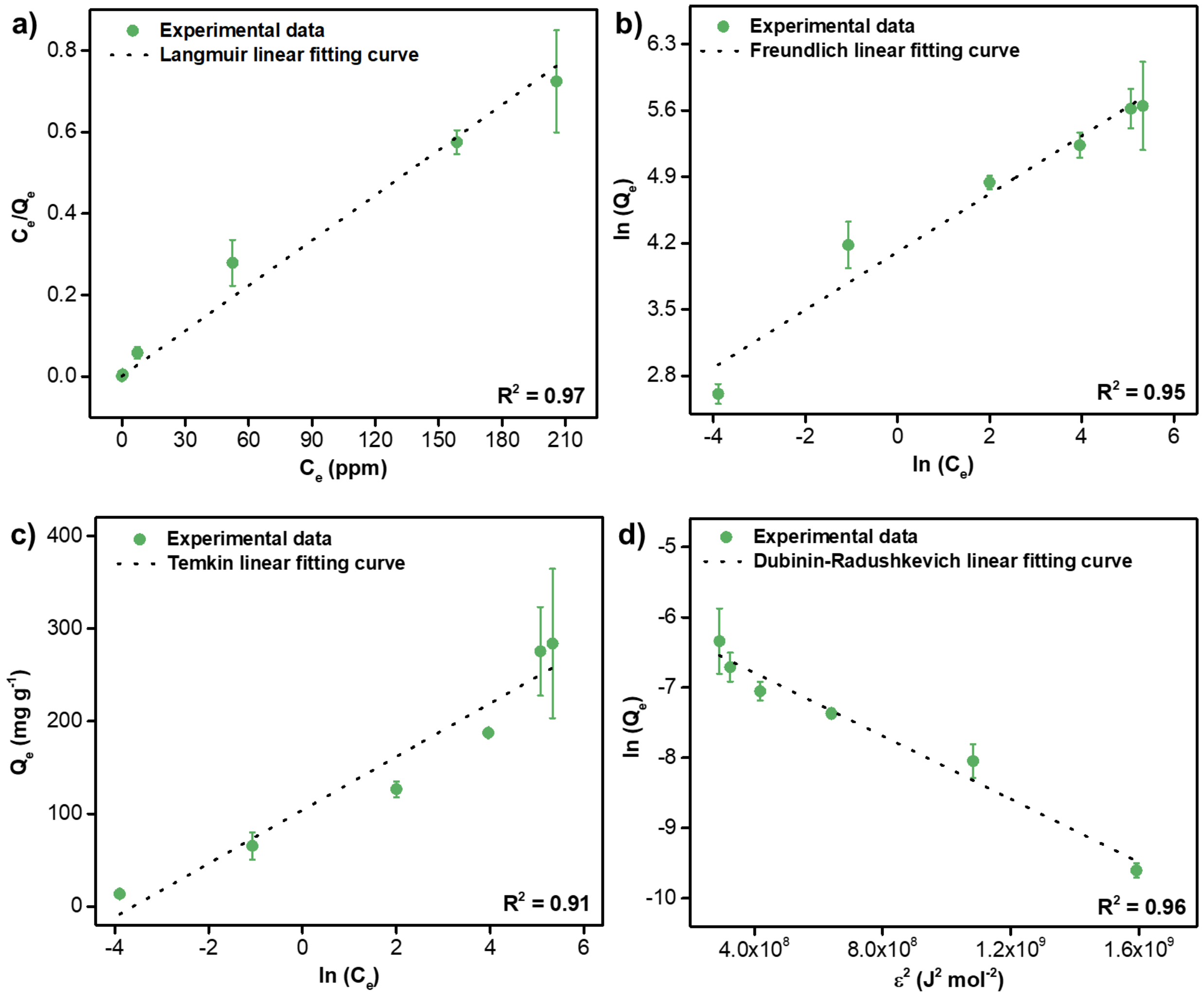

Figure S42. Experimental adsorption data fitting using a) Langmuir (**Equation S2**), b) Freundlich (**Equation S3**), c) Temkin (**Equation S4**), and d) Dubinin-Radushkevich (D-R) (**Equation S5**) linear models of Hg(II) adsorption isotherms onto Co-HITP. Conditions: m_{MOF} = 2 mg, $V_{solution}$ = 3 mL, and T = 298 K under a contact time of 4 hours. Error bars represent standard deviation from the mean value of three independent experiments.

Parameters	Co-HITP@Pb(II)	Co-HITP@Cd(II)	Co-HITP@Hg(II)
	**Langmuir Isotherm**		
**Q_{max} (mg g^{-1})**	93.5	56.2	270.3
**K_L (L/mg)**	0.1	0.01	2.5
	**Freundlich isotherm**		
**K_F (mg g^{-1})**	12.5	1.4	60.9
**n**	2.8	1.6	3.3
	**Temkin isotherm**		
**B (J mol^{-1})**	85.9	246.9	85.9
**K_T (L mg^{-1})**	36.9	0.2	36.9
	**Dubinin-Radushkevich isotherm**		
**K (mol^2 j^{-2})**	1.9 x 10^{-9}	6.2 x 10^{-9}	2.2 x 10^{-9}
**E (Kj mol^{-1})**	16.3	9.0	14.9

Table S4. Linear equilibrium modelling parameters for the adsorption of Pb(II), Cd(II), and Hg(II) onto Co-HITP MOF.

4.4 Comparison in performance with Co-HHTP

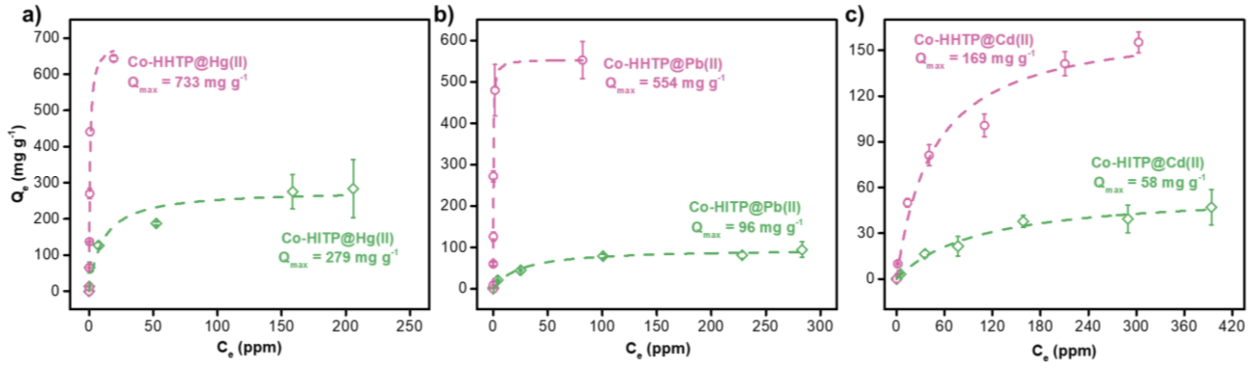

Figure S43. Non-linear Langmuir adsorption data fitting of Co-HHTP and Co-HITP towards a) Hg(II), b) Pb(II), and c) Cd(II) in water. Conditions: m_{MOF} = 2 mg, $V_{solution}$ = 3 mL, and T = 298 K under a contact time of 4 hours. Error bars represent standard deviation from the mean value of three independent experiments

In addition to differences in stacking arrangements between Co-HHTP and Co-HITP, several other factors must be considered to fairly compare their adsorption performance. Key parameters include i) BET surface area, ii) metal-ligand coordination environment (–O in Co-HHTP versus –NH in Co-HITP), and iii) particle size and aspect ratio. Numerous studies on MOFs have highlighted the influence of these factors on the adsorption of heavy-metal ions such as Hg^{2+}, Pb^{2+}, and Cd^{2+}, demonstrating clear structure–performance relationships. First, higher BET surface area facilitates greater diffusion of metal ions into the pores of MOFs, thereby increasing adsorption capacity.[7, 8] Second, amine functional groups typically act as strong electron donors, forming robust charge-transfer complexes with heavy-metal cations, as demonstrated in our previous work,[9] and others.[10, 11] Third, smaller MOF particles and lower length-to-width aspect ratios typically expose more edge sites and shorten diffusion paths for ions, leading to a higher density of accessible adsorption sites.[12] Given that Co-HITP exhibits a i) higher BET surface area, ii) Co–NH coordination environment, and iii) significantly smaller particle size (10-20 nm) and length-to-width aspect ratio (~1), it would theoretically be expected to show superior adsorption capacity. However, Co-HITP demonstrates up to five-fold lower adsorption capacity compared to Co-HHTP, emphasizing the critical role of intercalated layers in enhancing adsorption performance in Co-HHTP.

5. Surface charge properties of MOFs

5.1 Dye uptake experiments

To determine the surface charge properties of both HHTP- and HITP-based MOFs employed in this study, we analyzed their dye adsorption behavior by exposing the MOF materials to cationic and anionic dyes. In brief, we exposed 2 mg of activated MOF powders to 6 mL solutions methyl orange dye (25 ppm, anionic dye) and methylene blue dye (25 and 100 ppm, cationic dye) for 24

hours. The MOFs were then filtered using nonsterile 0.45 μm nylon syringe filters and the remaining dye solutions were subjected to UV-Vis spectrophotometry analysis.

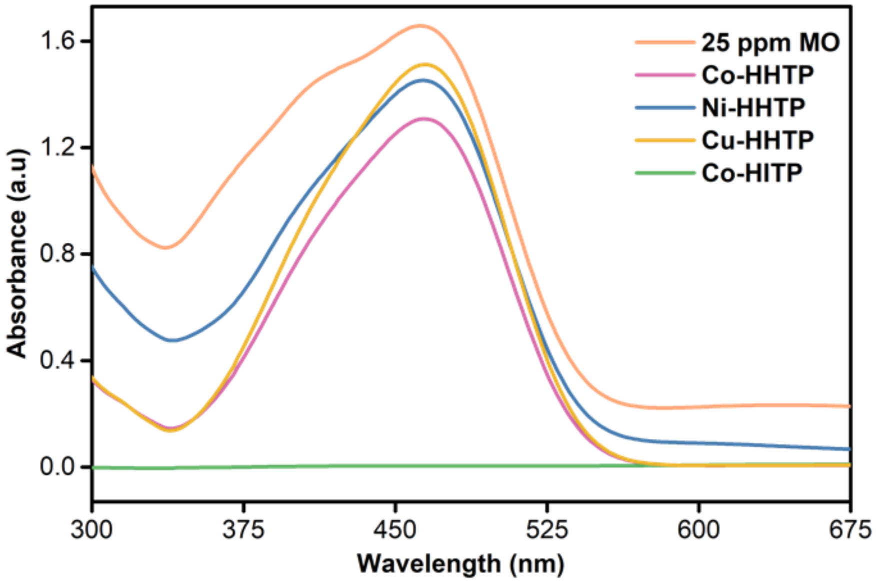

Figure S44. Methyl orange dye adsorption onto HHTP- and HITP-MOFs. Conditions: [MO] = 25 ppm, m_{MOF} = 2 mg, $V_{solution}$ = 6 mL, T = 298 K, and stirring time = 24 hours.

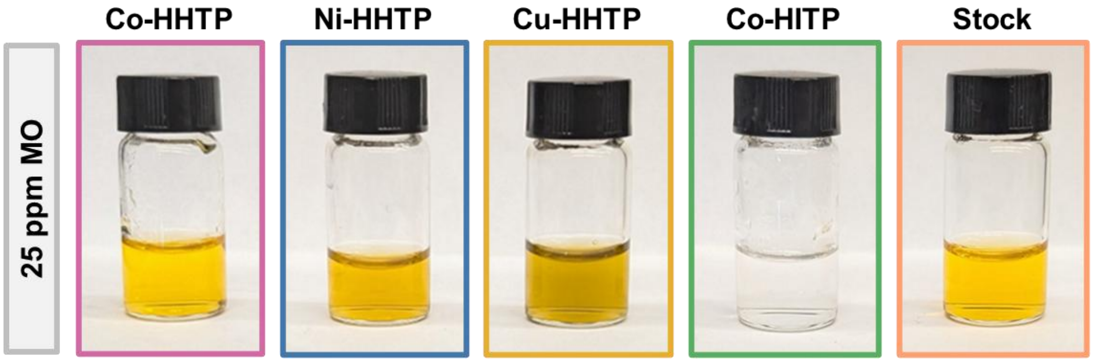

Figure S45. Photographs of the resulting MO dye solutions following adsorption (25 ppm, 24 hrs).

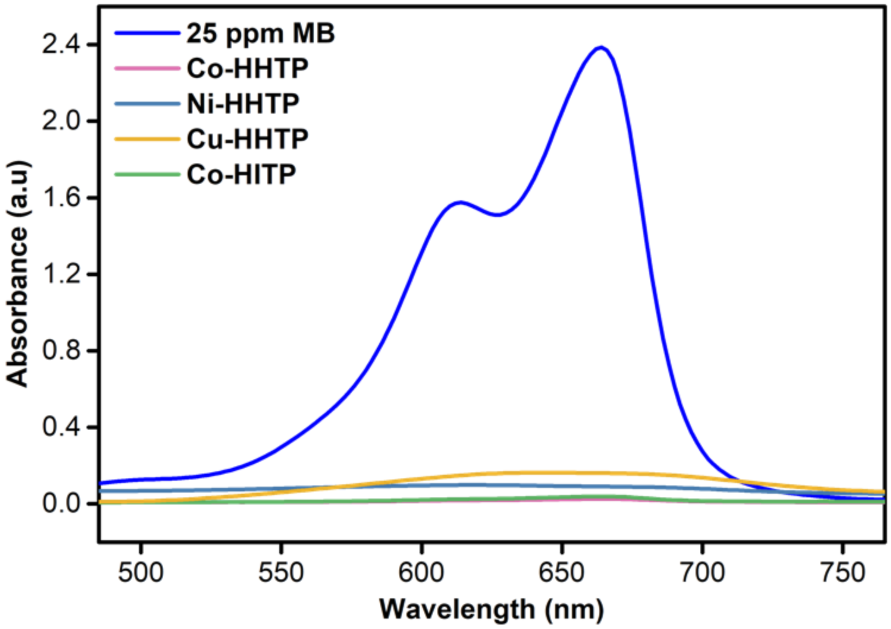

Figure S46. Methylene blue dye adsorption onto HHTP- and HITP-MOFs. Conditions: [MB] = 25 ppm, m_{MOF} = 2 mg, $V_{solution}$ = 6 mL, T = 298 K, and stirring time = 24 hours.

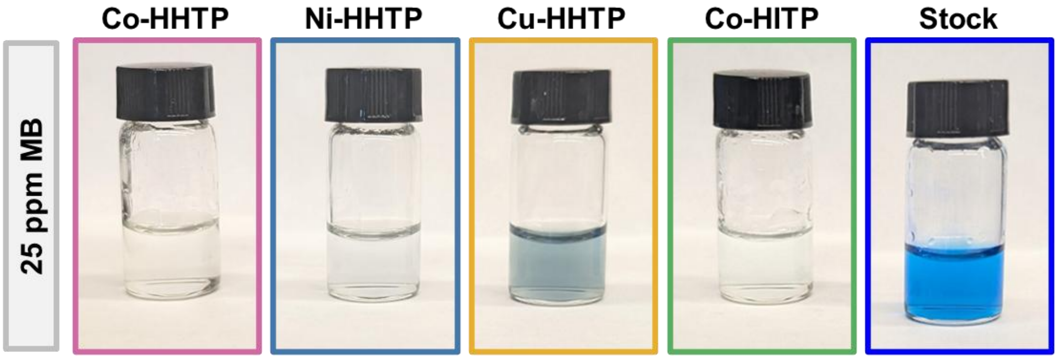

Figure S47. Photographs of the resulting MB dye solutions following adsorption (25 and 100 ppm, 24 hours).

5.2 Zeta potential measurements

Zeta potential measurements were carried out on a Malvern ZetaSizer Nano. In brief, 1 mg of MOF powders were homogeneously dispersed in 1 mL of Milli-Q water by sonication overnight (12 hours). The resulting suspension was transferred into a Malvern panalytical folded capillary zeta cell and the Zeta potential values were recorded at a temperature of 25 °C.

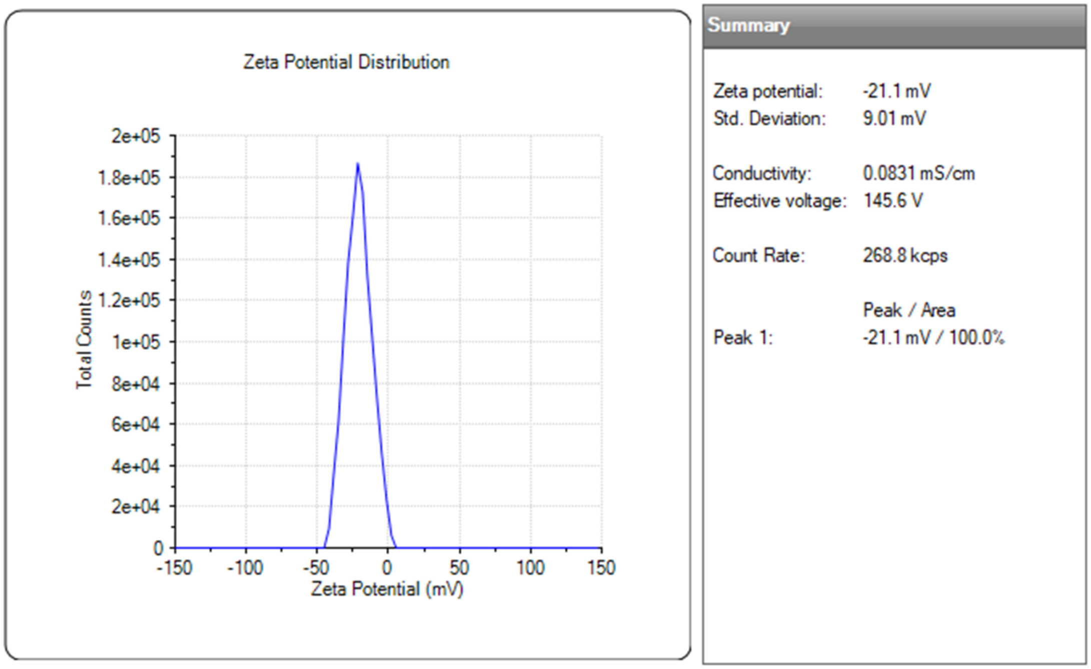

Figure S48. Zeta potential measurement of Co-HHTP in water (1 mg mL^{-1}).

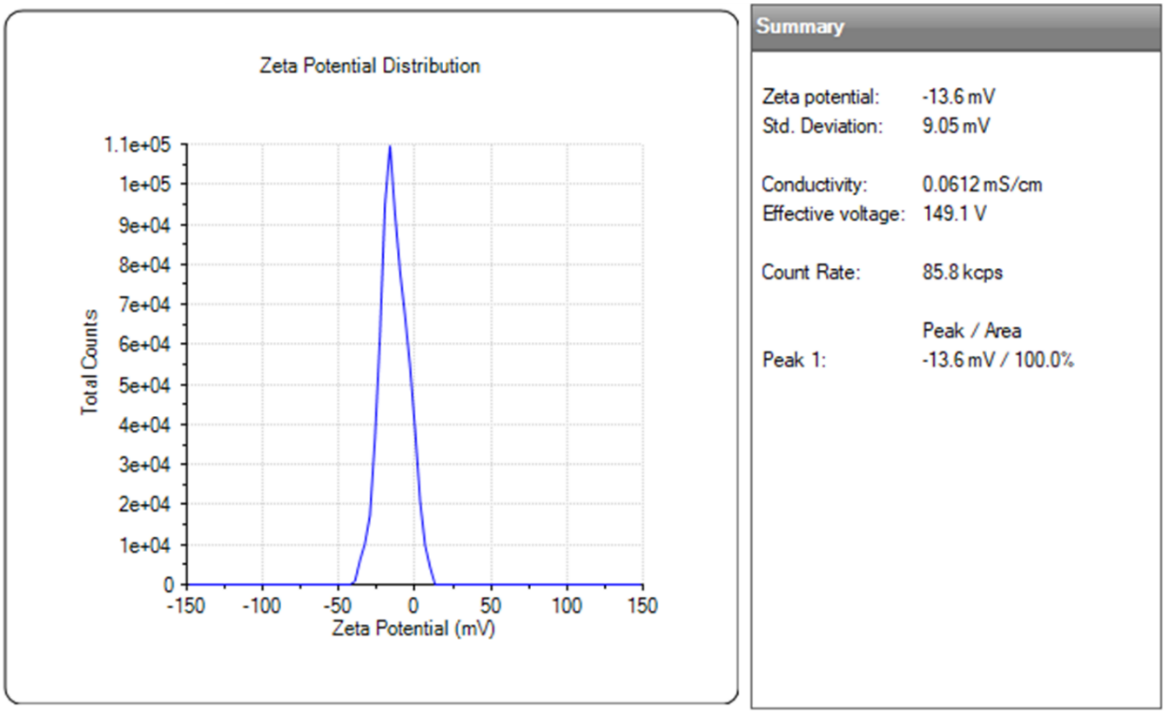

Figure S49. Zeta potential measurement of Ni-HHTP in water (1 mg mL^{-1}).

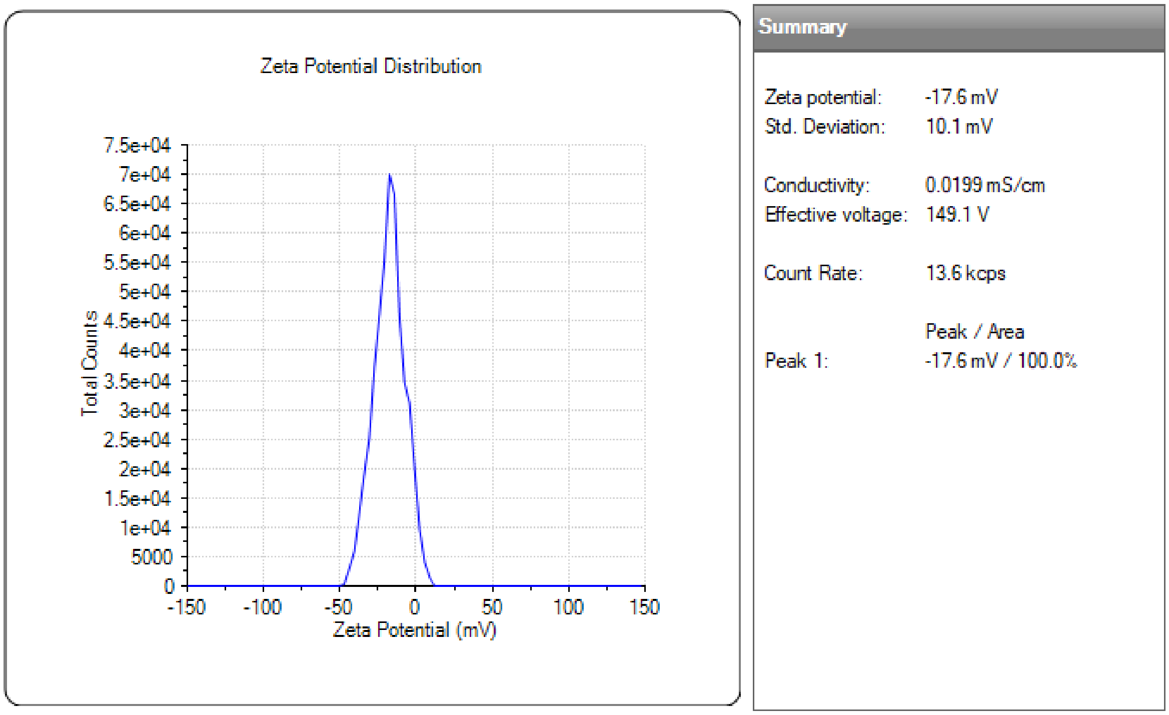

Figure S50. Zeta potential measurement of Cu-HHTP in water (1 mg mL^{-1}).

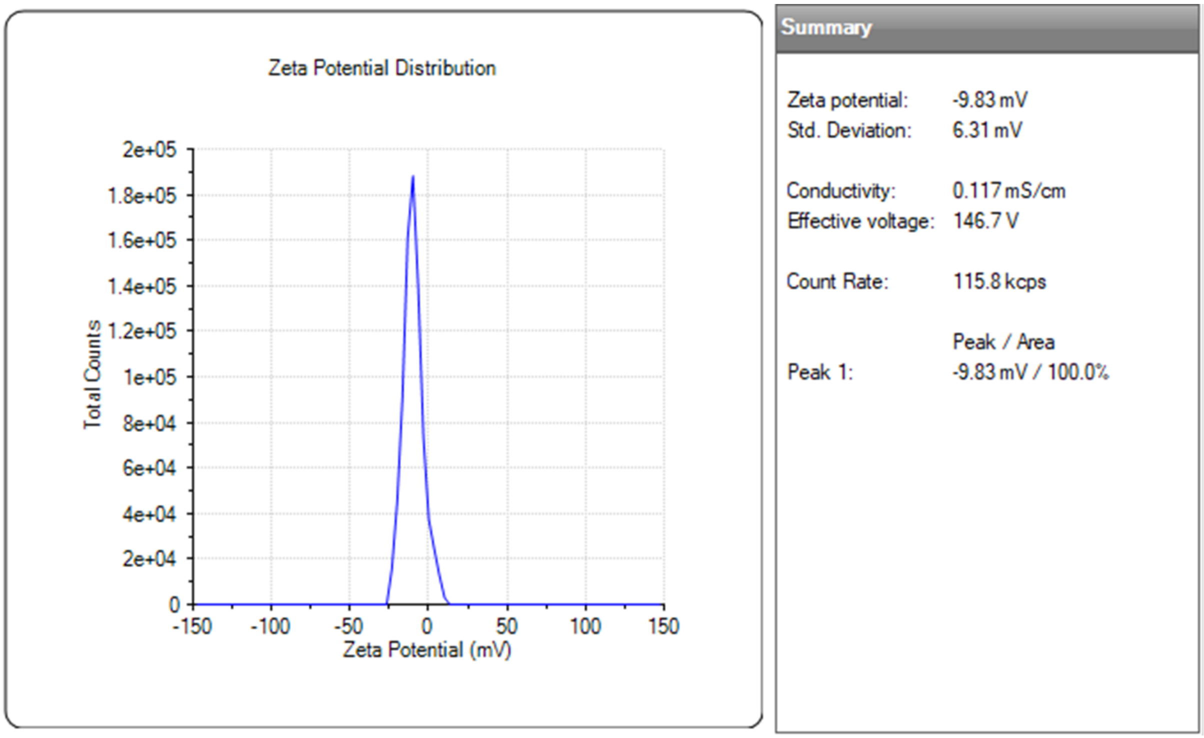

Figure S51. Zeta potential measurement of Co-HITP in water (1 mg mL^{-1}).

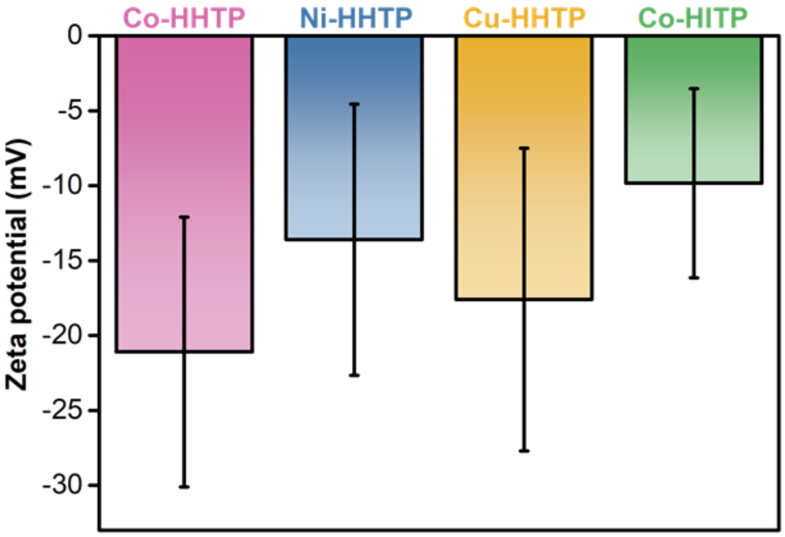

Figure S52. Comparison of Zeta potential values of the HHTP- and HITP-based MOFs in water (1 mg mL^{-1}) used in this study

Although zeta potential measurements confirmed a negatively charged surface for all HHTP-based MOFs, the interaction between the MOF surface and heavy-metal cations is not governed solely by electrostatics. A notable trend emerges when comparing Co-HHTP and Ni-HHTP, both of which possess the same stacking arrangement. The higher adsorption capacity and fast kinetics observed for Co-HHTP can be correlated with its more negative surface charge (-21.1 mV vs -13.6 mV for Ni-HHTP), which enhances electrostatic attraction toward the heavy metal cationic species and facilitates their diffusion into the framework. This suggests that surface charge can modulate the adsorption rate and extent in systems where other structural factors remain constant. However, this correlation does not apply when comparing Co-HHTP to Cu-HHTP or Co-HITP, as differences in stacking arrangements and interlayer intercalation dominate the adsorption process. Therefore, while zeta potential provides mechanistic insight within structurally comparable systems, it cannot be considered a universal descriptor across MOFs with different topologies and coordination environments.

6. Time-dependent adsorption studies

The uptake capacity Q_t, representing the amount of contaminants (adsorbate) adsorbed by one gram of the MOF material ($mg_{contaminant}/g_{MOF}$) at a specific time interval *t* was determined using **Equation S8** as follows:

$$Q_t(mg\ g^{-1}) = \frac{C_0 - C_e}{m} \times V \qquad \textbf{(Equation S8)}$$

Where C_0 is the initial concentration of contaminant (in ppm), C_e is the concentration of contaminant remaining in solution after adsorption at a specific time *t* (in ppm), *V* is the volume of the solution in which the pollutant is dissolved in (in mL), and *m* is the mass of the MOF sample used for adsorption (in mg). In order to predict the mechanism of the adsorption process of Pb(II), Cd(II), and Hg(II) onto the MOF materials, the experimental data were fitted with different kinetic models that are indicated below.

6.1 Adsorption isotherm models

6.1.1 Pseudo-first order kinetic model

The pseudo-first order kinetic model assumes the rate of ion adsorption to be directly proportional to the number of active adsorptive sites on the MOF adsorbent surface. As such, the estimated change in the contaminants' concentrations over time follows a first-order rate law. A good fit of the experimental adsorption data indicates a dominant physical adsorption process between the contaminants and the MOF materials, involving non-covalent interactions such as Van der Waals, hydrogen bonds, and π- π interactions.

We fitted the experimental adsorption data to the linear pseudo-first order kinetic model according to **Equation S9**.

$$\log(Q_e - Q_t) = \log Q_e - (\frac{K_1}{2.303})t \qquad \textbf{(Equation S9)}$$

Where Q_e is the uptake capacity at a specific concentration (in mg g^{-1}), Q_t is the uptake capacity at a specific time *t* (in mg g^{-1}), K_1 is the pseudo-first order rate constant (in min^{-1}), and *t* is the duration of contact between the MOF and the contaminants (in min).

6.1.2 Pseudo-second order kinetic model

The pseudo-second order kinetic model assumes the ion adsorption rate to be proportional to the square of the number of active adsorptive sites on the MOF homogeneous surface. As such, the model suggests a bimolecular adsorption process, whereby the rate-determining step depends on both the MOF nanocrystals and the contaminants concentrations. A good fit of the experimental adsorption data indicates a dominant chemisorption process involving the formation of chemical/covalent bonds between the adsorbents and adsorbate.

We fitted the experimental adsorption data to the linear pseudo-second order kinetic model according to **Equation S10**.

$$\frac{t}{Q_t} = \frac{t}{Q_e} + \frac{1}{K_2 Q_e^2} \qquad \textbf{(Equation S10)}$$

Where K_2 is the pseudo-second-order rate constant (g mg^{-1} min^{-1}).

6.1.3 Elovich kinetic model

Similar to the pseudo-second order kinetic model, Elovich kinetic is widely used to describe chemisorption processes. Nonetheless, it assumes the MOF surface to be energetically heterogeneous, having distinct types of adsorptive active sites. As such, the adsorption sites on the MOF surface vary in both, accessibility and affinity for contaminants.[13]

We fitted the experimental adsorption data to the linear Elovich kinetic model according to **Equation S11**.

$$Q_t = \frac{1}{\beta}\ln(\alpha\beta) + \frac{1}{\beta}\mathrm{t} \qquad \textbf{(Equation S11)}$$

Where α is the initial adsorption rate (in mg g^{-1} min^{-1}) and β is the desorption constant (in g mg^{-1}), which is related to the extent of surface coverage and the activation energy for adsorption.

6.2 Kinetic isotherms of Pb^{2+} ions

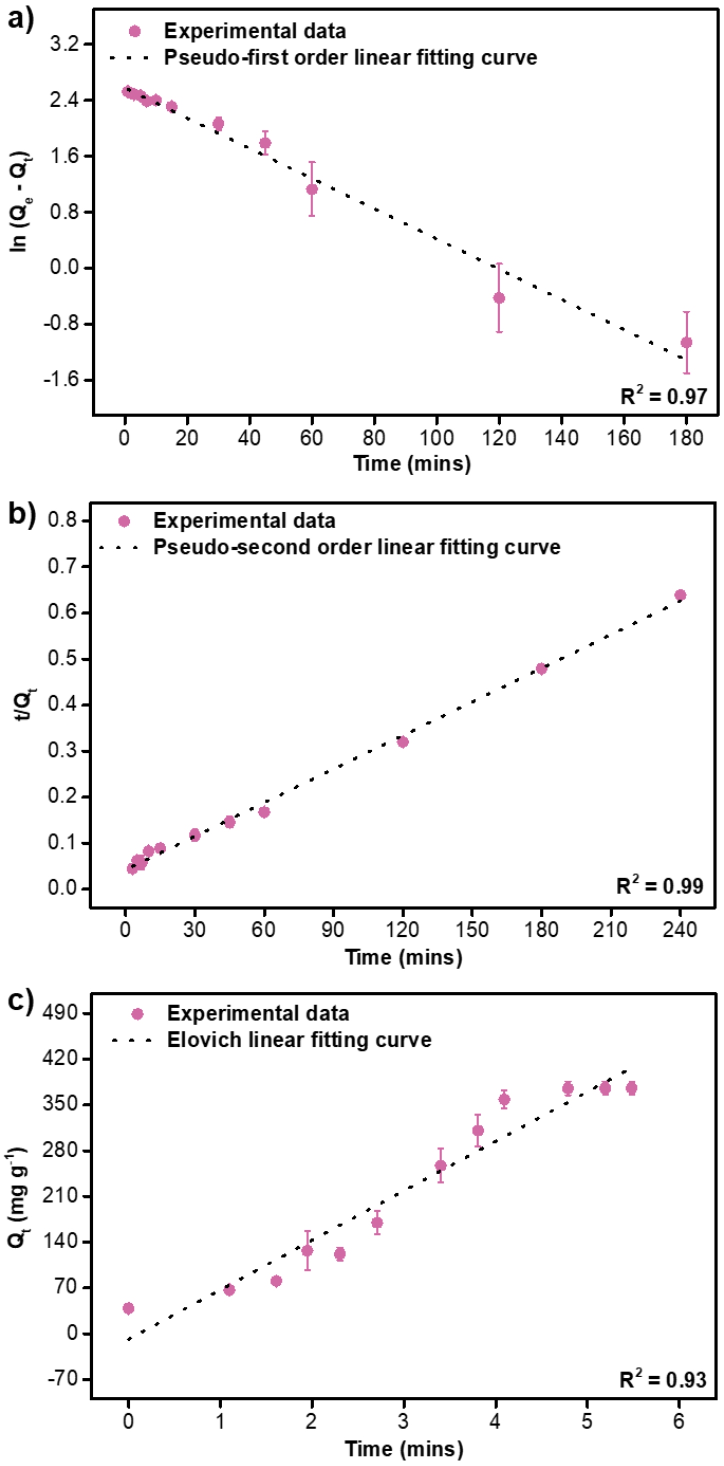

Figure S53. Experimental adsorption data fitting using a) pseudo-first order (**Equation S9**), b) pseudo-second order (**Equation S10**), and c) Elovich (**Equation S11**) linear models of Pb(II) adsorption isotherms onto Co-HHTP. Conditions: m_{MOF} = 2 mg, $V_{solution}$ = 3 mL, and T = 298 K at different time intervals. Error bars represent standard deviation from the mean value of three independent experiments.

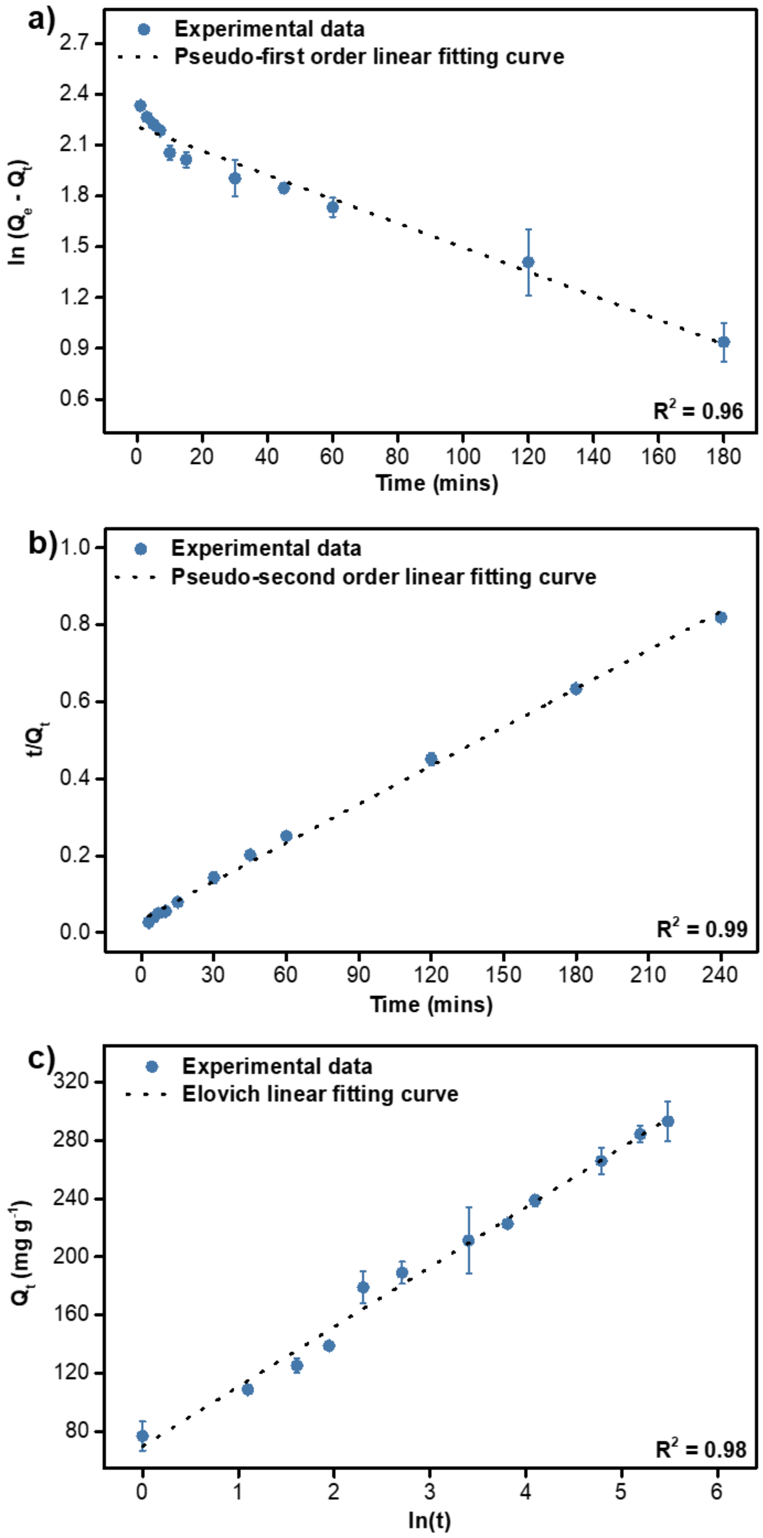

Figure S54. Experimental adsorption data fitting using a) pseudo-first order (**Equation S9**), b) pseudo-second order (**Equation S10**), and c) Elovich (**Equation S11**) linear models of Pb(II) adsorption isotherms onto Ni-HHTP. Conditions: m_{MOF} = 2 mg, $V_{solution}$ = 3 mL, and T = 298 K at different time intervals. Error bars represent standard deviation from the mean value of three independent experiments.

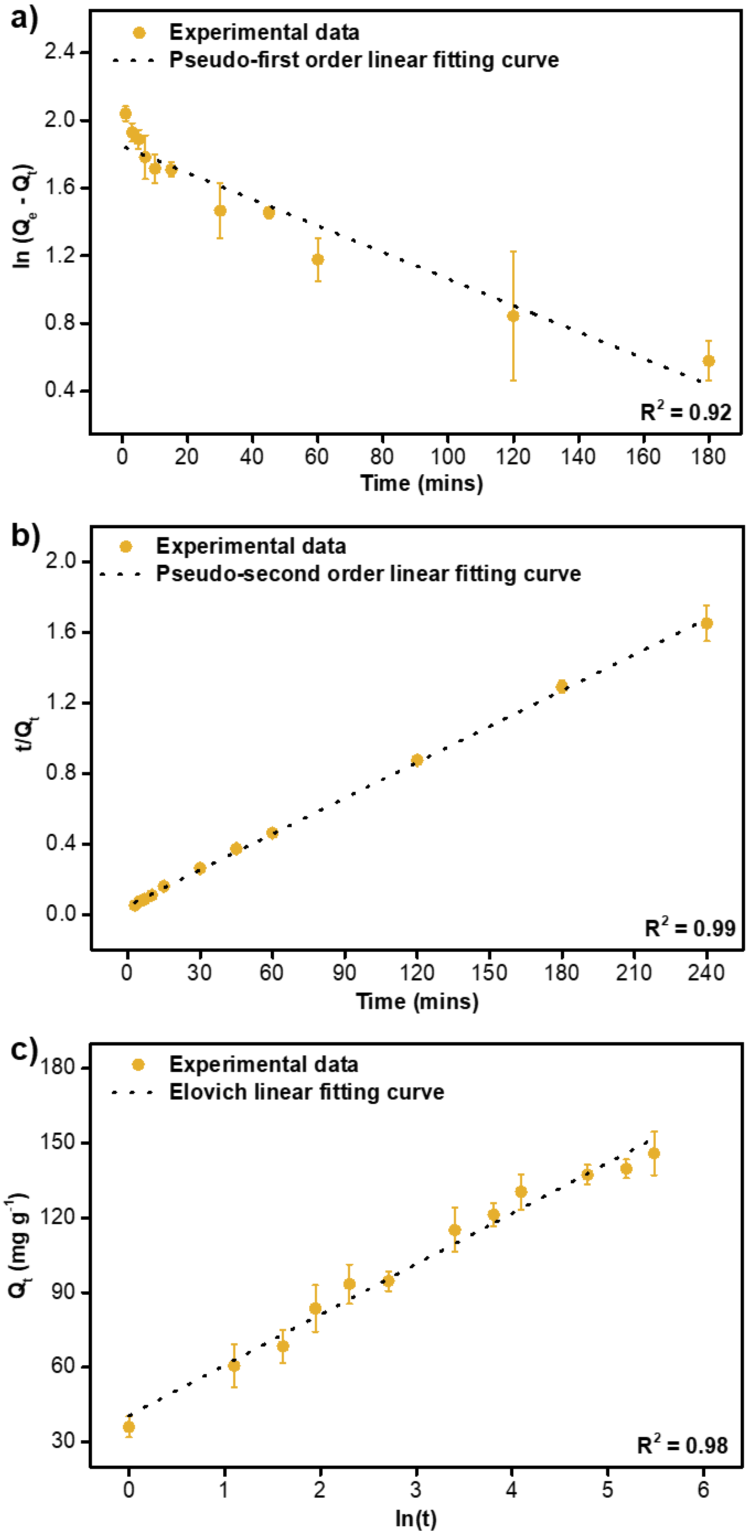

Figure S55. Experimental adsorption data fitting using a) pseudo-first order (**Equation S9**), b) pseudo-second order (**Equation S10**), and c) Elovich (**Equation S11**) linear models of Pb(II) adsorption isotherms onto Cu-HHTP. Conditions: m_{MOF} = 2 mg, $V_{solution}$ = 3 mL, and T = 298 K at different time intervals. Error bars represent standard deviation from the mean value of three independent experiments.

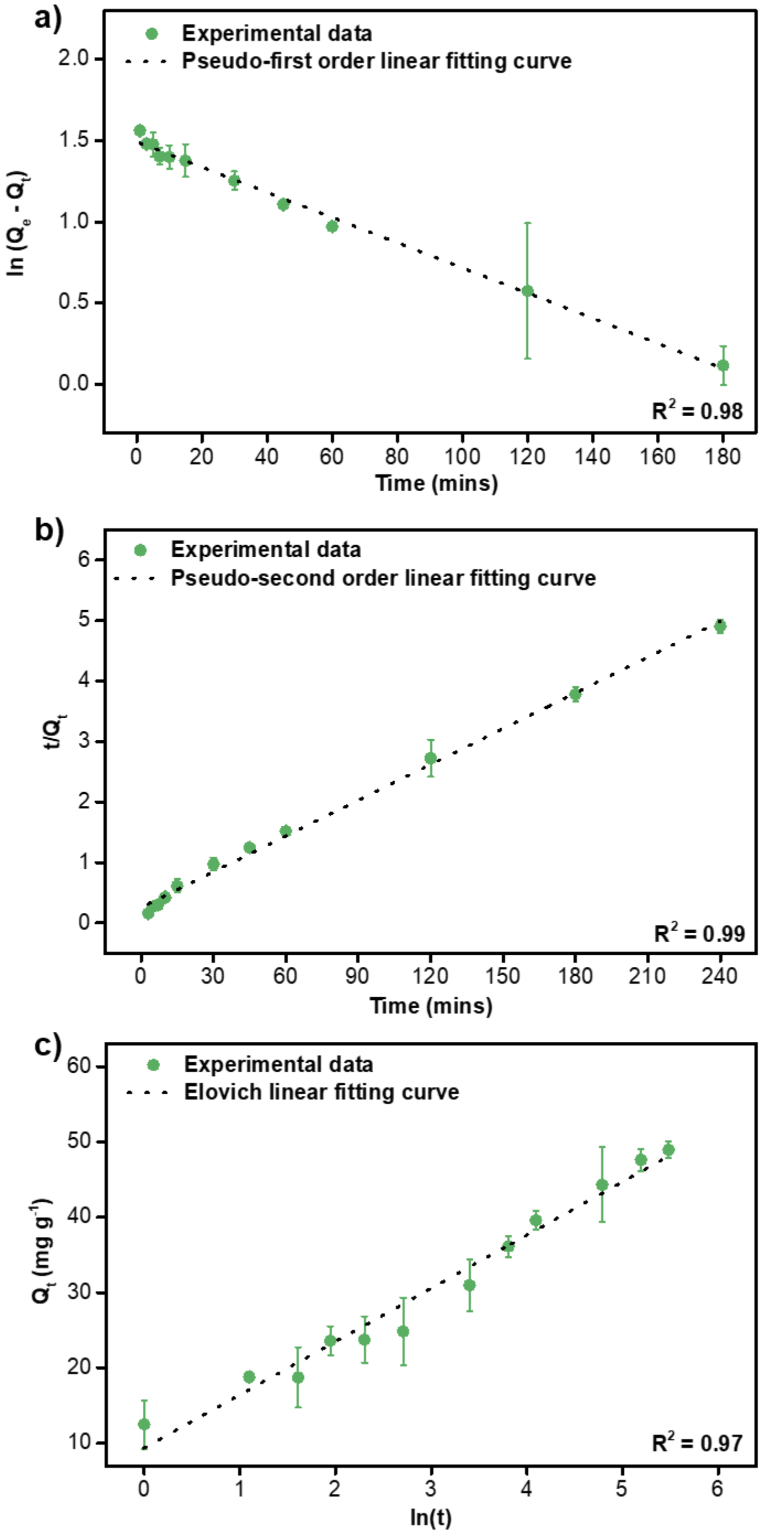

Figure S56. Experimental adsorption data fitting using a) pseudo-first order (**Equation S9**), b) pseudo-second order (**Equation S10**), and c) Elovich (**Equation S11**) linear models of Pb(II) adsorption isotherms onto Co-HITP. Conditions: m_{MOF} = 2 mg, $V_{solution}$ = 3 mL, and T = 298 K at different time intervals. Error bars represent standard deviation from the mean value of three independent experiments.

Parameters	Co-HHTP	Ni-HHTP	Cu-HHTP	Co-HITP
**Pseudo-first order kinetic isotherm**				
**K_1 (min^{-1})**	0.050	0.016	0.018	0.018
**Pseudo-second order kinetic isotherm**				
**K_2 ($g\ mg^{-1}\ min^{-1}$)**	1.4×10^{-4}	3.4×10^{-4}	9.5×10^{-4}	1.5×10^{-3}
**Elovich kinetic isotherm**				
**α ($J\ mol^{-1}$)**	68	23×10^{1}	15×10^{1}	27
**β ($L\ mg^{-1}$)**	0.013	0.024	0.049	0.14

Table S5. Linear kinetic modelling parameters for the adsorption of Pb(II) onto Co-HITP, Ni-HHTP, Cu-HHTP, and Co-HITP MOFs.

6.3 Kinetic isotherms of Cd^{2+} ions

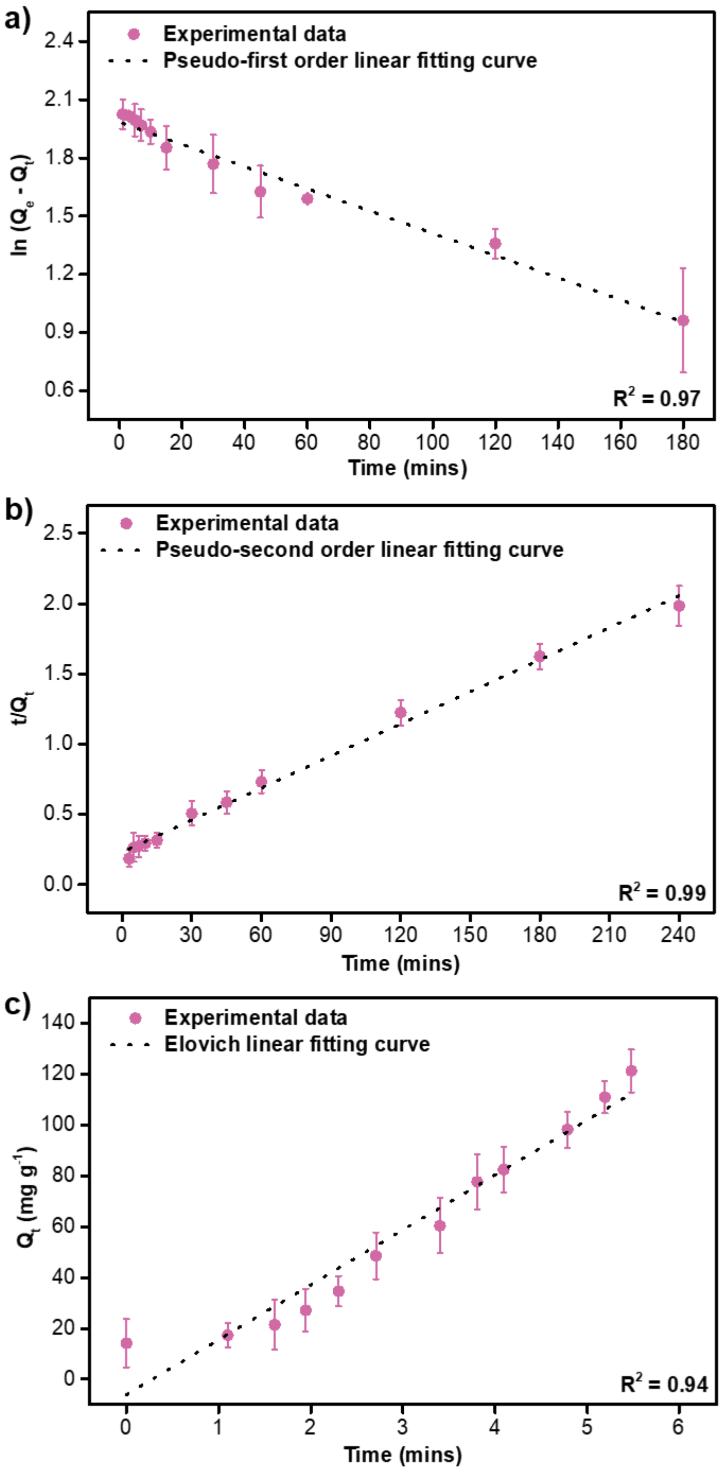

Figure S57. Experimental adsorption data fitting using a) pseudo-first order (**Equation S9**), b) pseudo-second order (**Equation S10**), and c) Elovich (**Equation S11**) linear models of Cd(II) adsorption isotherms onto Co-HHTP. Conditions: m_{MOF} = 2 mg, $V_{solution}$ = 3 mL, and T = 298 K at different time intervals. Error bars represent standard deviation from the mean value of three independent experiments.

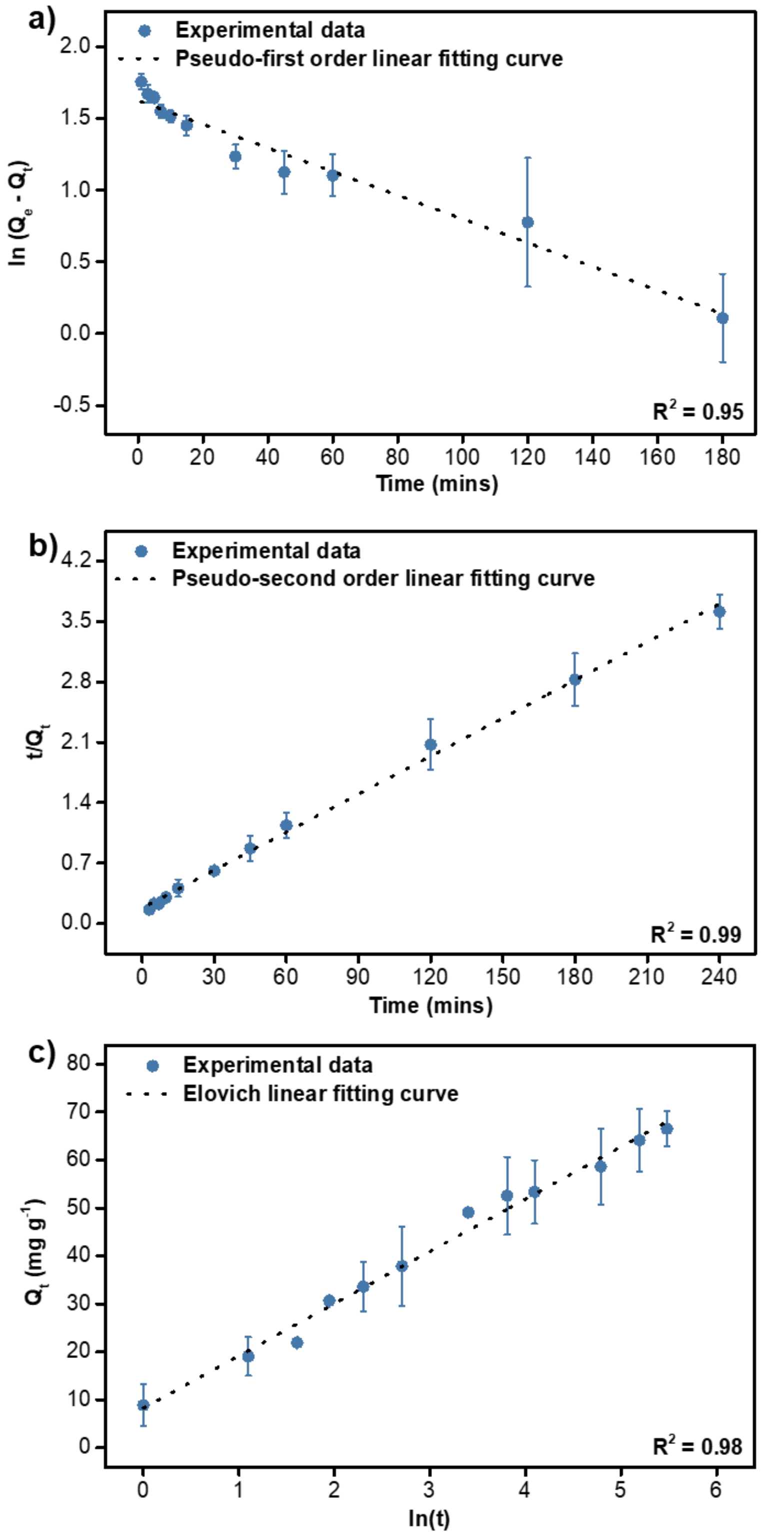

Figure S58. Experimental adsorption data fitting using a) pseudo-first order (**Equation S9**), b) pseudo-second order (**Equation S10**), and c) Elovich (**Equation S11**) linear models of Cd(II) adsorption isotherms onto Ni-HHTP. Conditions: m_{MOF} = 2 mg, $V_{solution}$ = 3 mL, and T = 298 K at different time intervals. Error bars represent standard deviation from the mean value of three independent experiments.

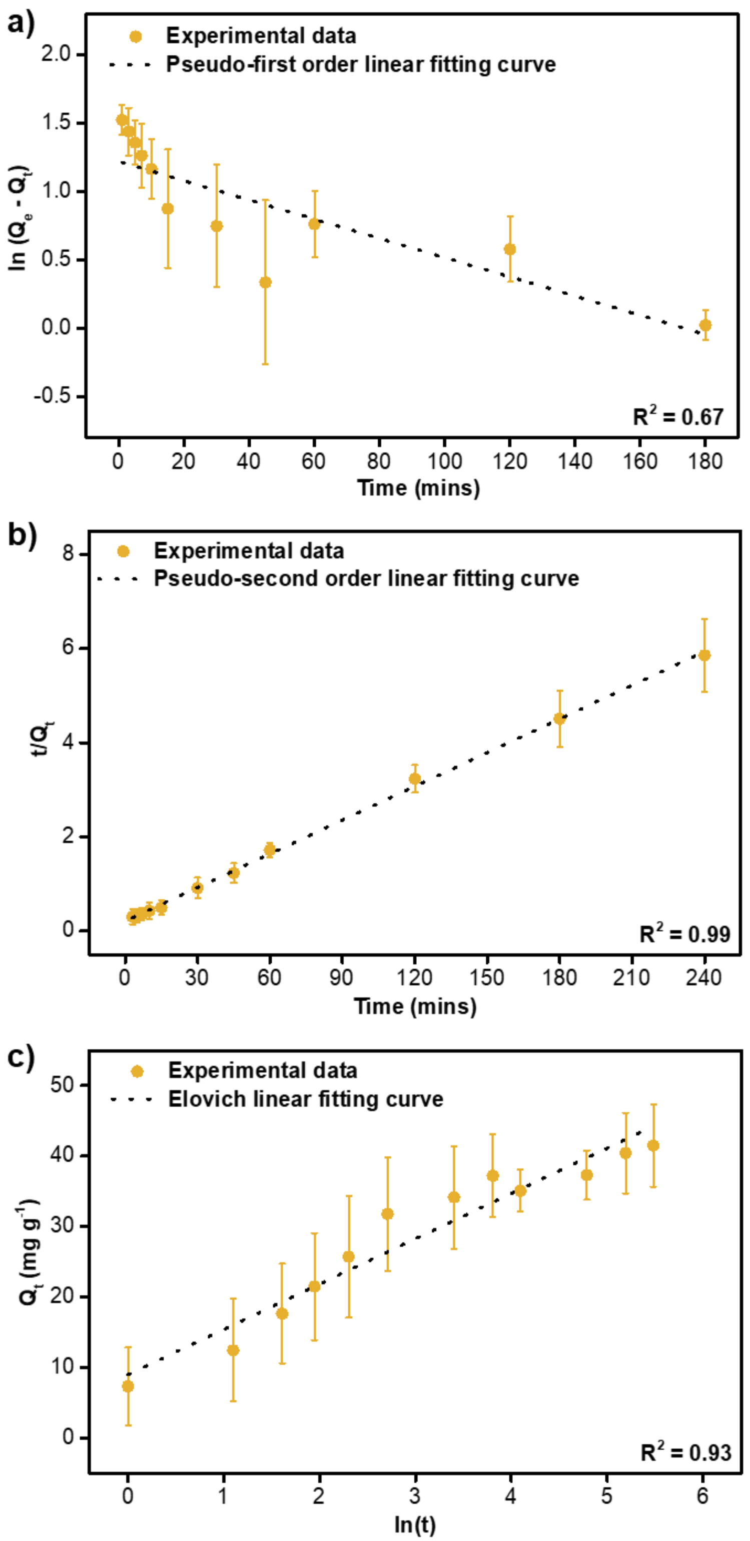

Figure S59. Experimental adsorption data fitting using a) pseudo-first order (**Equation S9**), b) pseudo-second order (**Equation S10**), and c) Elovich (**Equation S11**) linear models of Cd(II) adsorption isotherms onto Cu-HHTP. Conditions: m_{MOF} = 2 mg, $V_{solution}$ = 3 mL, and T = 298 K at different time intervals. Error bars represent standard deviation from the mean value of three independent experiments.

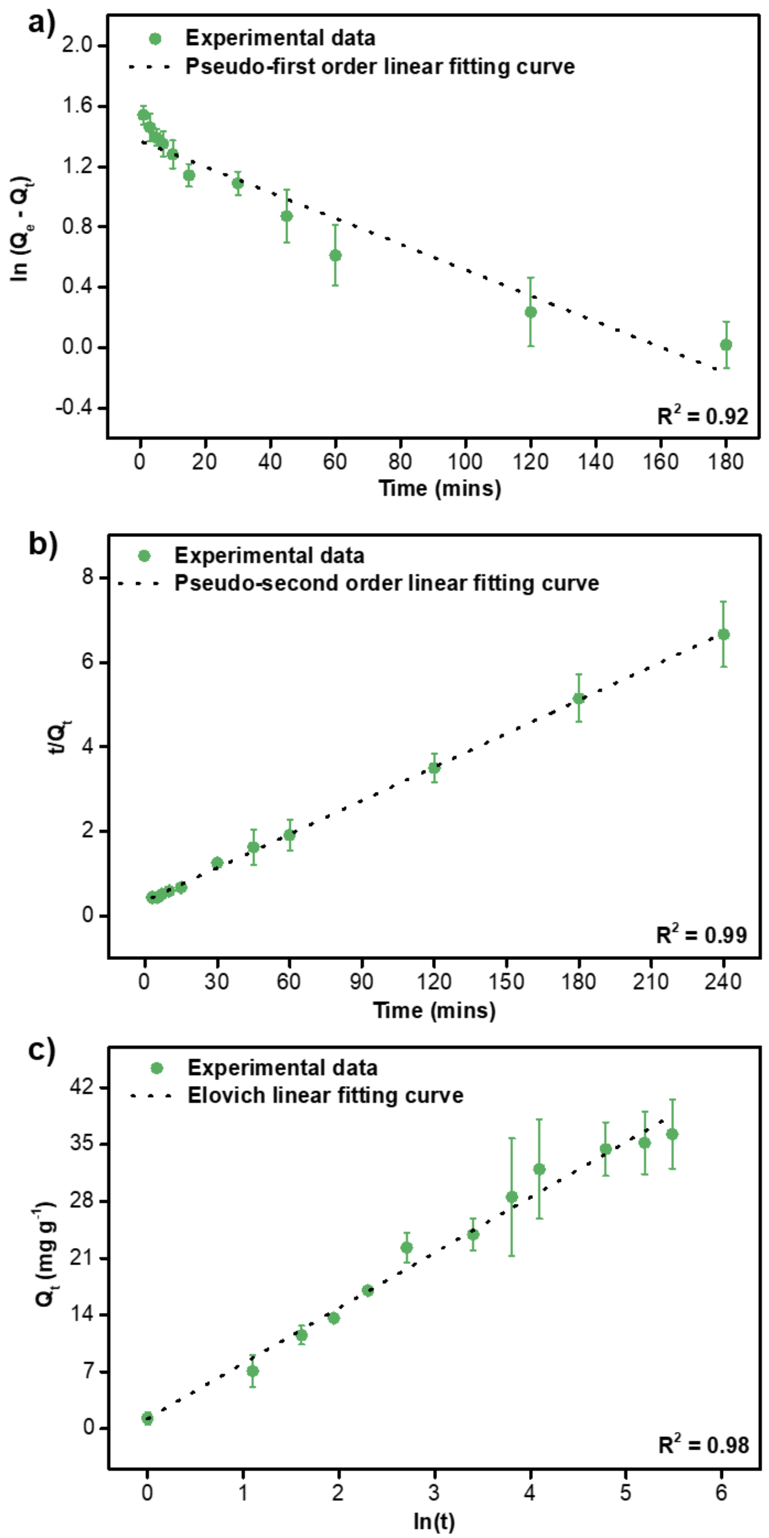

Figure S60. Experimental adsorption data fitting using a) pseudo-first order (**Equation S9**), b) pseudo-second order (**Equation S10**), and c) Elovich (**Equation S11**) linear models of Cd(II) adsorption isotherms onto Co-HITP. Conditions: m_{MOF} = 2 mg, $V_{solution}$ = 3 mL, and T = 298 K at different time intervals. Error bars represent standard deviation from the mean value of three independent experiments.

Parameters	Co-HHTP	Ni-HHTP	Cu-HHTP	Co-HITP
**Pseudo-first order kinetic isotherm**				
**K_1 (min^{-1})**	0.013	0.019	0.016	0.020
**Pseudo-second order kinetic isotherm**				
**K_2 ($g\ mg^{-1}\ min^{-1}$)**	2.5×10^{-4}	1.2×10^{-3}	2.7×10^{-3}	2.0×10^{-3}
**Elovich kinetic isotherm**				
**α ($J\ mol^{-1}$)**	16	23	26	8.2
**β ($L\ mg^{-1}$)**	0.046	0.092	0.16	0.15

Table S6. Linear kinetic modelling parameters for the adsorption of Cd(II) onto Co-HITP, Ni-HHTP, Cu-HHTP, and Co-HITP MOFs.

6.4 Kinetic isotherms of Hg^{2+} ions

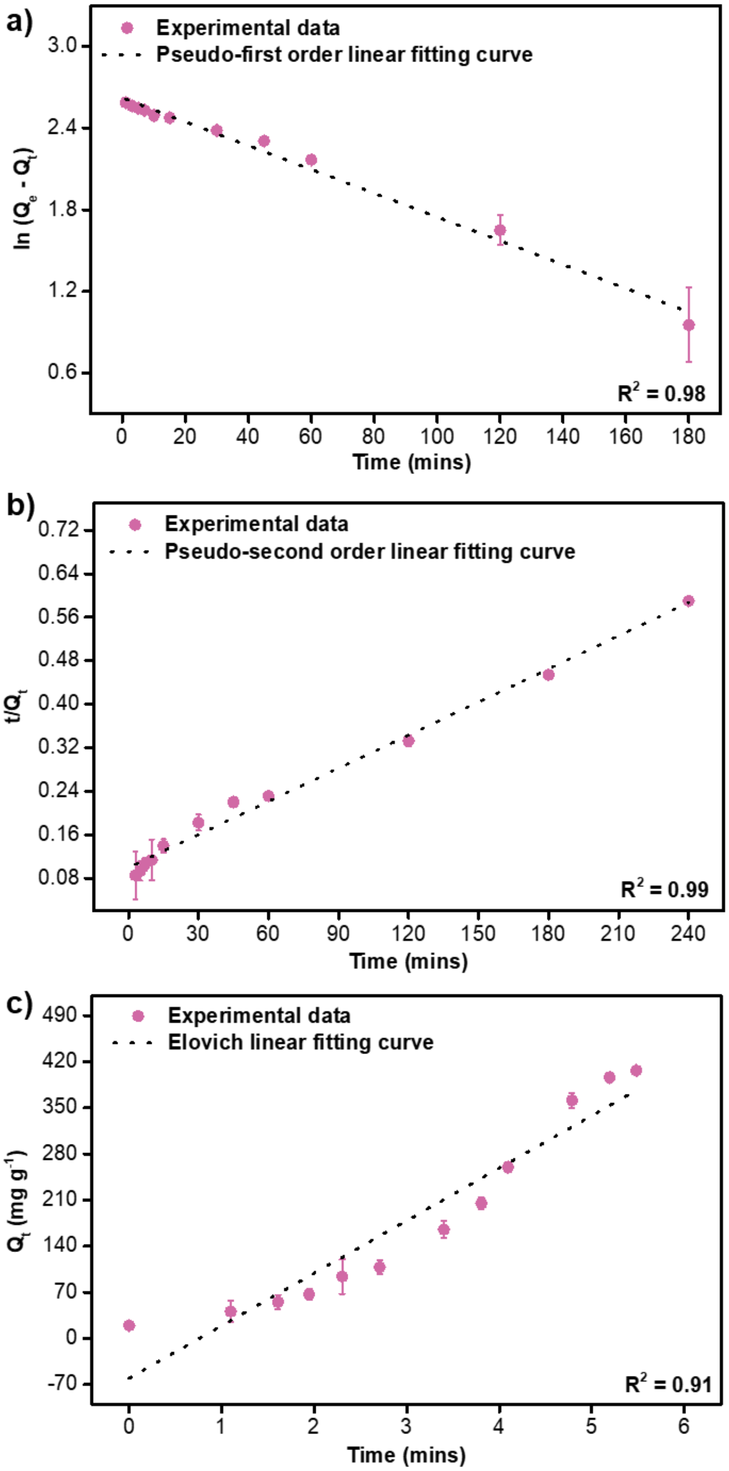

Figure S61. Experimental adsorption data fitting using a) pseudo-first order (**Equation S9**), b) pseudo-second order (**Equation S10**), and c) Elovich (**Equation S11**) linear models of Hg(II) adsorption isotherms onto Co-HHTP. Conditions: m_{MOF} = 2 mg, $V_{solution}$ = 3 mL, and T = 298 K at different time intervals. Error bars represent standard deviation from the mean value of three independent experiments.

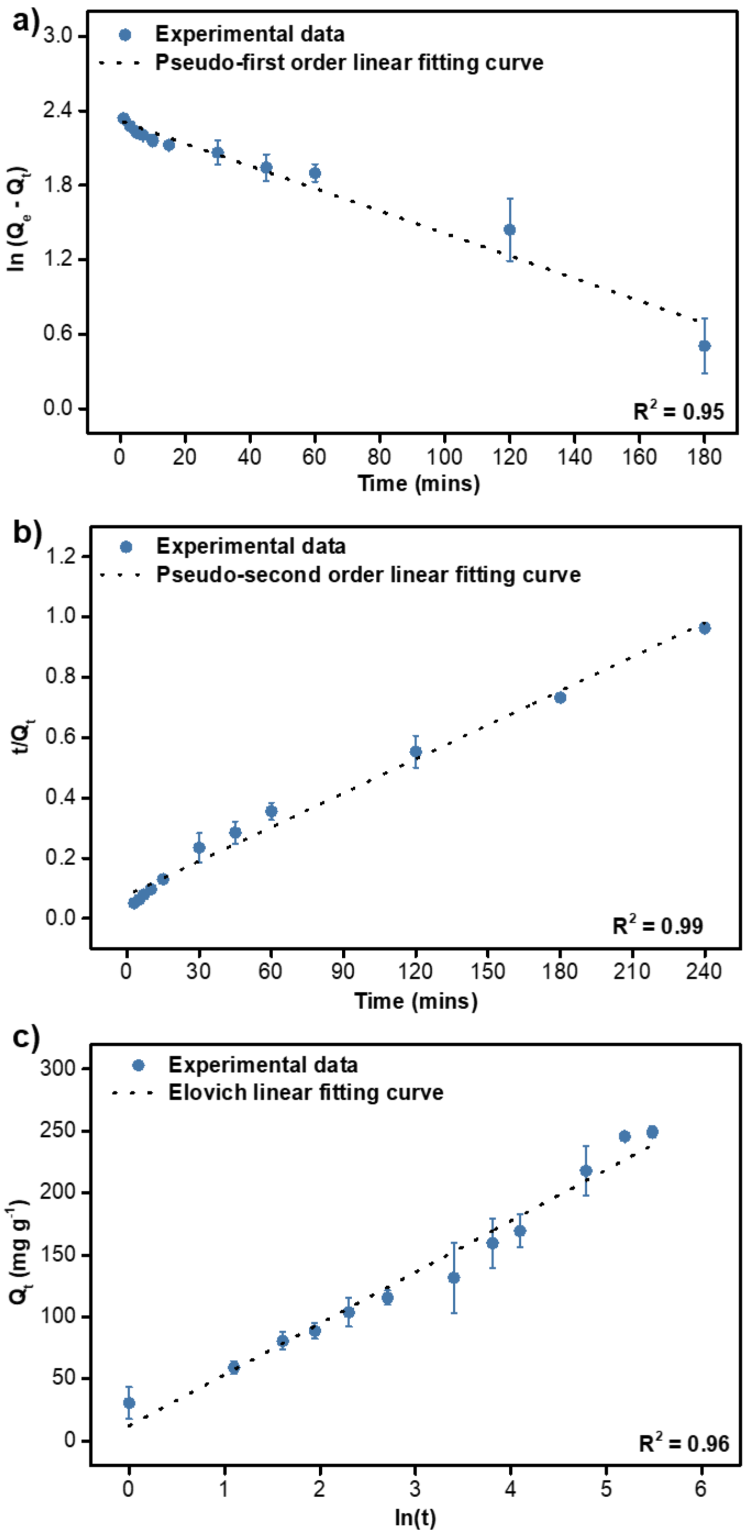

Figure S62. Experimental adsorption data fitting using a) pseudo-first order (**Equation S9**), b) pseudo-second order (**Equation S10**), and c) Elovich (**Equation S11**) linear models of Hg(II) adsorption isotherms onto Ni-HHTP. Conditions: m_{MOF} = 2 mg, $V_{solution}$ = 3 mL, and T = 298 K at different time intervals. Error bars represent standard deviation from the mean value of three independent experiments.

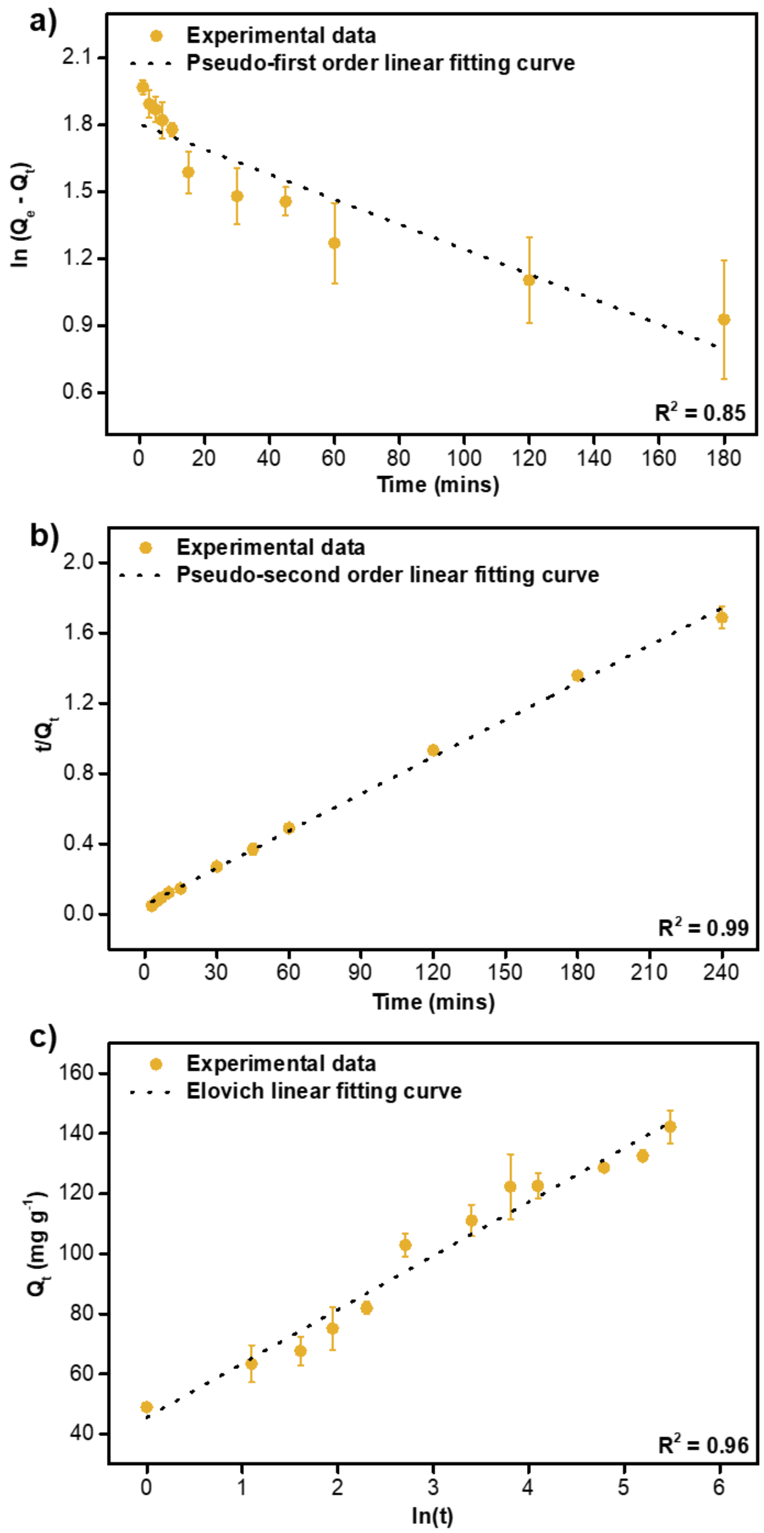

Figure S63. Experimental adsorption data fitting using a) pseudo-first order (**Equation S9**), b) pseudo-second order (**Equation S10**), and c) Elovich (**Equation S11**) linear models of Hg(II) adsorption isotherms onto Cu-HHTP. Conditions: m_{MOF} = 2 mg, $V_{solution}$ = 3 mL, and T = 298 K at different time intervals. Error bars represent standard deviation from the mean value of three independent experiments.

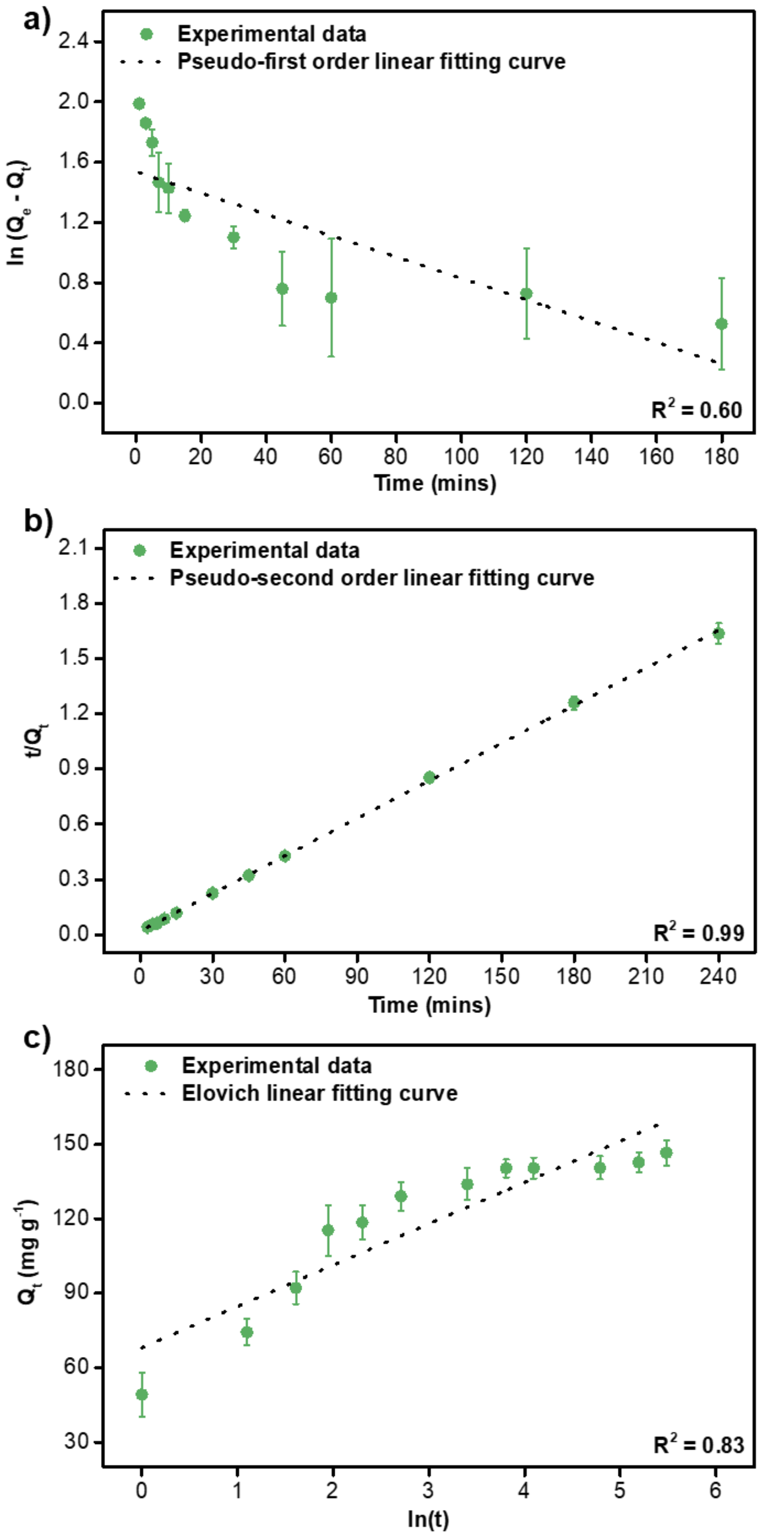

Figure S64. Experimental adsorption data fitting using a) pseudo-first order (**Equation S9**), b) pseudo-second order (**Equation S10**), and c) Elovich (**Equation S11**) linear models of Hg(II) adsorption isotherms onto Co-HITP. Conditions: m_{MOF} = 2 mg, $V_{solution}$ = 3 mL, and T = 298 K at different time intervals. Error bars represent standard deviation from the mean value of three independent experiments.

Parameters	Co-HHTP	Ni-HHTP	Cu-HHTP	Co-HITP
**Pseudo-first order kinetic isotherm**				
**K_1 (min^{-1})**	0.020	0.020	0.013	0.016
**Pseudo-second order kinetic isotherm**				
**K_2 (g mg^{-1} min^{-1})**	4.1 x 10^{-5}	1.8 x 10^{-4}	9.8 x 10^{-4}	2.5 x 10^{-3}
**Elovich kinetic isotherm**				
**α (J mol^{-1})**	37	55	23 x 10^{1}	99 x 10^{1}
**β (L mg^{-1})**	0.013	0.024	0.056	0.060

Table S7. Linear kinetic modelling parameters for the adsorption of Hg(II) onto Co-HITP, Ni-HHTP, Cu-HHTP, and Co-HITP MOFs.

6.5 Intra-particle diffusion model for Co-HHTP

The intraparticle diffusion model was used to simulate the adsorption diffusion kinetics and determine the number of mass transfer processes based on the Weber-Morris equation below:

$$q_t = K_p t^{0.5} + C \qquad \text{(Equation S12)}$$

Where K_p represents the intra-particle diffusion rate constant and C refers tp a constant defining the boundary-layer effect.[14]

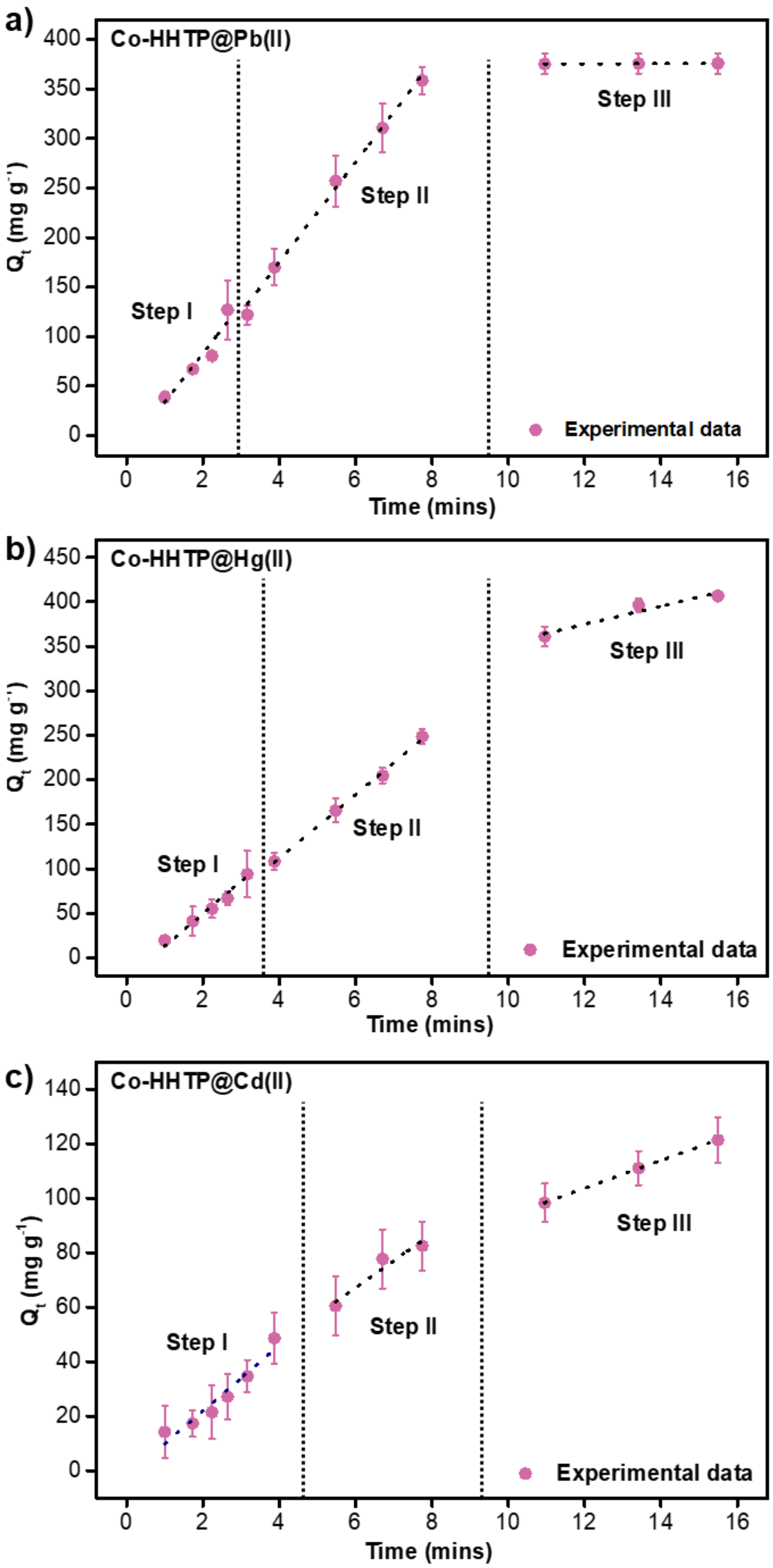

Figure S65. Intra-particle diffusion (**Equation S12**) models for Co-HHTP following adsorption of a) Pb(II), b) Hg(II), and c) Cd(II), respectively.

7. Mechanistic insights into MOF–Metal Ion Interactions

7.1 MOF-Hg(II) interactions

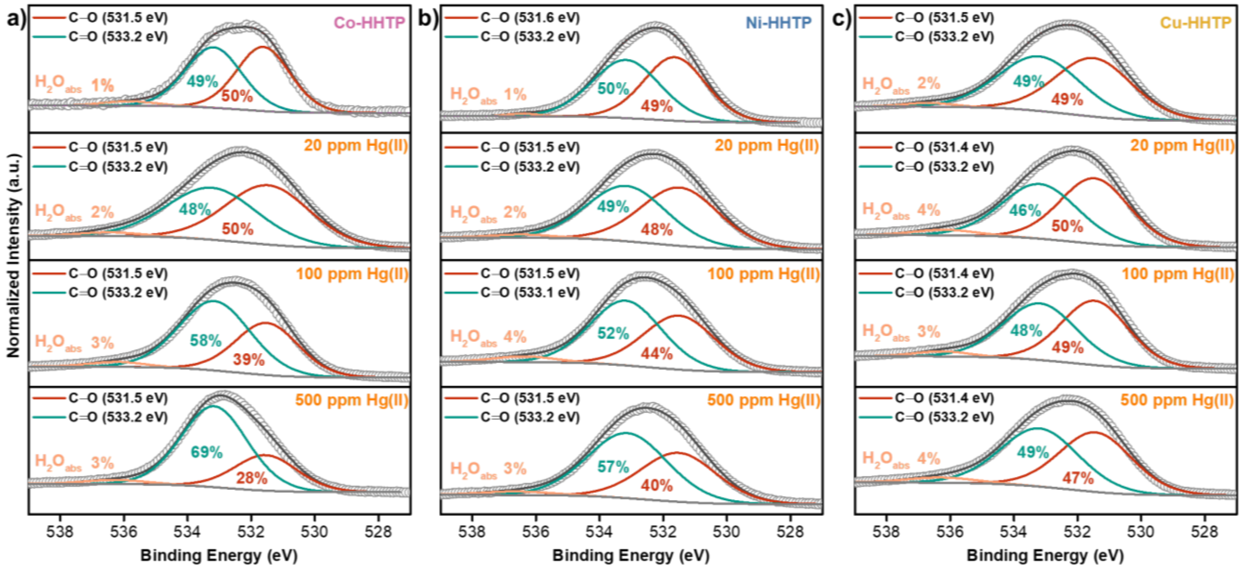

Figure S66. High-resolution XPS spectra of O 1s element of a) Co-HHTP, b) Ni-HHTP, and c) Cu-HHTP, before and after exposure of 20, 100, and 500 ppm of Hg(II).

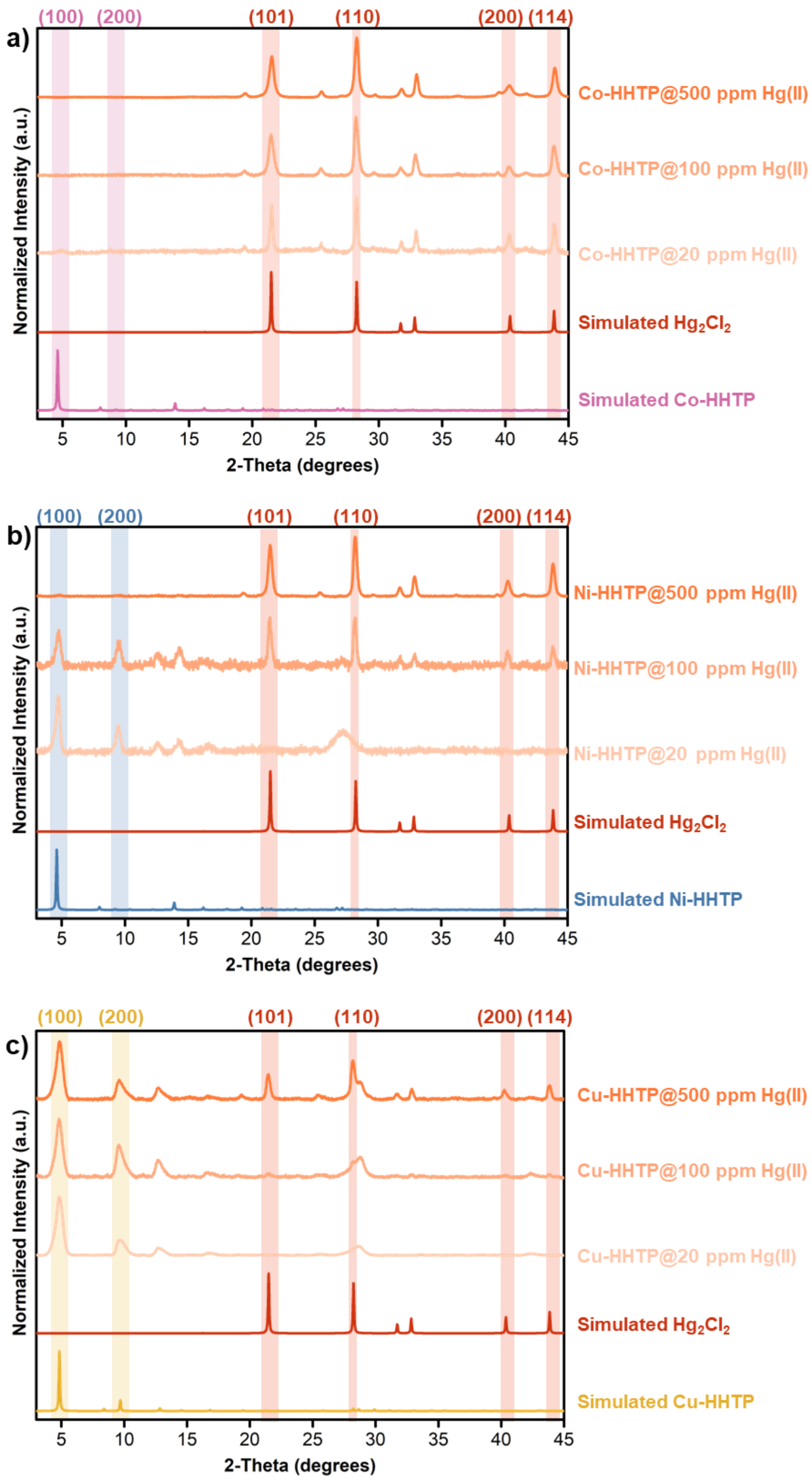

Figure S67. PXRD measurements of a) Co-HHTP, b) Ni-HHTP, and c) Cu-HHTP following exposure to 20 ppm, 100 ppm, and 500 ppm of $HgCl_2$ for 12 hours under ambient conditions.

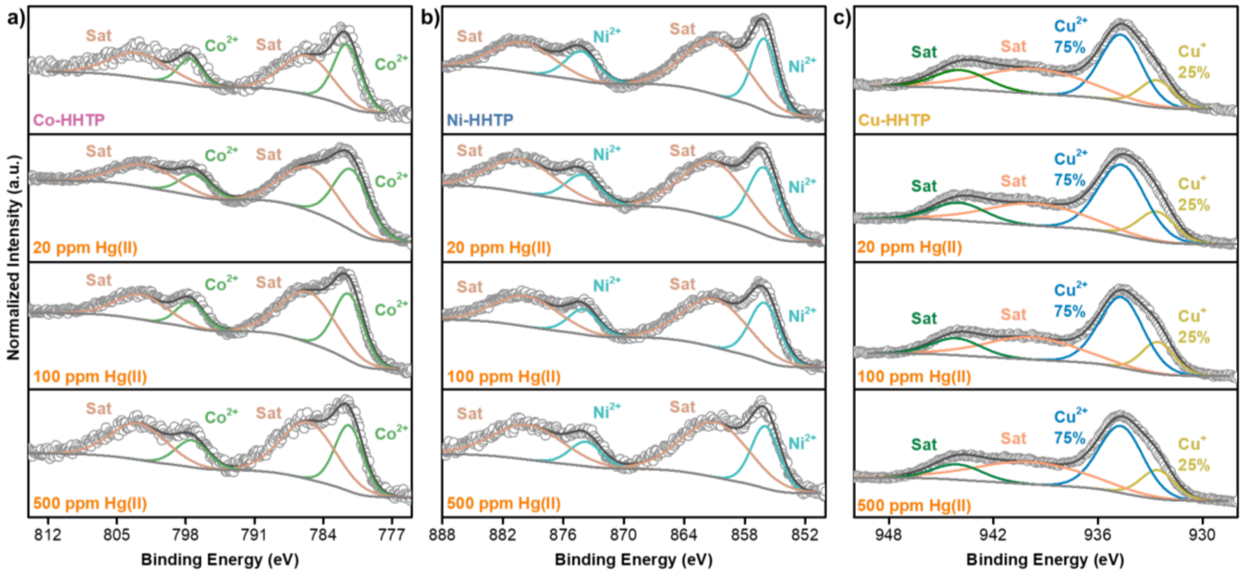

Figure S68. High-resolution XPS spectra of a) Co 2p, b) Ni 2p, and c) Cu 2p elements of Co-HHTP, Ni-HHTP, and Cu-HHTP, respectively, before and after exposure of 20, 100, and 500 ppm of Hg(II).

7.2 MOF-Pb(II) interactions

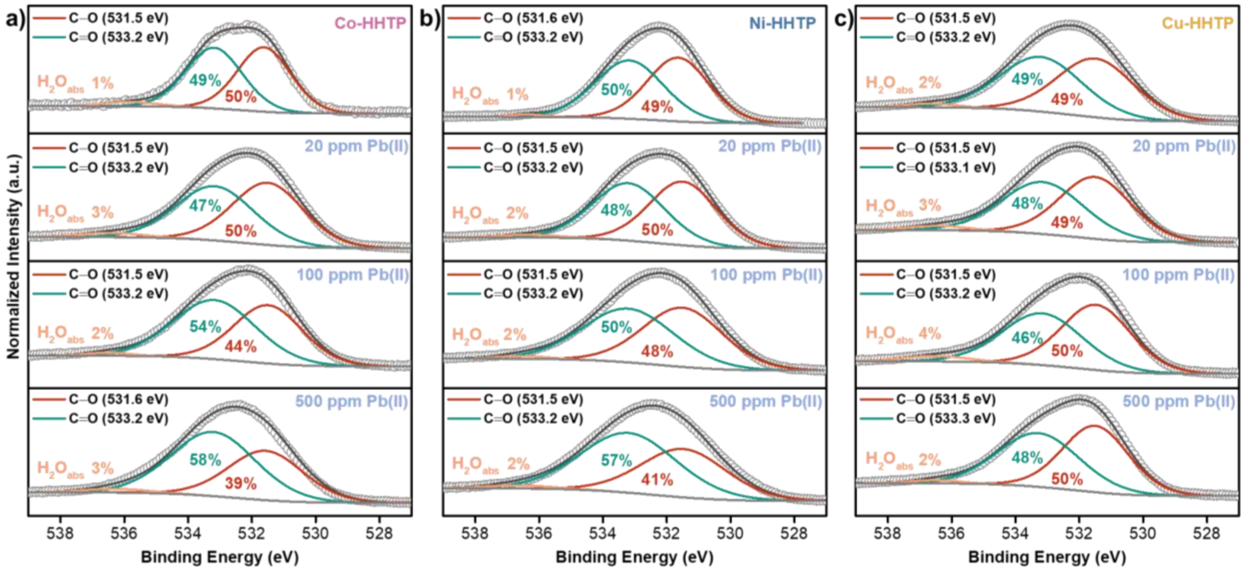

Figure S69. High-resolution XPS spectra of O 1s element of a) Co-HHTP, b) Ni-HHTP, and c) Cu-HHTP, before and after exposure of 20, 100, and 500 ppm of Pb(II).

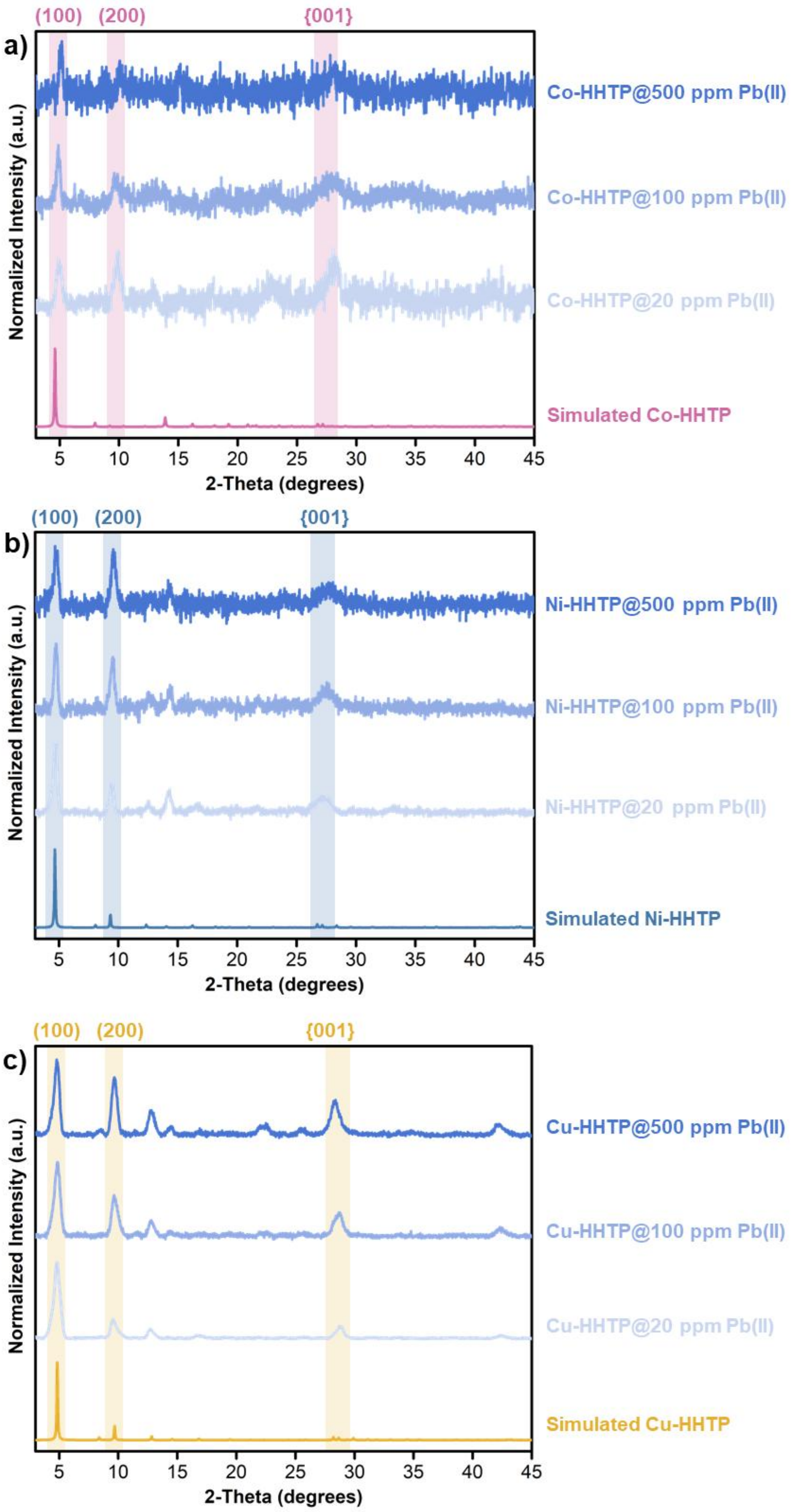

Figure S70. PXRD measurements of a) Co-HHTP, b) Ni-HHTP, and c) Cu-HHTP following exposure to 20 ppm, 100 ppm, and 500 ppm of $Pb(NO_3)_2$ for 12 hours under ambient conditions.

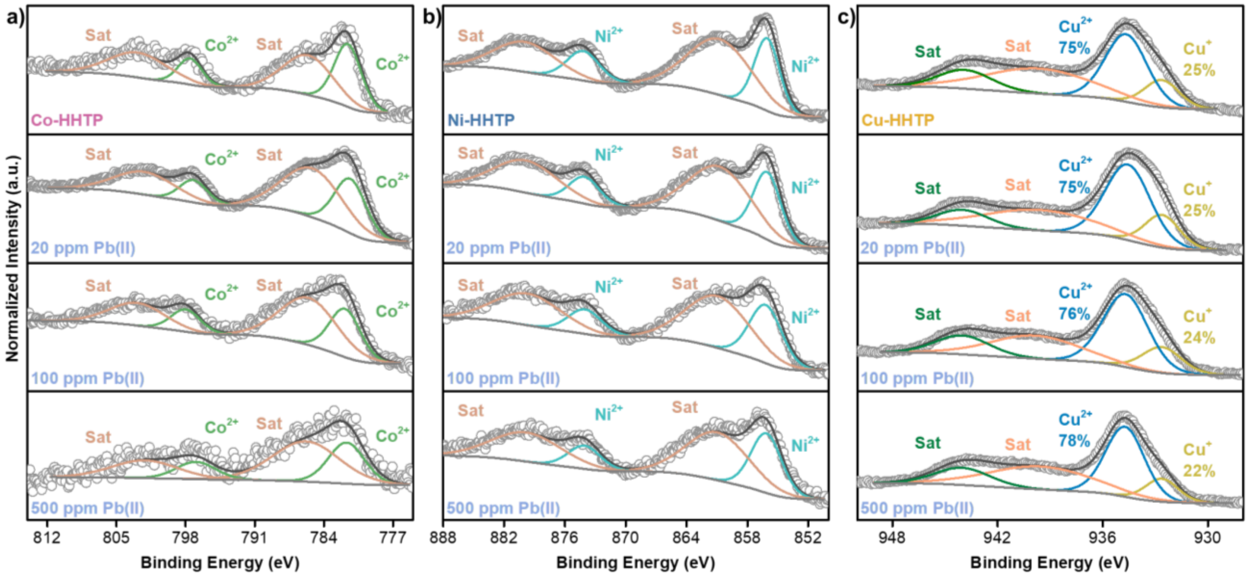

Figure S71. High-resolution XPS spectra of a) Co 2p, b) Ni 2p, and c) Cu 2p elements of Co-HHTP, Ni-HHTP, and Cu-HHTP, respectively, before and after exposure of 20, 100, and 500 ppm of Pb(II).

7.3 MOF-Cd(II) interactions

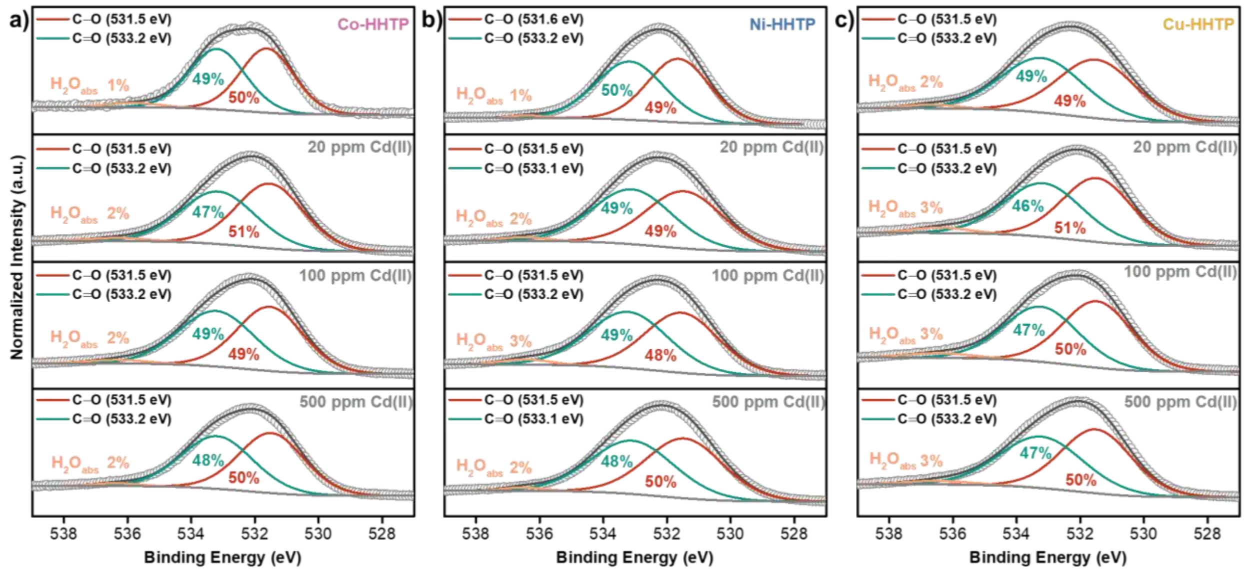

Figure S72. High-resolution XPS spectra of O 1s element of a) Co-HHTP, b) Ni-HHTP, and c) Cu-HHTP, before and after exposure of 20, 100, and 500 ppm of Cd(II).

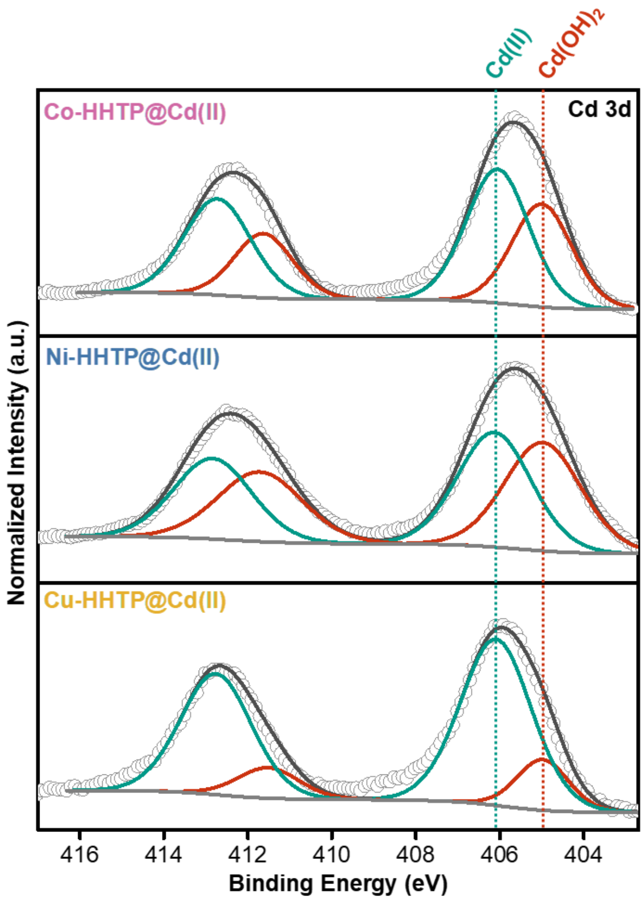

Figure S73. High-resolution XPS spectra of Cd 3d element of Co-HHTP, Ni-HHTP, and Cu-HHTP, after exposure to 500 ppm of Cd(II) for 12 hours under ambient conditions.

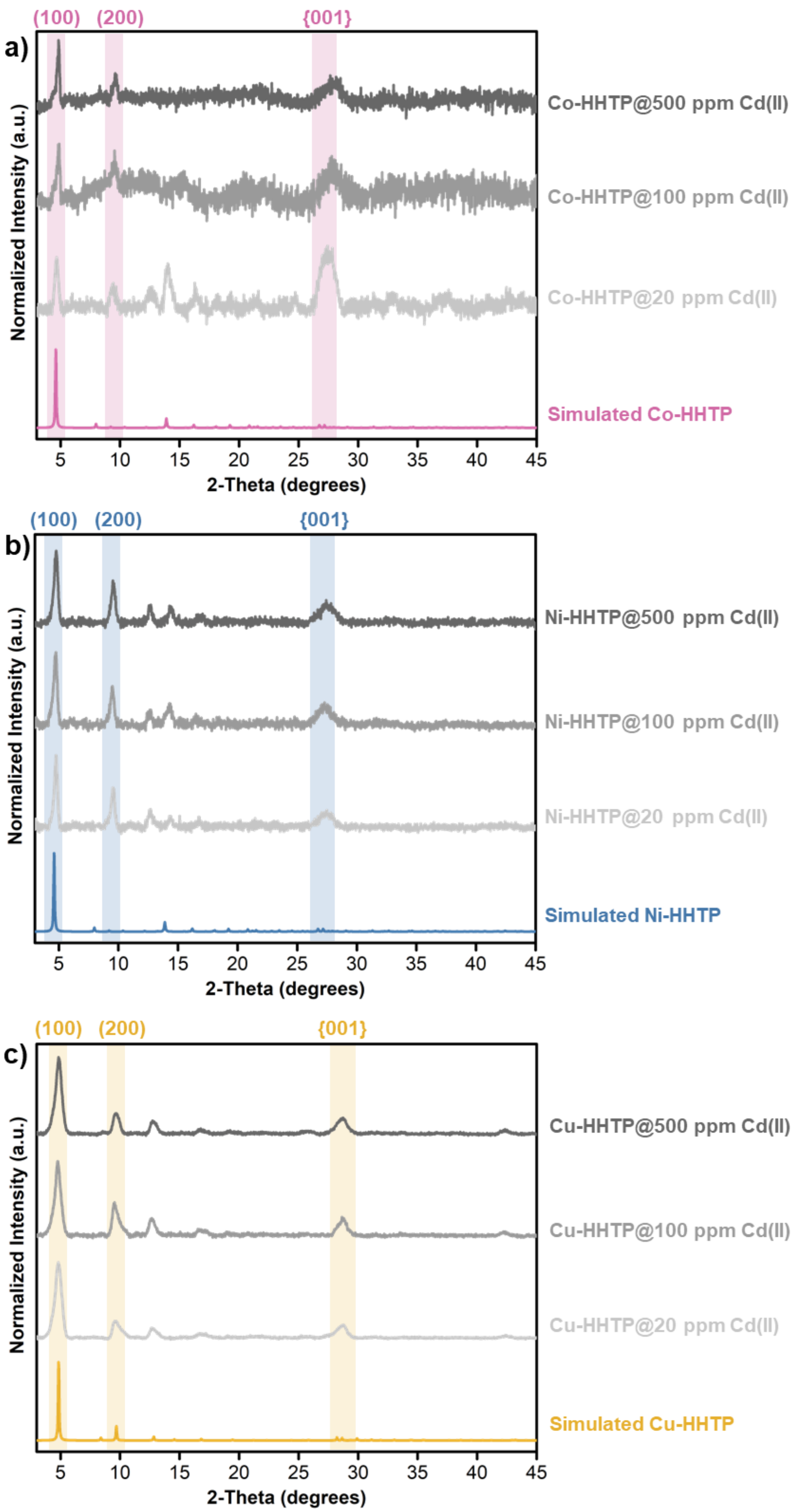

Figure S74. PXRD measurements of a) Co-HHTP, b) Ni-HHTP, and c) Cu-HHTP following exposure to 20 ppm, 100 ppm, and 500 ppm of $CdCl_2$ for 12 hours under ambient conditions.

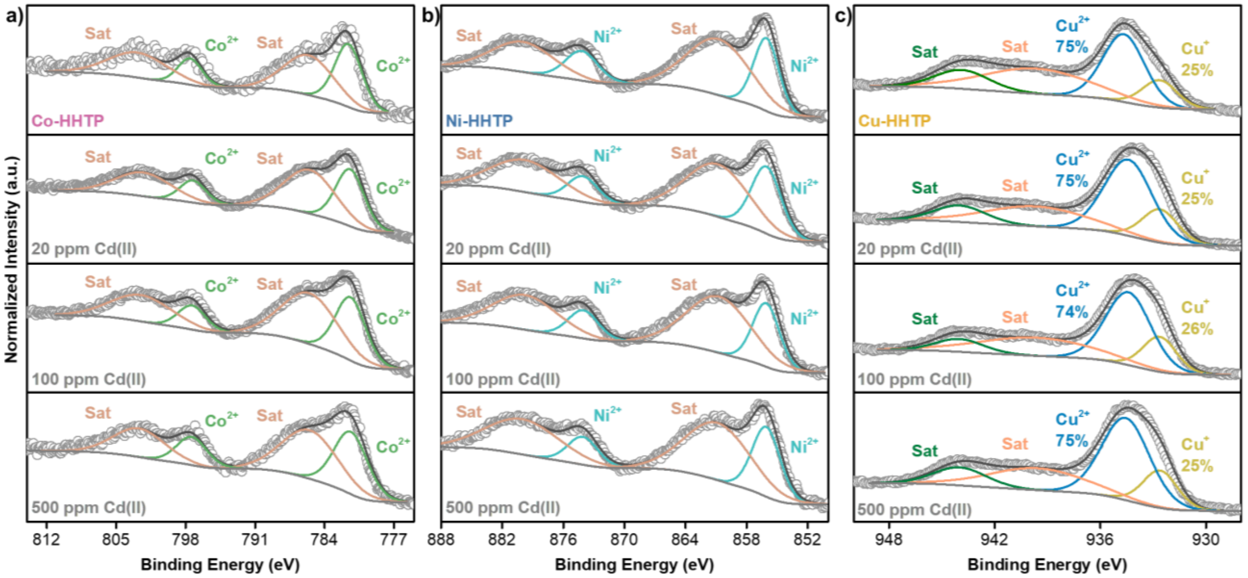

Figure S75. High-resolution XPS spectra of a) Co 2p, b) Ni 2p, and c) Cu 2p elements of Co-HHTP, Ni-HHTP, and Cu-HHTP, respectively, before and after exposure of 20, 100, and 500 ppm of Cd(II).

7.4 Additional characterization for Co-HHTP

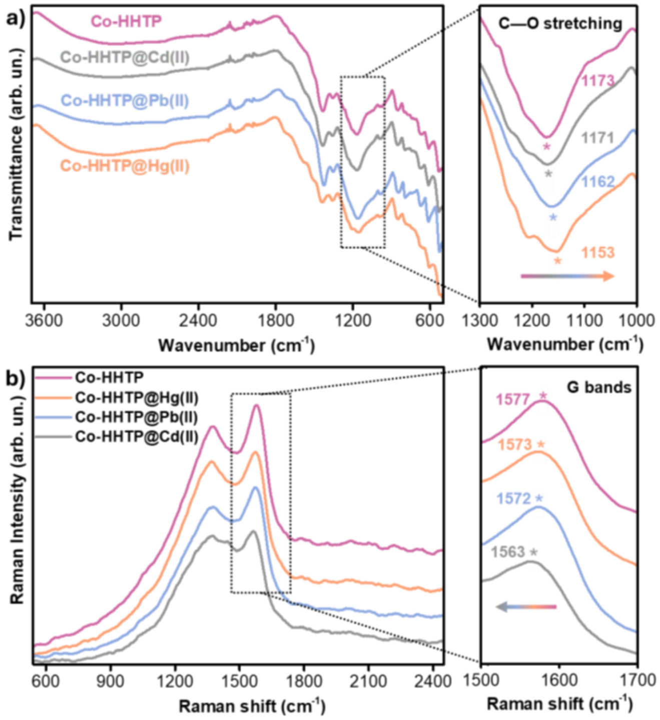

Figure S76. a) ATR-FTIR spectra of pristine Co-HHTP before and after adsorption of 100 ppm of Hg(II), Cd(II), and Pb(II). b) Raman spectra recorded using a 632.8 nm laser in the range of 600-2400 cm-1 portraying the graphitic (G) and defect (D) bands of Co-HHTP before and after removal of 100 ppm of Hg(II), Cd(II), and Pb(II).

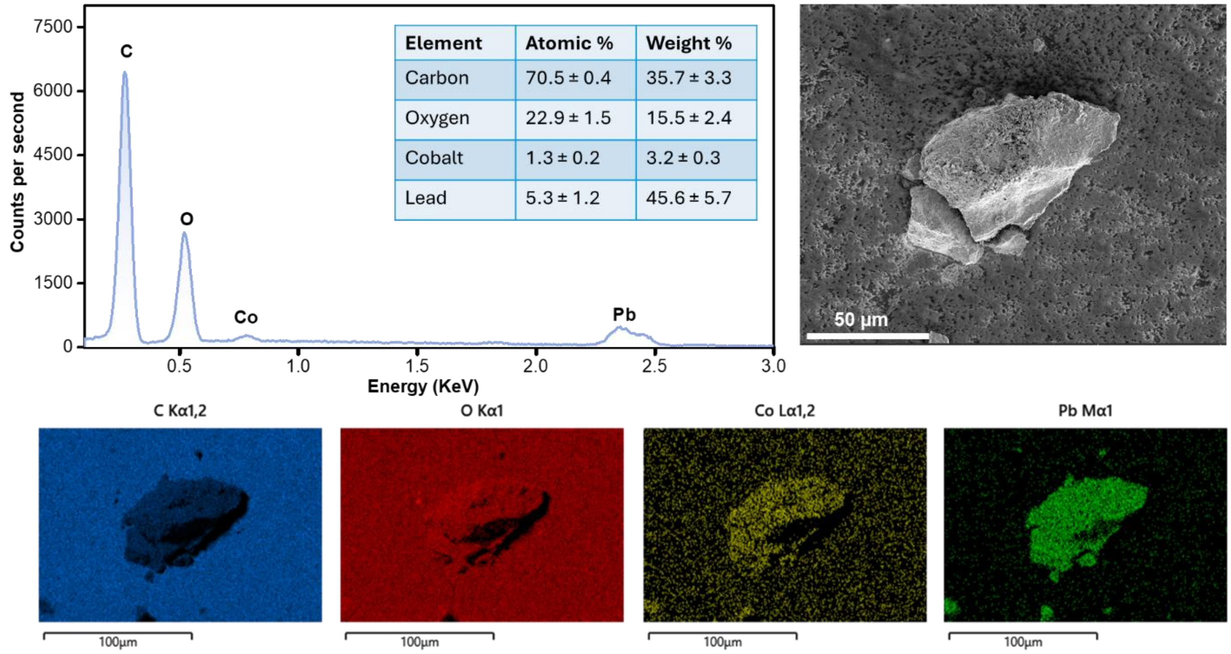

Figure S77. EDX spectrum and elemental mapping images of Co-HHTP following exposure to 100 ppm of Pb(II) ions for 4 hours.

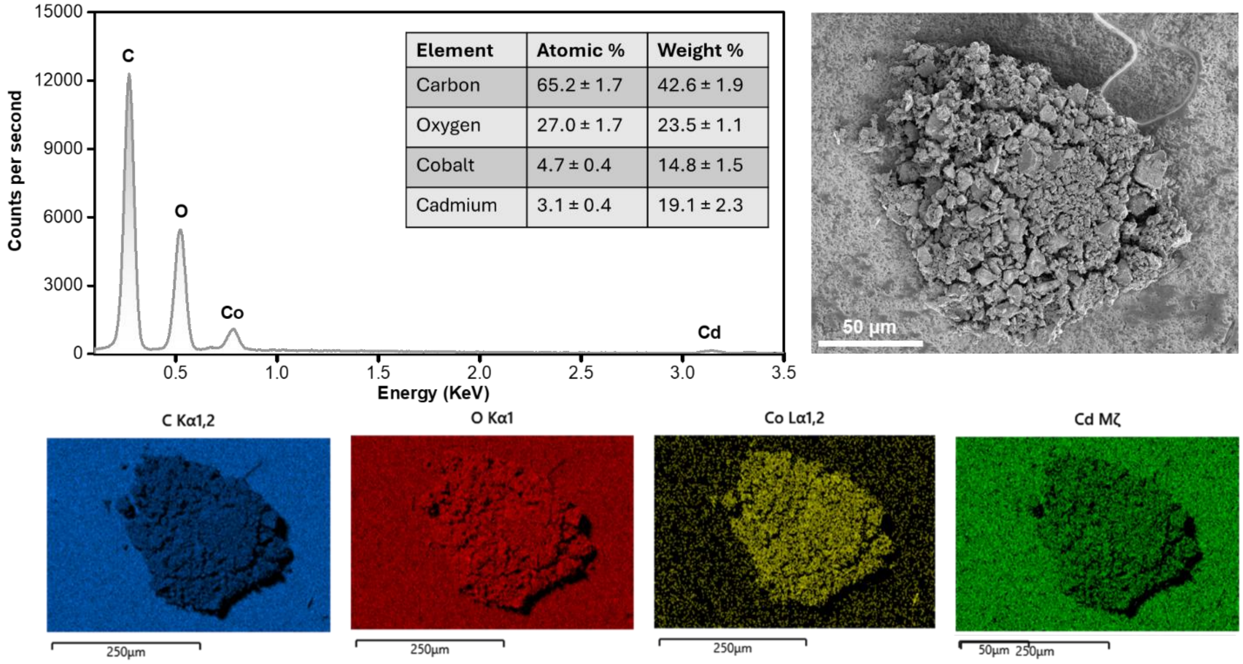

Figure S78. EDX spectrum and elemental mapping images of Co-HHTP following exposure to 100 ppm of Cd(II) ions for 4 hours.

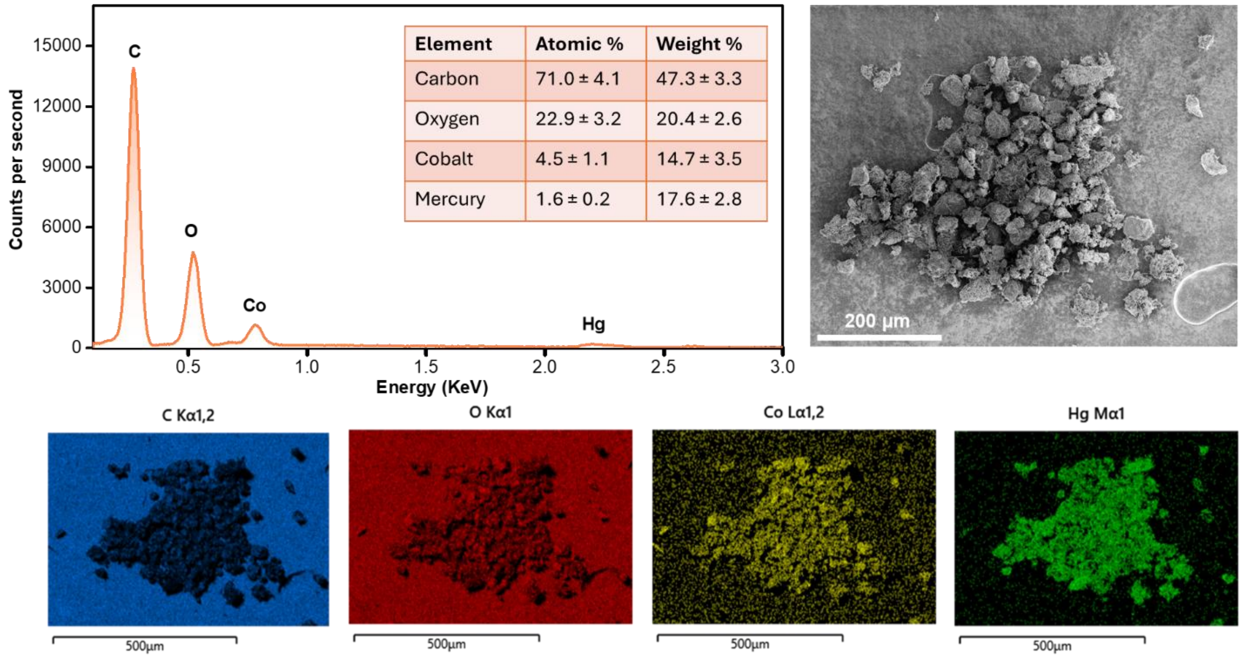

Figure S79. EDX spectrum and elemental mapping images of Co-HHTP following exposure to 100 ppm of Hg(II) ions for 4 hours.

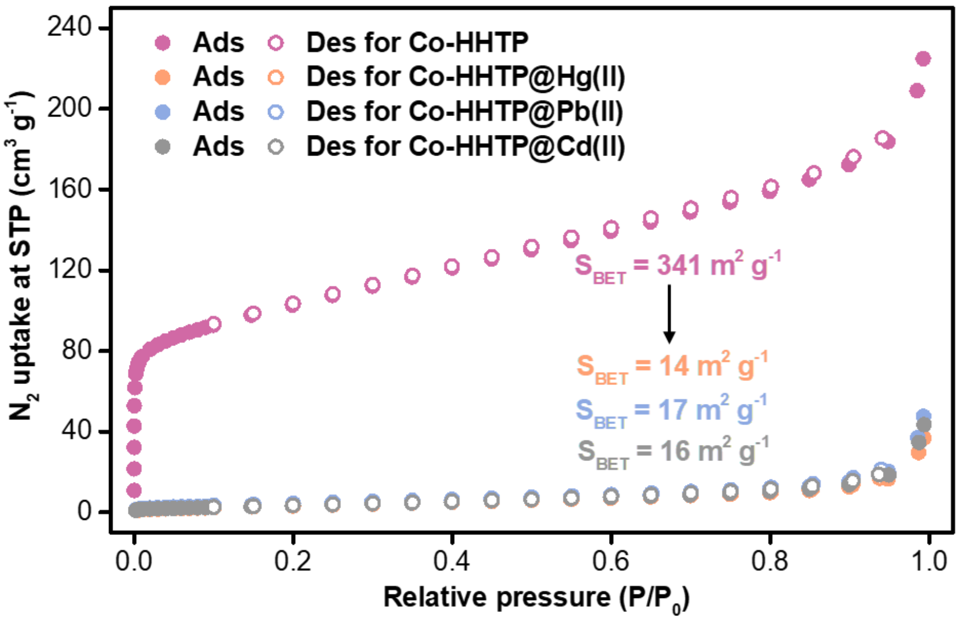

Figure S80. Nitrogen sorption curves (filled circles: N_2 adsorption, open circles: N_2 desorption) for Co-HHTP before and after adsorption at standard temperature pressure (STP). The BET surface areas by the gas adsorption analysis is found to be 341 m^2 g^{-1} for Co-HHTP, 14 m^2 g^{-1} for Co-HHTP@Hg(II), 17 m^2 g^{-1} for Co-HHTP@Pb(II), and 16 m^2 g^{-1} for Co-HHTP@Cd(II).

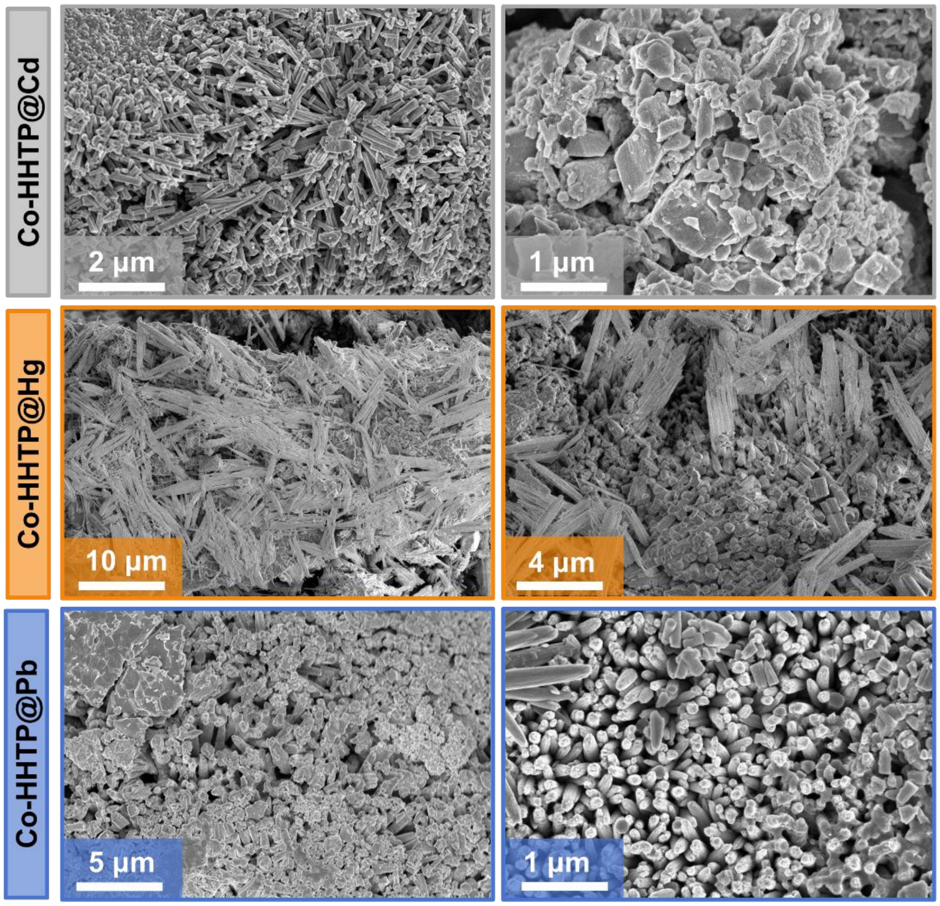

Figure S81. SEM micrographs of Co-HHTP at different locations and magnifications following exposure to 100 ppm of Cd^{2+}, Hg^{2+}, and Pb^{2+} ions for 4 hours.

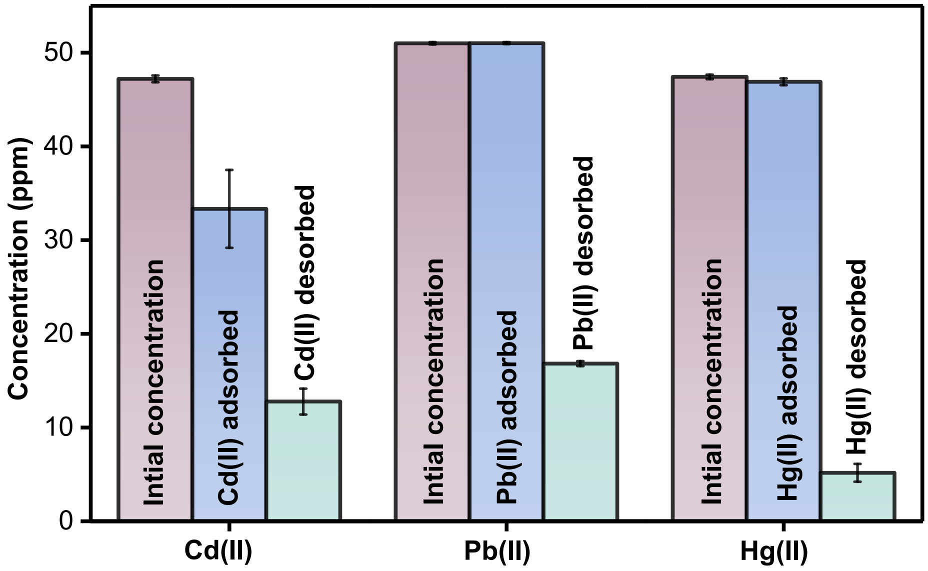

Figure S82. Adsorption-desorption cycle of Co-HHTP towards 50 ppm of Cd(II), Pb(II), and Hg(II) contaminants. 4 mM of HCl was used to desorb the contaminants.

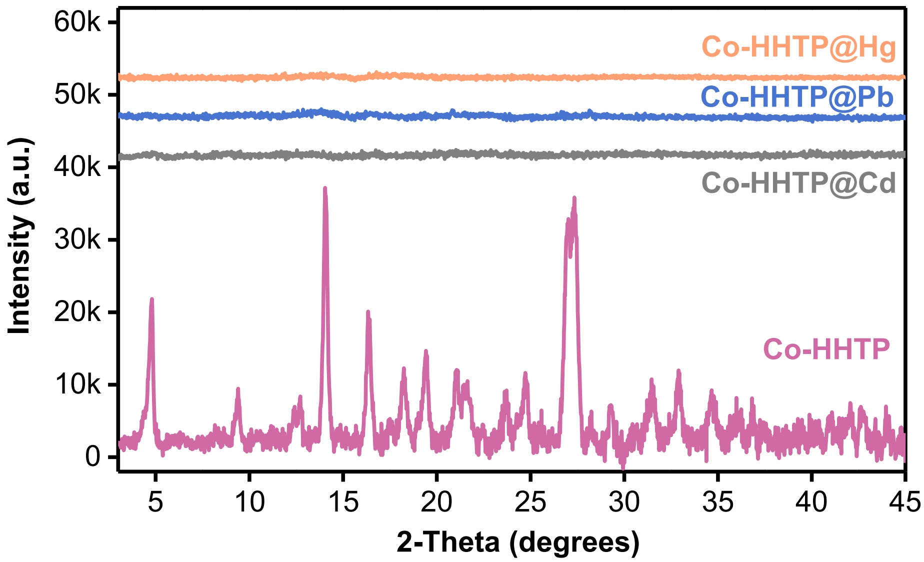

Figure S83. PXRD patterns of Co-HHTP following desorption of heavy metals via acid treatment using 4 mM of HCl.

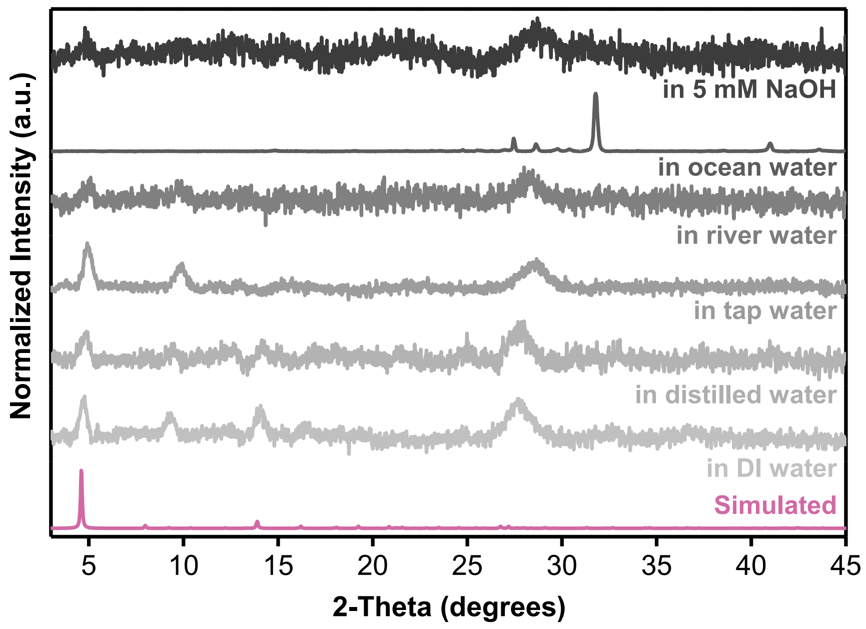

Figure S84. PXRD patterns of bulk Co-HHTP after soaking in various water matrices for 48 hours.

Our adsorption experiments throughout this study focused on single-metal systems. However, previous reports on MOF-based adsorbents and inorganic nanoparticles have demonstrated their ability to co-adsorb multiple heavy-metal ions, such as Pb^{2+}, Cd^{2+}, and Hg^{2+}, from mixed solutions, albeit with affinities and capacities that differ from single-component systems.[15-19] Given that Co-HHTP operates through a synergistic combination of chemisorption, physisorption, and redox interactions, we anticipate similar trends. Specifically, the superior removal efficiencies observed for Hg^{2+} and Pb^{2+} relative to Cd^{2+} stem from their ability to undergo charge-transfer and redox interactions with Co-HHTP, leading to partial reduction and, in the case of Hg^{2+}, precipitation of insoluble mercury(I) chloride species. The formation of these reduced species may occupy or block the adsorptive active sites within the MOF, thereby limiting subsequent uptake of Cd^{2+}. The latter primarily interacts with the framework through coordination and electrostatic interactions rather than redox processes, suggesting a lower adsorption capacity in mixed-ion environments where both, Hg^{2+} and Pb^{2+}, are present. Therefore, while Co-HHTP is expected to retain multi-ion adsorption capability, the capacities measured in single-metal systems should be interpreted cautiously when extrapolated to complex water matrices containing coexisting heavy metals.

8. Molecular modeling

The atomic coordinates of Co-HHTP MOF were obtained from the CIF file of Yaghi and colleagues,[20] whereas those of the Cu-HHTP MOF from a previous modeling investigation,[21] where the CIF, derived by Gittins et al.[22] was used as the input structure. All the structures were simulated at the density functional theory level (DFT) using the Quantum Espresso (QE) package,[23] Projected Augmented Wave (PAW) pseudopotentials available in the QE database, and the generalized gradient approximation (GGA) with the Perdew–Burke–Ernzerhof (PBE) functional.[24] Dispersion corrections were added according to the Grimme-D3 parametrization implemented in the QE package.[25] Wave function and charge density cutoffs for the plane-wave basis sets of 60 and 600 Ry were employed, respectively. Single-particle wave functions were

calculated spin-unrestricted by applying a Gaussian smearing of the one-particle levels with a width of 0.002 Ry. Periodic boundary conditions were used in all directions. The lattice constants of the cells were optimized by applying the variable-cell algorithm on the bare MOFs; DFT-optimized lattice constants were then used to investigate the interaction with adsorbate species in the presence of water molecules by performing local relaxations. Periodic boundary conditions were applied in all directions. In the case of bilayers, the reciprocal space was sampled using a (1 x 1 x 3) k-point grid, whereas in the case of thicker cells with four layers, the reciprocal space was sampled at the gamma point only. The optimized lattice parameters of the Co-HHTP phase resulted in good agreement with the experimental measurements, providing values of 21.65, 22.25 and 6.61 Å for the length of the lattice vectors (to be compared with 22.13, 22.13 and 6.65 Å), and 89.3, 89.1 and 119.8° (to be compared with 90, 90 and 120°) for the unit cell angles. The extent of agreement between the experimental and simulated structures of the Cu-HHTP bare MOF is reported in Zhong et al.[21]

Electrostatic mapping was performed by using the Gaussian16 software,[26] considering finite-size model systems. Basis sets of 6-31G(d,p) quality were used for the lighter elements, whereas def2-TZVPP basis sets were employed for the Cu and Co species. Charge analysis was performed with an NBO scheme, as implemented in the g16 software.

These two MOFs have 2D sheets with stoichiometry of $M_3(HHTP)_2$, where each catechol unit coordinates three metal species via two oxygen anions. In the case of Cu-HHTP, each Cu cation is tetra-coordinated (see **Figure S86A**), whereas in the case Co-HHTP, the presence of two additional water molecules per Co, coordinated via their oxygen to the metal center, makes the coordination environment of each Co cation octahedral (see **Figure S86B**). In Cu-HHTP, the 2D sheets are arranged as a monoclinic slipped-parallel (SP) structure,[22] although, as thoroughly discussed in Mirica and colleagues,[21] an alternative organization with open metal sites (OMS) can result as a consequence of the shifting of the individual stacked layers to an fcc crystal of ABC

type. This latter configuration was identified by RMD (Reactive Molecular Dynamics) in a previous investigation.[21] QC calculations on a reduced 4L-ABCB unit cell demonstrated a close competition between the two types of morphologies. In the case of Co-HHTP MOF, the $Co_3(HHTP)_2$ sheets alternate with Co_3(HHTP) 0D complexes, where each Co cation is coordinated at each vertex of the catechol unit with the addition of four water molecules in an octahedral local environment (see **Figure S86C**). As shown in the work of Stolz et al,[27] the catechol units in the 0D complexes and in the 2D sheets are staggered, whereas the 2D sheets adopt a slipped parallel (quasi-eclipsed) arrangement between them. Both MOFs form long channels perpendicular to the planes of the catechol networks; the walls of these channels are terminated by negative oxygen ions only in the case of the Cu-HHTP system, whereas in the case of the Co-HHTP MOF water molecules belonging to the 0D structures (exposing hydrogen atoms available as donors of hydrogen bonding) can also be found.

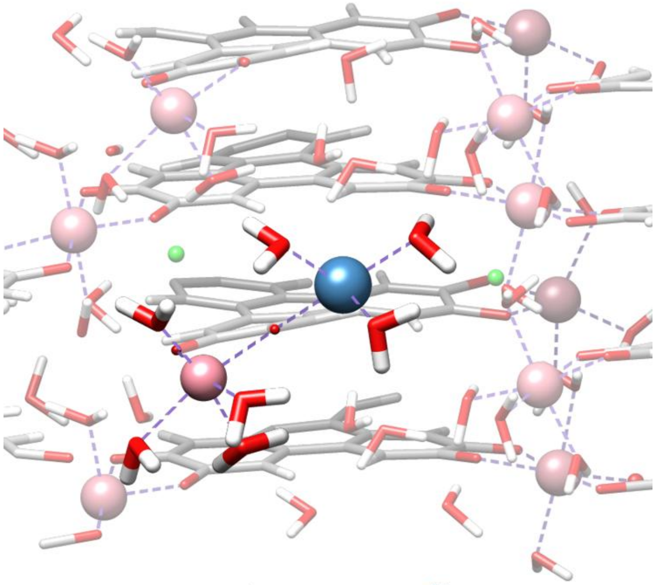

Figure S85. Hg^{2+} interacting with the Co-HHTP MOF, configuration C1, see Table 1 and main text. Color code: C gray, O red, H white, Co pink, Hg blue.

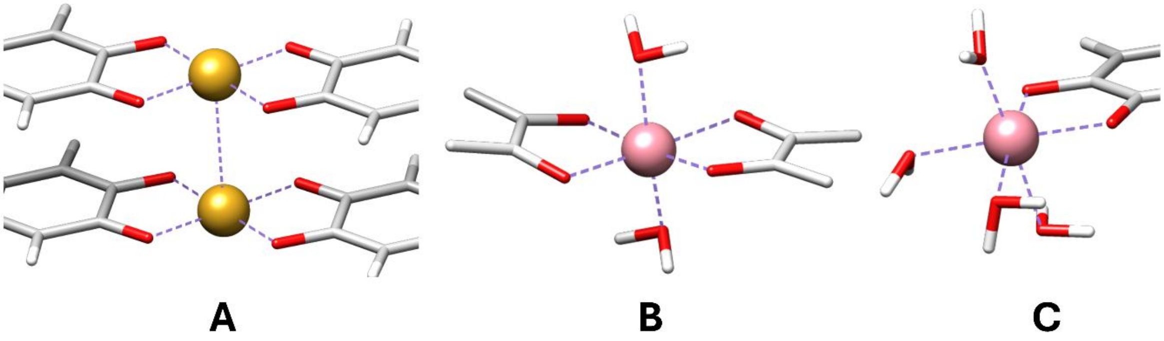

Figure S86. (A) tetra-coordinated Cu cation from $Cu_3(HHTP)_2$ in a local slipped-parallel arrangement morphology; (B) hexa-coordinated Co cation from $Co_3(HHTP)_2$, where two water molecules and four catechol oxygens complete the coordination shell; (C) hexa-coordinated Co cation from the 0D structure of the Cu-HHTP MOF, where four water molecules and two catechol oxygens complete the coordination shell. Color code: C gray, O red, H white, Cu light yellow, Co pink.

9. Fabrication of Co-HHTP on textile fabrics

9.1 Synthetic procedures for the preparation of Co-HHTP on textiles

We first prepared the ligand solution by dissolving 65 mg of HHTP (0.2 mmol, 1 eq) in 0.8 mL of 1,3-dimethyl-2-imidazolidinone in a 20 mL vial. The metal solution was then prepared by dissolving 70 mg of $Co(OAc)_2$ (0.28 mmol, 1.4 eq) in 3.2 mL of DI water. After separately sonication each solution for 10 minutes, the metal solution was added dropwise to the ligand solution, and the resulting mixture was shaken for a few seconds. A 1.5 x 1.5 cm^2 piece of textile fabric (cotton, silk, or polyester) was then immersed in the vial, and the mixture was heated on a hotplate set at 75 °C for 14 hours, with leaving the vial uncapped to allow air diffusion into the solution. After cooling, the fabric swatch was removed from the vial and washed by submerging it in 15 mL of DI water for 10 minutes, followed by 15 mL of acetone for another 10 minutes. This washing process was repeated five times, to ensure the removal of any residual starting materials and byproducts. The washed textile swatch was then drip-dried in air overnight (12 hours) before use in any adsorption experiments.

9.2 Optimization efforts for depositing Co-HHTP on textiles

Table S8. Summary of the synthetic conditions used for depositing Co-HHTP on cotton textile.

Entry	Solvent (v/v)	HHTP Conc.	Additives (e.q.)	Metal salt Conc.	MOF loading per 1 cm^2
1	DMI/H_2O (1/4)	12 mM	N/A	$Co(OAc)_2$ 24 mM	1.6
2	H_2O	50 mM	N/A	$Co(OAc)_2$ 70 mM	0.1
3	H_2O	50 mM	CH_3COONa (25)	$Co(OAc)_2$ 70 mM	1.5
4	H_2O	50 mM	CH_3COONa (50)	$Co(OAc)_2$ 70 mM	1.1
5	EtOH/H_2O (1/1)	50 mM	N/A	$Co(OAc)_2$ 70 mM	0.9
6	DMI/H_2O (1/4)	75 mM	N/A	$Co(OAc)_2$ 150 mM	3.1
7	DMI/H_2O (1/4)	50 mM	N/A	$CoCl_2$ 70 mM	0.7
8	H_2O	50 mM	NH_4OH (20)	$Co(OAc)_2$ 70 mM	0.8
9	DMI/H_2O (2/3)	50 mM	N/A	$Co(OAc)_2$ 70 mM	2.5
10	DMI/H_2O (1/9)	50 mM	N/A	$Co(OAc)_2$ 70 mM	3.4
11	DMI/H_2O (1/4)	25 mM	N/A	$Co(OAc)_2$ 100 mM	1.5
12	DMI/H_2O (1/4)	115 mM	N/A	$Co(OAc)_2$ 100 mM	4.2
13	DMI/H_2O (1/4)	50 mM	N/A	$Co(NO_3)_2$ 70 mM	0.4
**14**	**DMI/H_2O (1/4)**	**50 mM**	**N/A**	**$Co(OAc)_2$ 70 mM**	**3.0**

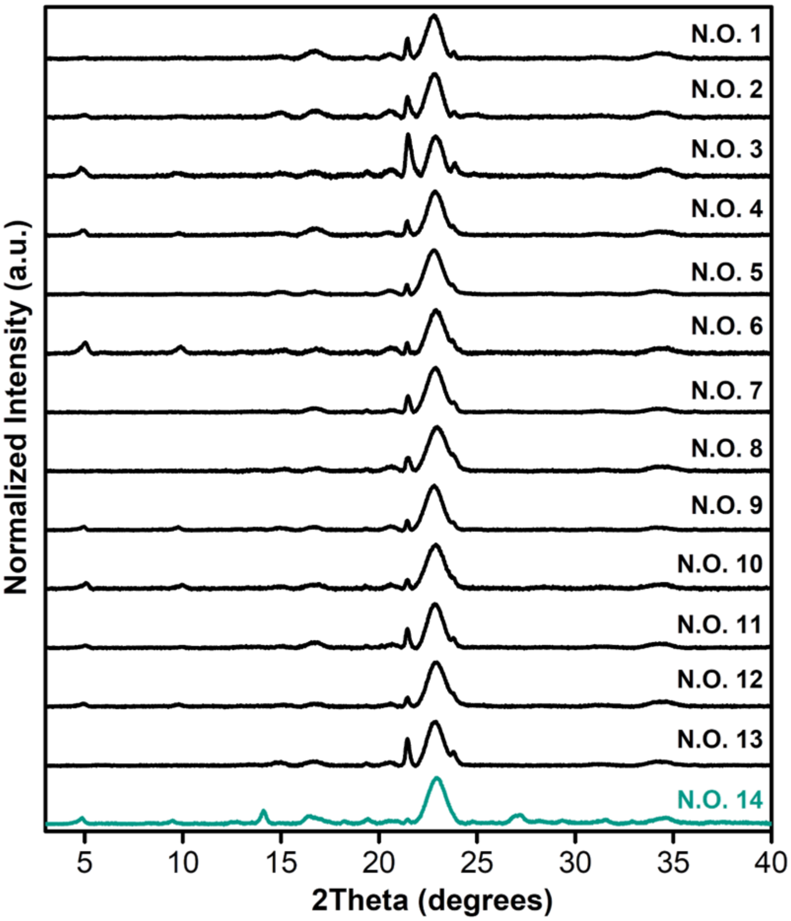

Figure S87. Comparison of PXRD patterns (λ = 1.5406 Å) of Co-HHTP@Cotton synthesized from the conditions listed in Table S8.

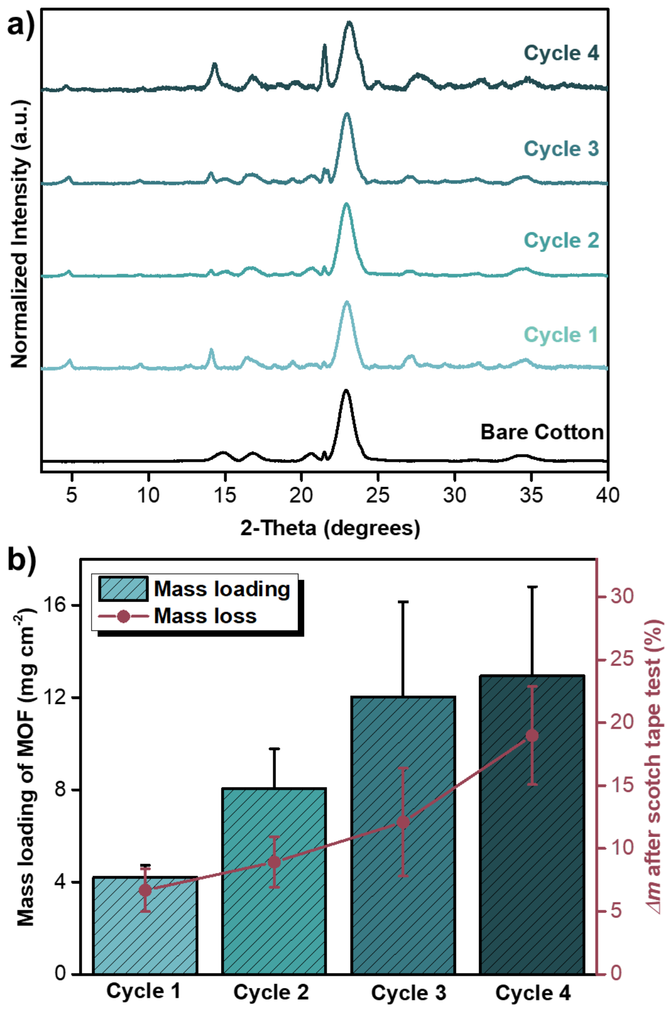

Figure S88. a) PXRD patterns and b) mass loading and loss of Co-HHTP@cotton textile after multiple solvothermal deposition cycles determined by scotch tape test.

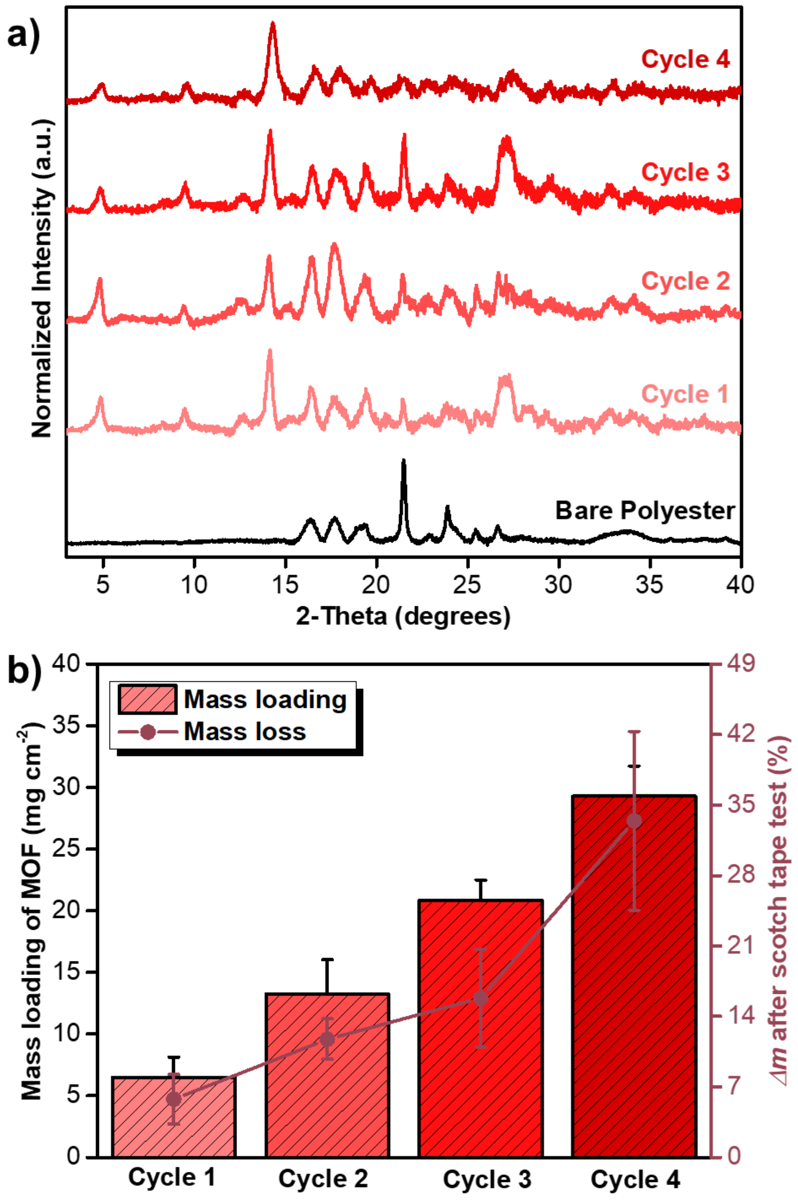

Figure S89. a) PXRD patterns and b) mass loading and loss of Co-HHTP@polyester textile after multiple solvothermal deposition cycles determined by scotch tape test.

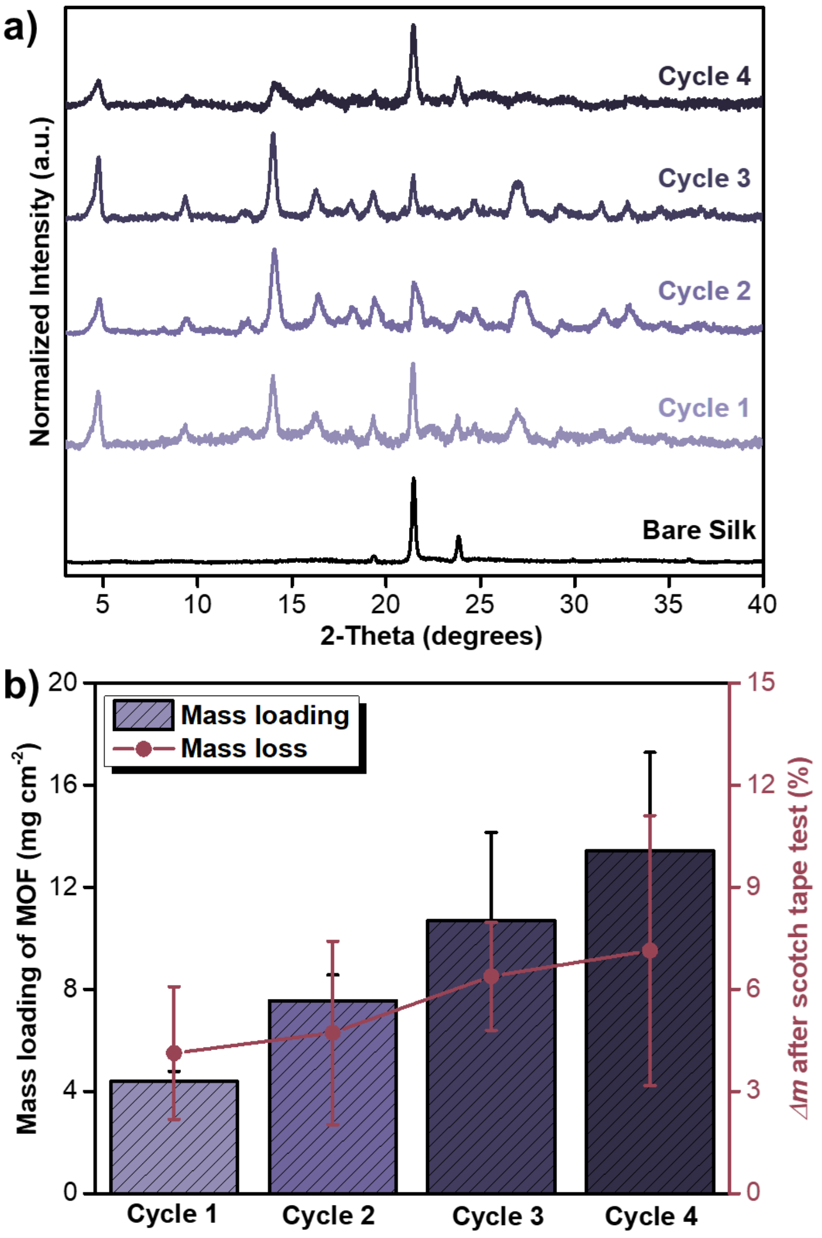

Figure S90. a) PXRD patterns and b) mass loading and loss of Co-HHTP@silk textile after multiple solvothermal deposition cycles determined by scotch tape test.

9.3 Characterization of Co-HHTP on textiles

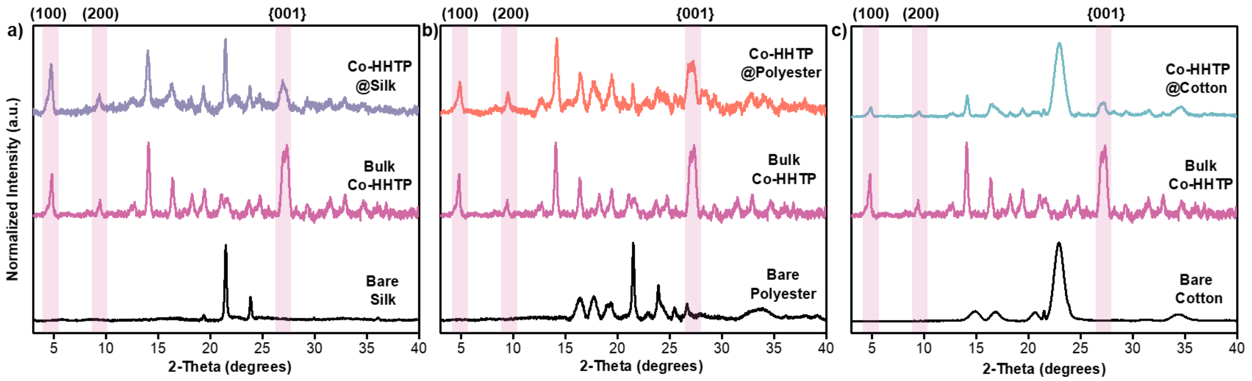

Figure S91. PXRD patterns of (bottom to top) a) bare silk, bulk Co-HHTP, Co-HHTP@Silk, b) bare polyester, bulk Co-HHTP, Co-HHTP@Polyester, and c) bare cotton, bulk Co-HHTP, Co-HHTP@Cotton.

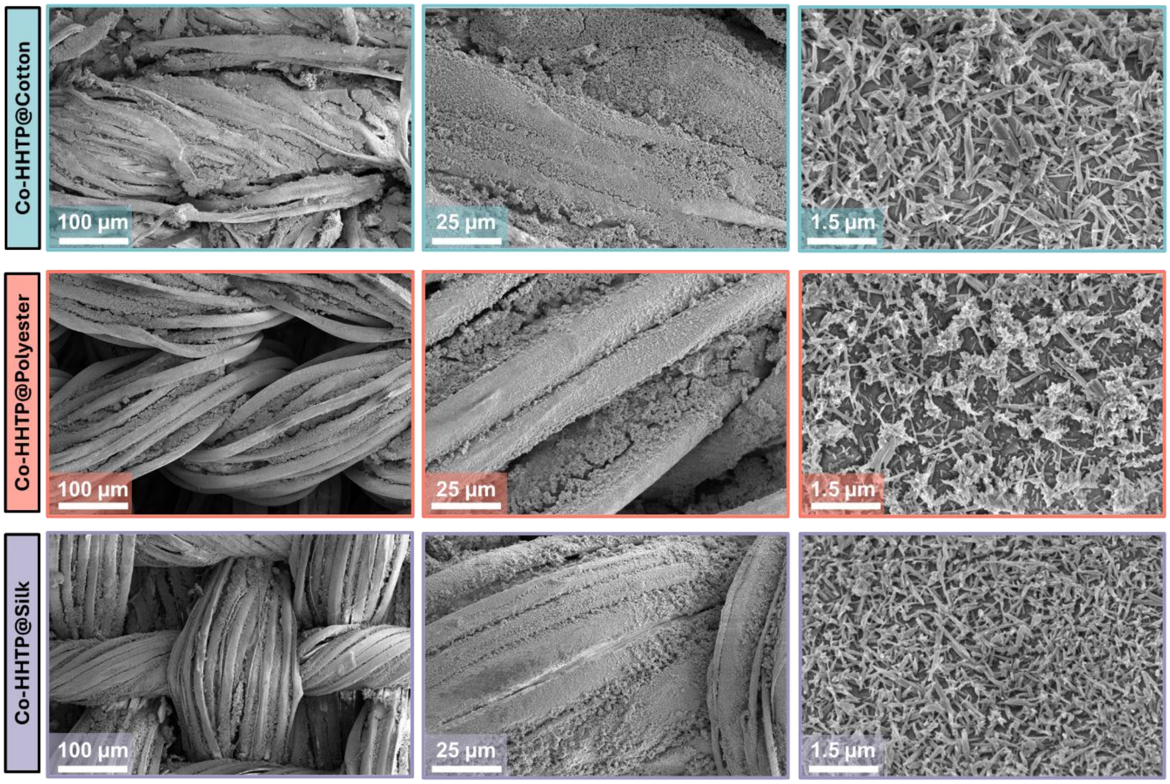

Figure S92. Additional SEM micrographs at different scales of Co-HHTP deposited on cotton, polyester, and silk swatches.

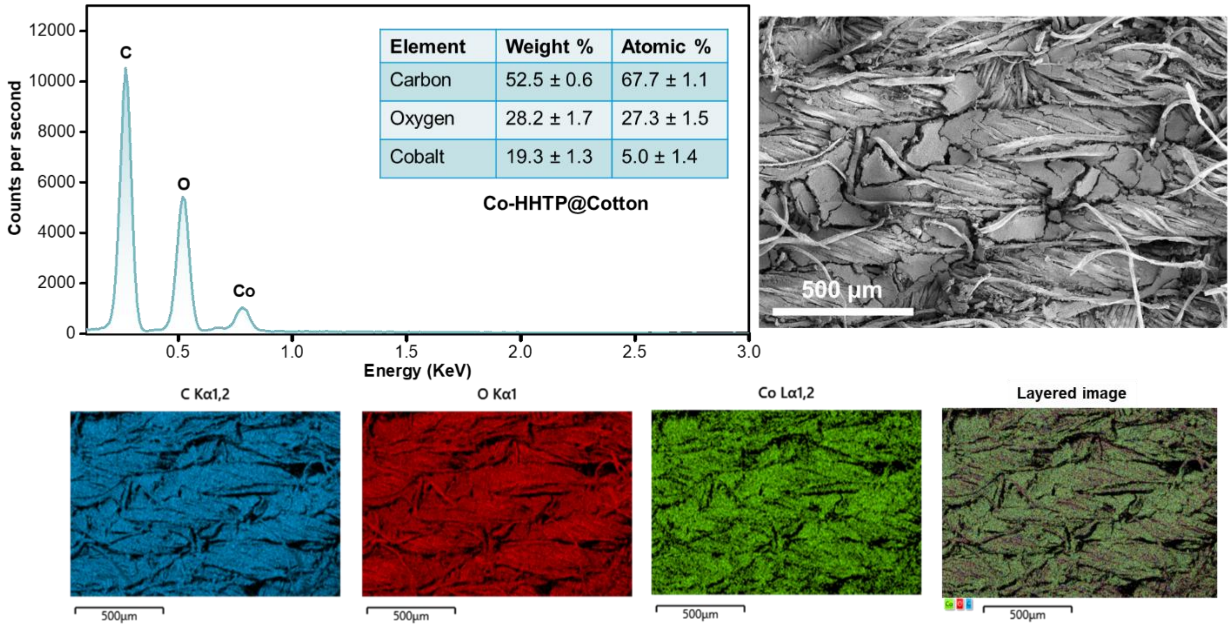

Figure S93. EDX spectrum and elemental mapping images of Co-HHTP@Cotton.

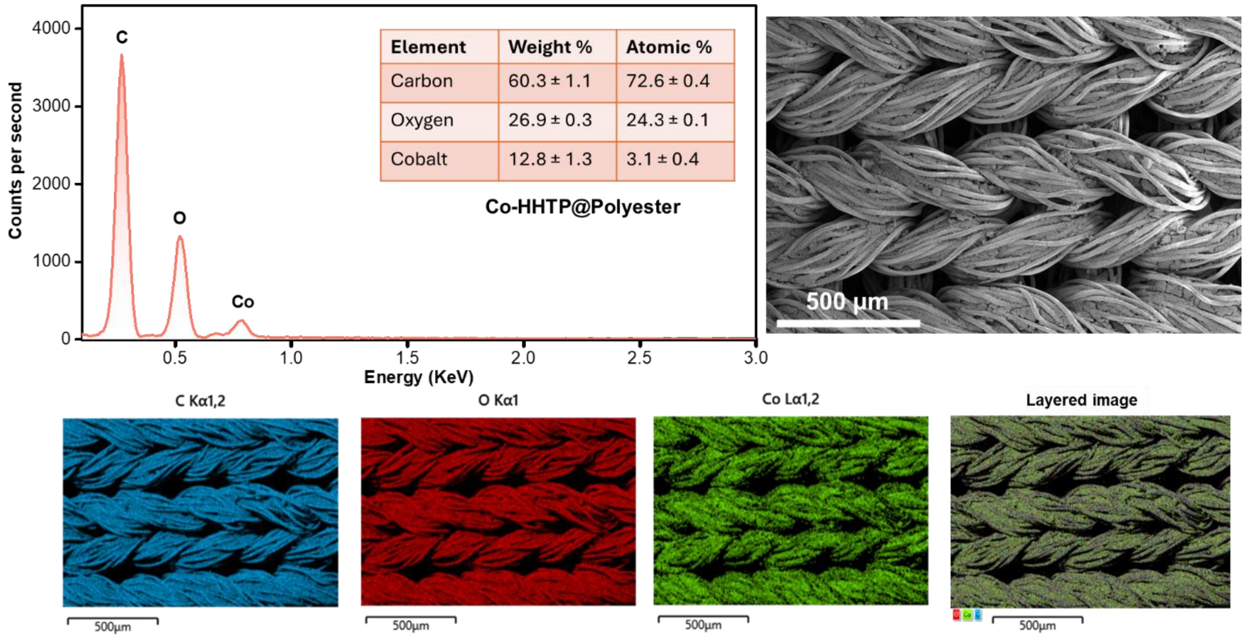

Figure S94. EDX spectrum and elemental mapping images of Co-HHTP@Polyester.

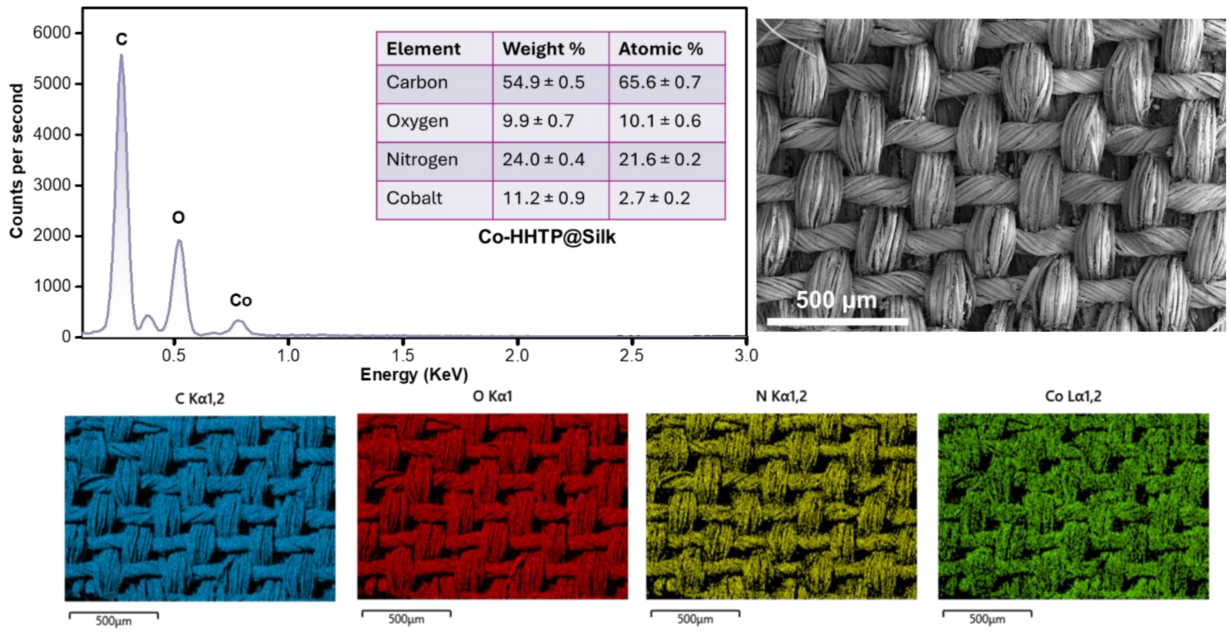

Figure S95. EDX spectrum and elemental mapping images of Co-HHTP@Silk.

9.4 Stability of Co-HHTP on textiles after adsorption

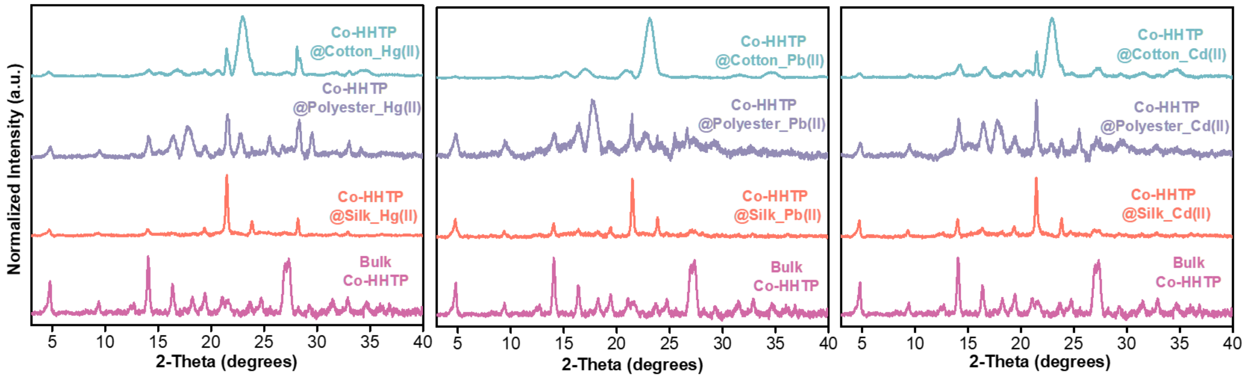

Figure S96. PXRD patterns of Co-HHTP@textiles following exposure to 50 ppm of Hg(II), Pb(II), and Cd(II) for 4 hours. The composite retained its crystallinity in most cases, in contrast to the bulk powder, owing to the strong interfacial adhesion between the MOF and the textile substrate, which enhances structural stability and mitigates framework degradation during heavy metal adsorption.

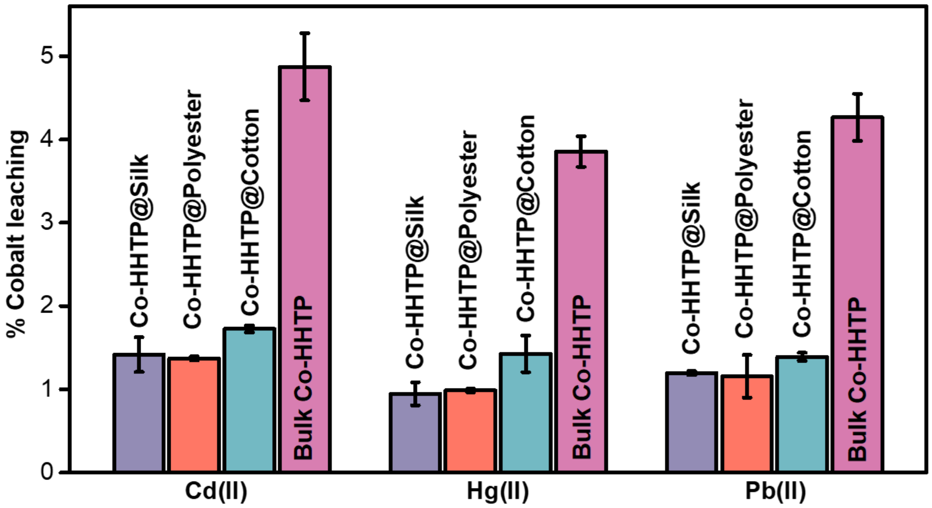

Figure S97. Percent (%) Cobalt leaching of bulk Co-HHTP and Co-HHTP@textiles following adsorption of 50 ppm of heavy metal pollutants for 4 hours.

10. Chemiresistive detection of heavy metals with Co-HHTP@textile

10.1 Fabrication of Co-HHTP@textile swatches

Co-HHTP@textile swatches used in the detection experiments were prepared via a layer-by-layer approach previously reported by our group.[9] In brief, 1 cm x 4 cm cotton swatches were first plasma-cleaned for 1 minute and sequentially washed with 30 mL of deionized (DI) water, ethanol, and acetone, then air-dried overnight. For Co-HHTP deposition, the swatches were immersed in 10 mL of a 300 mM aqueous solution of $Co(CH3COO)_2 \cdot 4H_2O$ for 1 min, then dried in an oven at 45 °C for 30 minutes. The swatches were then rinsedwith 10 mL of DI water and 10 mL of ethanol (1 minute each) followed by drying at 40 °C for 10 minutes. The resulting pale pink swatches were then immersed in 5 mL of a 1:1 DI water:ethanol solution containing 50 mM HHTP and 50 equivalents of NaOAc (relative to the ligand) for 1 min. After immersion, the swatches were vacuum-dried at 45 °C for 30 minutes, rinsed with DI water and ethanol, and dried again for 30 min, yielding black-colored swatches representing the first deposition cycle. This deposition cycle was repeated 8 times to ensure a uniform MOF growth. The resulting swatches were activated by soaking in DI water for 2 days, followed by solvent exchange with ethanol for another 2 days. Finally, the swatches were dried in a vacuum oven set at 72 °C for 18 hours before testing.

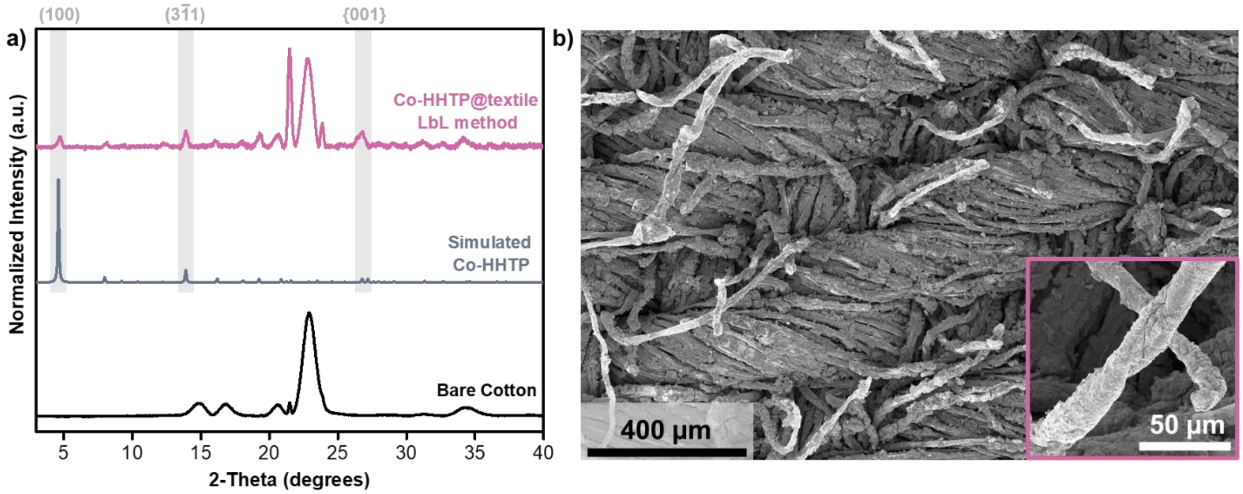

Figure S98. a) PXRD patterns and b) SEM micrographs of Co-HHTP@textile prepared via a layer-by-layer approach.

10.2 Chemiresistive sensing setup and methods

We used Co-HHTP@textile swatches as chemiresistors to detect Hg^{2+}, Cd^{2+}, and Pb^{2+} ions following the setup illustrated in **Figure S99**. In brief, a 0.5 x 4 cm^2 swatch was partially immersed in 7.5 mL of deionized (DI) water and connected at both ends using alligator clips secured with a clamp. These clips were linked to a Palmsens EmStat MUX8-R2 potentiostat (Palm Instruments, BV, Netherlands). After soaking the swatch for 10 minutes, we applied a driving voltage of 1.0 V across the textile, producing a baseline current ranging from 5 to 20 µA, depending on the swatch. We then allowed the system to equilibrate for an additional 30 minutes. Subsequently, we introduced 10 µL aliquots of heavy metal stock solutions (1-50 ppm) into the solution under continuous stirring at room temperature.

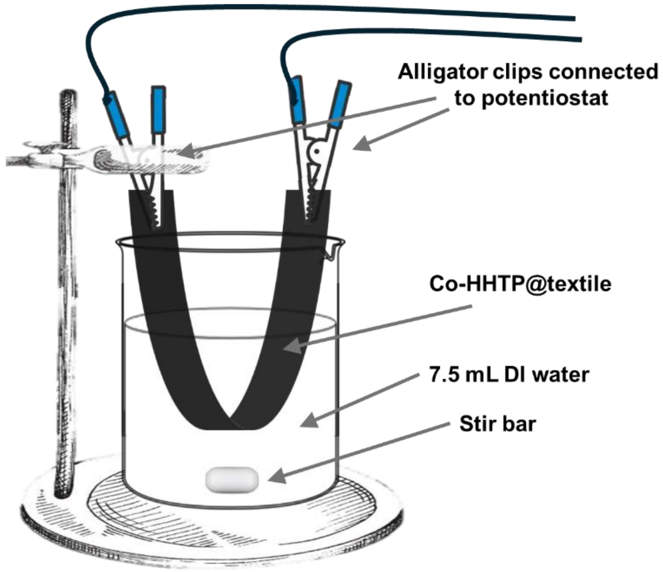

Figure S99. Schematic illustration of the experimental setup for the chemiresistive sensing of heavy metal cations using Co-HHTP@textile.

10.3 Replicates of chemiresistive detection experiments

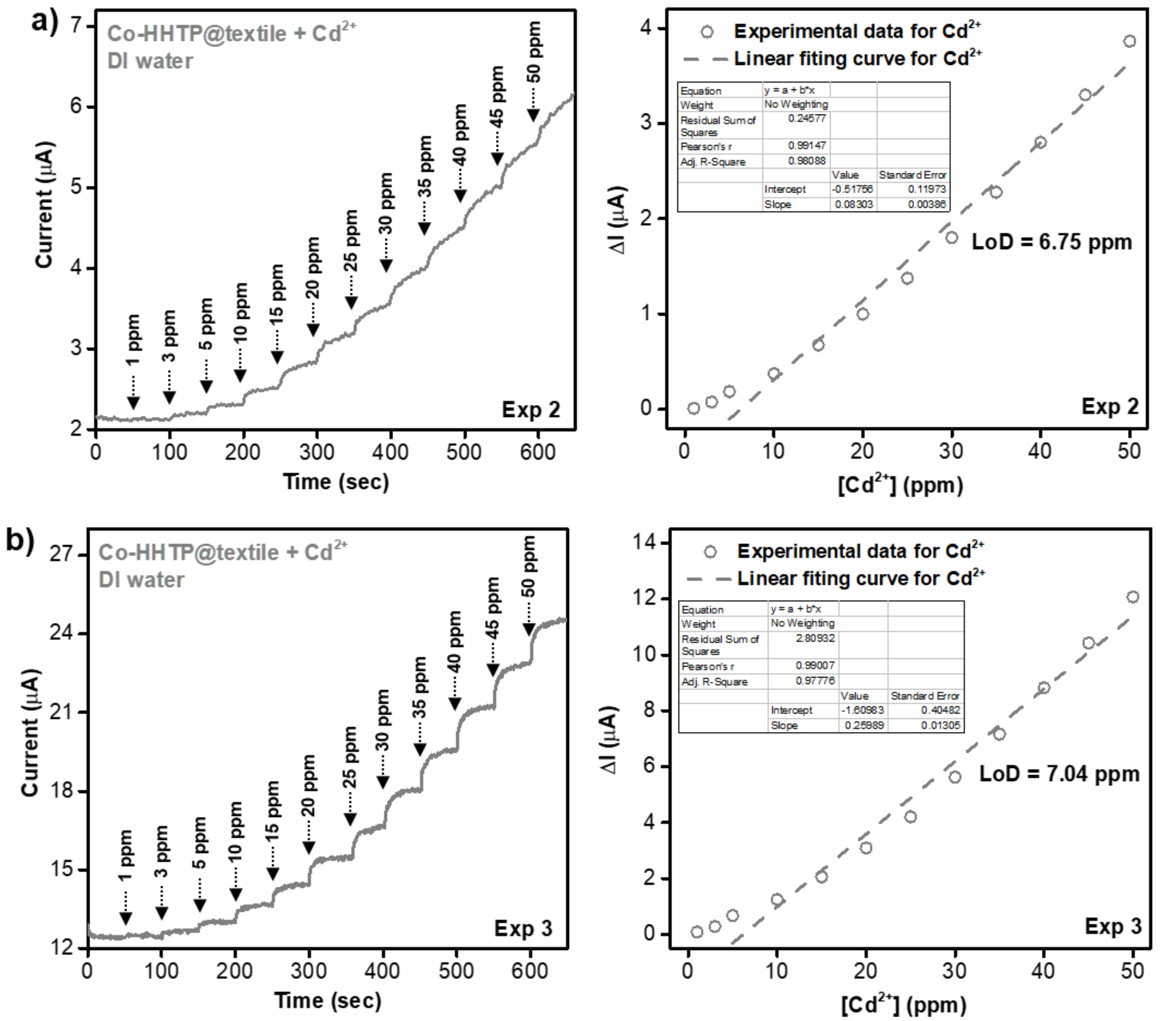

Figure S100. Additional replicates of chemiresistive detection of Cd^{2+} ions with 0.5 x 4 cm^2 of Co-HHTP@textile swatches. Change in current of textile swatches in a) Exp 2 and b) Exp 3 equilibrated in DI water at 1.0 V driving voltage to successive additions of Cd^{2+} ions in the concentration range of 1-50 ppm.

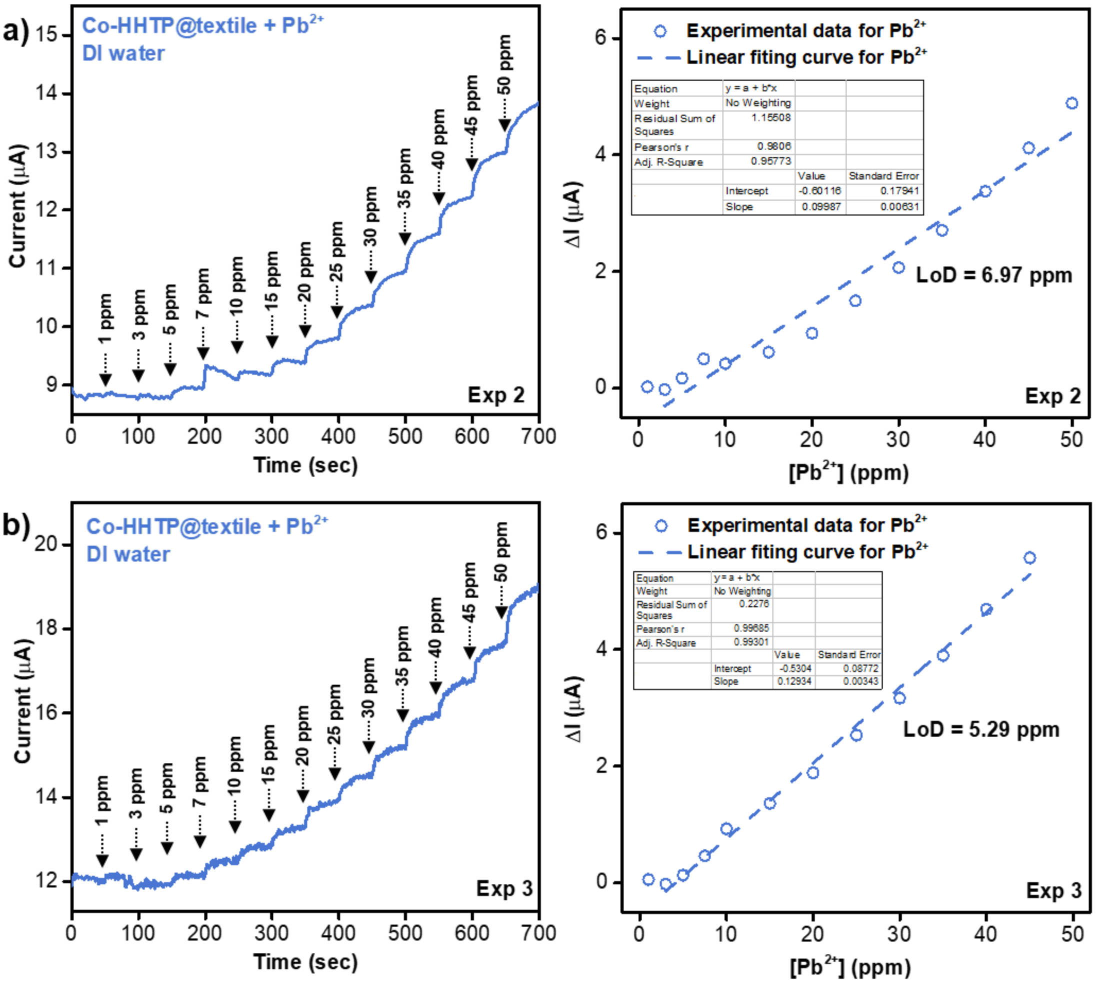

Figure S101. Additional replicates of chemiresistive detection of Pb^{2+} ions with 0.5 x 4 cm^2 of Co-HHTP@textile swatches. Change in current of textile swatches in a) Exp 2 and b) Exp 3, equilibrated in DI water at 1.0 V driving voltage to successive additions of Pb^{2+} ions in the concentration range of 1-50 ppm.

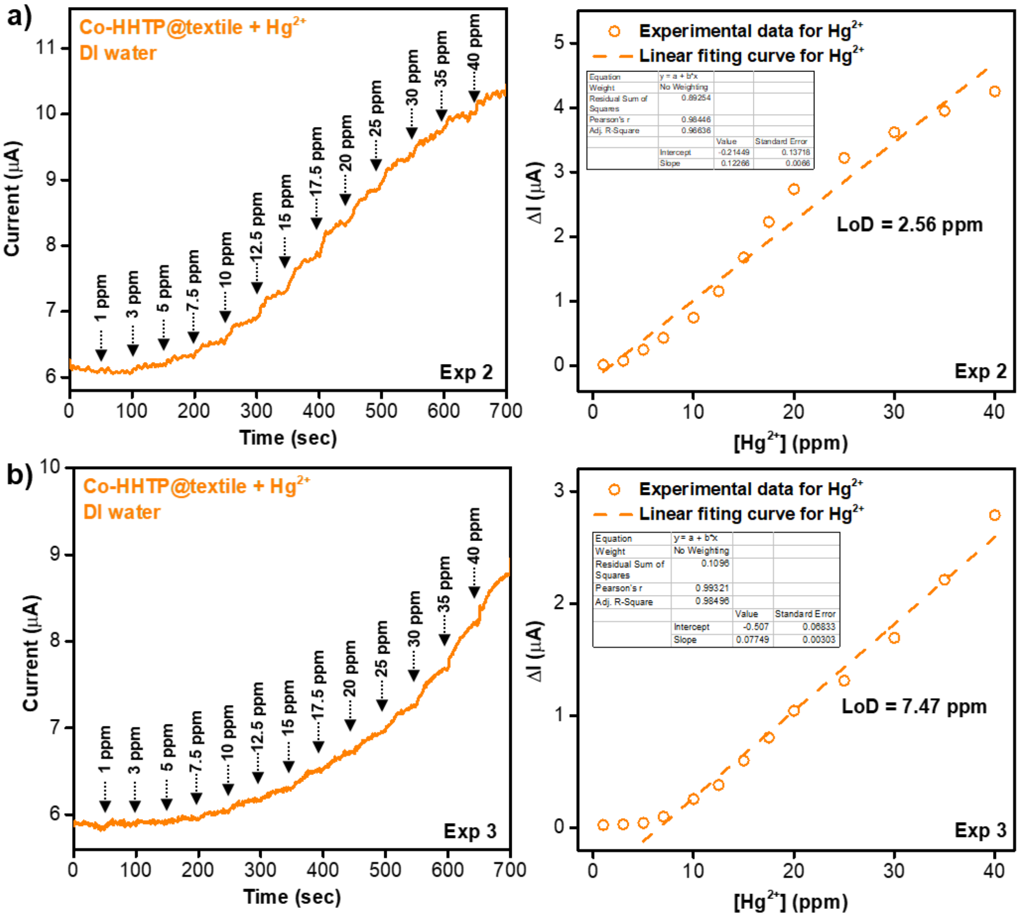

Figure S102. Additional replicates of chemiresistive detection of Hg^{2+} ions with 0.5 x 4 cm^2 of Co-HHTP@textile swatches. Change in current of textile swatches in a) Exp 2 and b) Exp 3, equilibrated in DI water at 1.0 V driving voltage to successive additions of Hg^{2+} ions in the concentration range of 1-40 ppm.

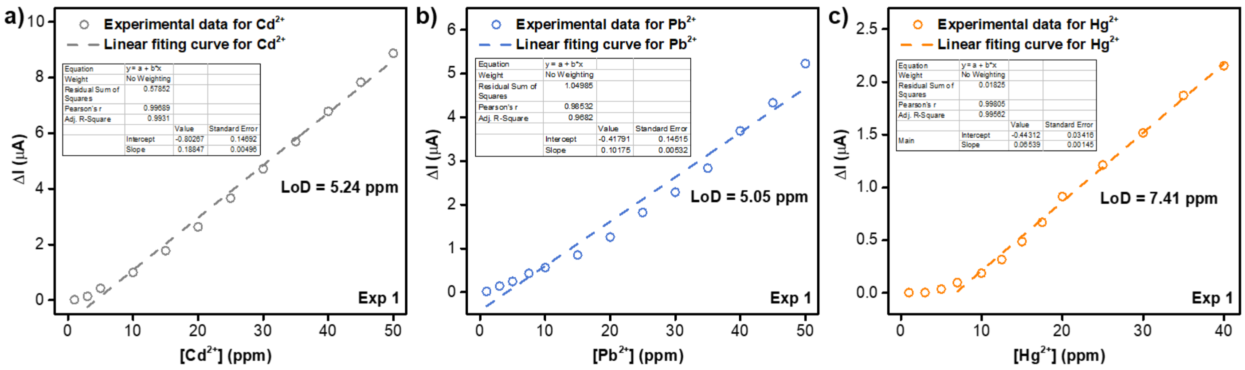

Figure S103. Response (change in current) vs concentration curves of Co-HHTP@textile swatches following exposure to a) Cd^{2+}, b) Pb^{2+}, and c) Hg^{2+} ions for the plots shown in Figure 7.

10.4 Estimation of the theoretical limit of detection (LoD)

The theoretical limit of detection (LOD) was determined from the signal-to-noise ratio (S/N)[28] as well as the slope and intercept of the linear straight line obtained from the concentration-dependent response plots according to **Equations S13 and S14**.

$$LOD = \frac{S/N - intercept}{slope} \quad \textbf{(Equation S13)}$$

$$S/N = 3 \times \sigma_{baseline} \quad \textbf{(Equation S14)}$$

where $\sigma_{baseline}$ is calculated as the standard deviation across the current values in the baseline (amperometric sensing experiment from 0 to 50 seconds).

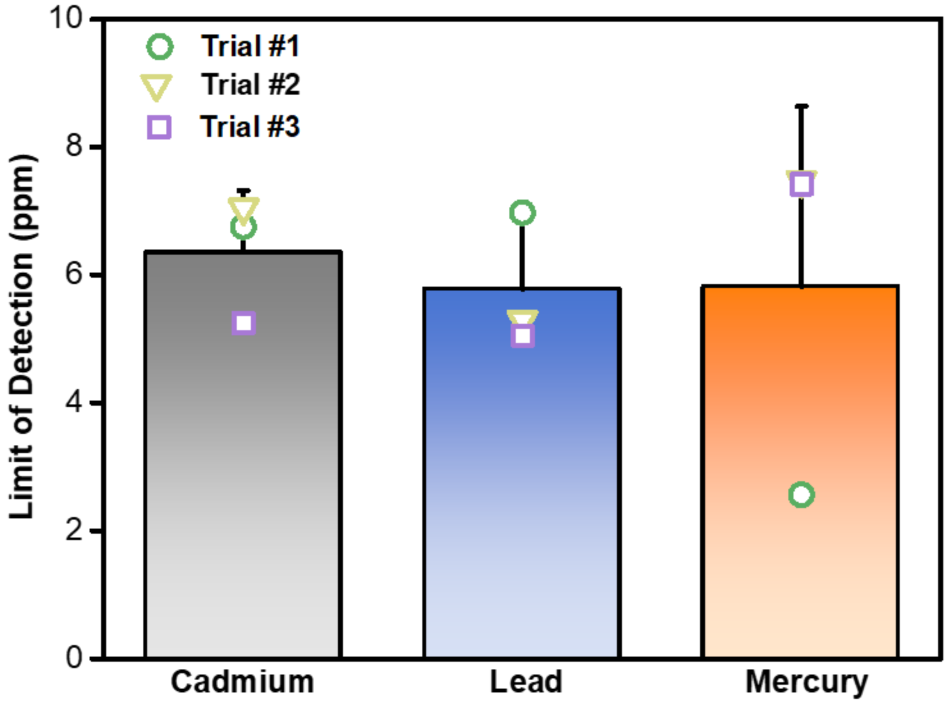

Figure S104. Calculated theoretical limit of detection (based on 3× S/N) for Co-HHTP@textile swatches in response to each analyte. Error bars represent the standard deviation of three replicates.

10.5 Comparison of multifunctional performance with literature

Sensing method	MOF	Cation	LoD	Q_{max}	Ref
**Fluorescence**	UiO-66-NH2@AuNCs/ZIF-8	Hg^{2+}	0.42 ppb	129.9 mg g^{-1}	29
	LMOF-263		3.3 ppb	380 mg g^{-1}	30
	NH2-MIL-53(Al)		0.15 μM	153.8 mg g^{-1}	31
	NH_2-UiO-66-SH (NSU66)		0.035 μM	265.3 mg g^{-1}	32
	TMU-48S		0.1 ppm	714 mg g^{-1}	33
	LMOF-263	Pb^{2+}	19.7 ppb	N/A	30
	NH_2-MIL-101(Fe)		5.2 nM	1.1 mM g^{-1}	34
	TMU-48S		N/M	454 mg g^{-1}	33
	FJI-H9	Cd^{2+}	10 ppm	286 mg g^{-1}	35
	Fe_3O_4/MOF/L-cysteine		0.94 ng mL^{-1}	248.2 mg g^{-1}	36
**Colorimetric**	Pt NP@UiO-66-NH_2	Hg^{2+}	0.35 nM	243.9 mg g^{-1}	37
	Ni-based MOF		N/M	713 mg g^{-1}	38
	Thioketone Al-MOFs Monitor		0.8 ppb	1110.5 mg g^{-1}	39
**Square Wave Anodic Stripping Voltammetry (SWASV)**	Zr-DMBD MOFs/3D-KSC	Hg^{2+}	0.05 μM	19.3 mg g^{-1}	40
**Amperometry**	Co-HHTP	Hg^{2+}	5.81 ppm	733 mg g^{-1}	**This work**
		Pb^{2+}	5.77 ppm	554 mg g^{-1}	
		Cd^{2+}	6.34 ppm	169 mg g^{-1}	

Table S13. Comparison of Q_{max} and LoD of Co-HHTP@textile towards the studied heavy metal cations with major reported MOFs used for dual sensing and capture of these contaminants.

10.6 Chemiresistive detection of heavy metals in the presence of interferences

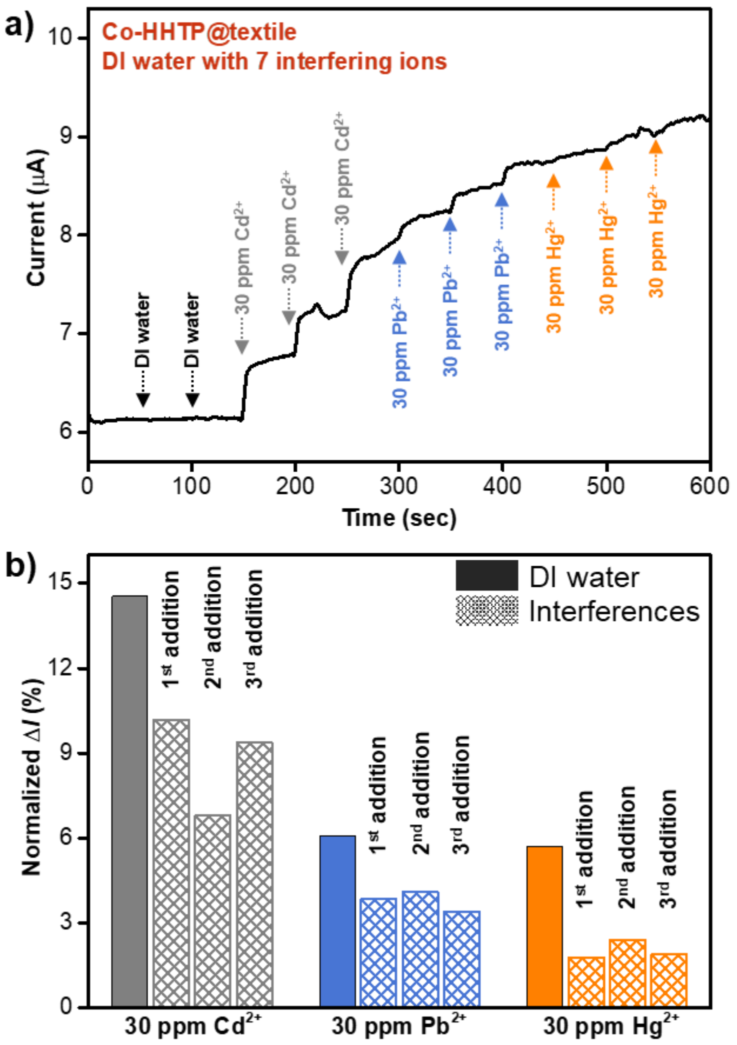

Figure S105. a) Chemiresistive detection of heavy metal cations with Co-HHTP@textile swatches in the presence of 30 ppm of each: sodium chloride, sodium formate, potassium carbonate, potassium sulfate, potassium phosphate, potassium nitrate, and potassium acetate. b) Comparison of the change in conductance of Co-HHTP@textile upon exposure to 30 ppm of the studied heavy metals in DI water with, and without the presence of interfering ions.

11. References

1. Swartzendruber, L. J., *Calculations for comparing two-point and four-point probe resistivity measurements on rectangular bar-shaped semiconductor samples*. US Department of Commerce, National Bureau of Standards: 1964; Vol. 13.

2. Sun, L.; Park, S. S.; Sheberla, D.; Dincă, M., Measuring and Reporting Electrical Conductivity in Metal–Organic Frameworks: Cd2(TTFTB) as a Case Study. *J. Am. Chem. Soc.* **2016,** *138* (44), 14772-14782.

3. Dubinin, M. M., Physical Adsorption of Gases and Vapors in Micropores. In *Progress in Surface and Membrane Science*, Cadenhead, D. A.; Danielli, J. F.; Rosenberg, M. D., Eds. Elsevier: 1975; Vol. 9, pp 1-70.
4. Puccia, V.; Avena, M. J., On the use of the Dubinin-Radushkevich equation to distinguish between physical and chemical adsorption at the solid-water interface. *Colloid and Interface Science Communications* **2021,** *41*, 100376.
5. Jrad, A.; Damacet, P.; Yaghi, Z.; Ahmad, M.; Hmadeh, M., Zr-Based Metal–Organic Framework Nanocrystals for Water Remediation. *ACS Appl. Nano Mater.* **2022,** *5* (8), 10795-10808.
6. Chen, T.; Dou, J.-H.; Yang, L.; Sun, C.; Libretto, N. J.; Skorupskii, G.; Miller, J. T.; Dincă, M., Continuous Electrical Conductivity Variation in M3(Hexaiminotriphenylene)2 (M = Co, Ni, Cu) MOF Alloys. *J. Am. Chem. Soc.* **2020,** *142* (28), 12367-12373.
7. Zhai, L.; Zheng, X.; Liu, M.; Wang, X.; Li, W.; Zhu, X.; Yuan, A.; Xu, Y.; Song, P., Tuning surface functionalizations of UiO-66 towards high adsorption capacity and selectivity eliminations for heavy metal ions. *Inorg. Chem. Commun.* **2023,** *154*, 110937.
8. Wang, Y.; Zhang, N.; Chen, D.; Ma, D.; Liu, G.; Zou, X.; Chen, Y.; Shu, R.; Song, Q.; Lv, W., Facile synthesis of acid-modified UiO-66 to enhance the removal of Cr(VI) from aqueous solutions. *Sci. Total Environ.* **2019,** *682*, 118-127.
9. Damacet, P.; Chandra, P.; Ambrogi, E. K.; Noh, H.-J.; Asmus, E. L.; Shehayeb, E. O.; Barcaro, G.; Monti, S.; Mirica, K. A., Electronic Textiles Based on Conductive Metal–Organic Frameworks as Scavengers and Sensors of Toxic Oxyanions from Water. *J. Am. Chem. Soc.* **2025,** *147* (31), 27561-27575.
10. Falaise, C.; Volkringer, C.; Facqueur, J.; Bousquet, T.; Gasnot, L.; Loiseau, T., Capture of iodine in highly stable metal–organic frameworks: a systematic study. *Chem. Commun.* **2013,** *49* (87), 10320-10322.
11. Yin, N.; Wang, K.; Xia, Y. a.; Li, Z., Novel melamine modified metal-organic frameworks for remarkably high removal of heavy metal Pb (II). *Desalination* **2018,** *430*, 120-127.
12. Damacet, P.; Shehayeb, E. O.; Mirica, K. A., Controlling the Spatiotemporal Self-Organization of Stimuli-Responsive Nanocrystals under Out-of-Equilibrium Conditions. *J. Am. Chem. Soc.* **2025,** *147* (2), 1584-1594.
13. Mercado-Borrayo, B.; Schouwenaars, R.; Litter, M.; Montoya-Bautista, C.; Ramírez-Zamora, R., Metallurgical slag as an efficient and economical adsorbent of arsenic. In *Water reclamation and sustainability*, Elsevier: 2014; pp 95-114.
14. Wang, J.; Guo, X., Rethinking of the intraparticle diffusion adsorption kinetics model: Interpretation, solving methods and applications. *Chemosphere* **2022,** *309*, 136732.
15. Mubarak, A. S.; Salih, S. S.; Kadhom, M.; Ghosh, T. K., Competitive and non-competitive adsorption of Cd(II) and Pb(II) from aqueous solution using Zr-BADS metal organic frameworks. *Sustainable Chemistry for the Environment* **2025,** *9*, 100231.
16. Liu, C.; Zeng, S.; Yang, B.; Jia, F.; Song, S., Simultaneous removal of Hg2+, Pb2+ and Cd2+ from aqueous solutions on multifunctional MoS2. *J. Mol. Liq.* **2019,** *296*, 111987.
17. Liu, X.; Xu, X.; Dong, X.; Park, J., Competitive adsorption of heavy metal ions from aqueous solutions onto activated carbon and agricultural waste materials. *Pol. J. Environ. Stud* **2020,** *29* (1), 749-761.
18. Nimbalkar, M. N.; Bhat, B. R., Simultaneous adsorption of methylene blue and heavy metals from water using Zr-MOF having free carboxylic group. *Journal of Environmental Chemical Engineering* **2021,** *9* (5), 106216.
19. Boix, G.; Troyano, J.; Garzón-Tovar, L.; Camur, C.; Bermejo, N.; Yazdi, A.; Piella, J.; Bastus, N. G.; Puntes, V. F.; Imaz, I.; Maspoch, D., MOF-Beads Containing Inorganic Nanoparticles for the

Simultaneous Removal of Multiple Heavy Metals from Water. *ACS Appl. Mater. Interfaces.* **2020,** *12* (9), 10554-10562.
20. Hmadeh, M.; Lu, Z.; Liu, Z.; Gándara, F.; Furukawa, H.; Wan, S.; Augustyn, V.; Chang, R.; Liao, L.; Zhou, F.; Perre, E.; Ozolins, V.; Suenaga, K.; Duan, X.; Dunn, B.; Yamamto, Y.; Terasaki, O.; Yaghi, O. M., New Porous Crystals of Extended Metal-Catecholates. *Chem. Mater.* **2012,** *24* (18), 3511-3513.
21. Zhong, Z.; Damacet, P.; Sánchez-González, E.; Eagleton, A. M.; Vereshchuk, N.; Wongratanaphisan, R.; Anderson, J. T.; Goncalves, S.; Peterson, G. W.; Blount, B.; Monti, S.; Barcaro, G.; Ibarra, I. A.; Mirica, K. A., Scalable templated fabrication of Cu-based MOF on textiles for simultaneous sensing, filtration, and detoxification of SO2. *Chem* **2025,** *11*, 102580.
22. Gittins, J. W.; Balhatchet, C. J.; Fairclough, S. M.; Forse, A. C., Enhancing the energy storage performances of metal–organic frameworks by controlling microstructure. *Chem. Sci.* **2022,** *13* (32), 9210-9219.
23. Giannozzi, P.; Andreussi, O.; Brumme, T.; Bunau, O.; Nardelli, M. B.; Calandra, M.; Car, R.; Cavazzoni, C.; Ceresoli, D.; Cococcioni, M., Advanced capabilities for materials modelling with Quantum ESPRESSO. *J. Phys.: Condens. Matter* **2017,** *29* (46), 465901.
24. Perdew, J. P.; Burke, K.; Ernzerhof, M., Generalized gradient approximation made simple. *Phys. Rev. Lett.* **1996,** *77* (18), 3865.
25. Grimme, S.; Antony, J.; Ehrlich, S.; Krieg, H., A consistent and accurate ab initio parametrization of density functional dispersion correction (DFT-D) for the 94 elements H-Pu. *J. Chem. Phys.* **2010,** *132* (15).
26. Frisch, M. e.; Trucks, G.; Schlegel, H. B.; Scuseria, G.; Robb, M.; Cheeseman, J.; Scalmani, G.; Barone, V.; Petersson, G.; Nakatsuji, H., Gaussian 16. Gaussian, Inc. Wallingford, CT: 2016.
27. Stolz, R. M.; Kolln, A. F.; Rocha, B. C.; Brinks, A.; Eagleton, A. M.; Mendecki, L.; Vashisth, H.; Mirica, K. A., Epitaxial Self-Assembly of Interfaces of 2D Metal–Organic Frameworks for Electroanalytical Detection of Neurotransmitters. *ACS Nano* **2022,** *16* (9), 13869-13883.
28. Li, H.; Wu, Y.; Xu, Z.; Wang, Y., Controllable preparation of a Cu NCs@ Zn-MOF hybrid with dual emission induced by an ion exchange strategy for the detection of explosives. *ACS Sens.* **2024,** *9* (9), 4701-4710.
29. Bi, X.; Liu, X.; Luo, L.; Liu, S.; He, Y.; Zhang, L.; Li, L.; You, T., Isolation of Sensing Units and Adsorption Groups Based on MOF-on-MOF Hierarchical Structure for Both Highly Sensitive Detection and Removal of Hg2+. *Inorg. Chem.* **2024,** *63* (4), 2224-2233.
30. Rudd, N. D.; Wang, H.; Fuentes-Fernandez, E. M. A.; Teat, S. J.; Chen, F.; Hall, G.; Chabal, Y. J.; Li, J., Highly Efficient Luminescent Metal–Organic Framework for the Simultaneous Detection and Removal of Heavy Metals from Water. *ACS Appl. Mater. Interfaces.* **2016,** *8* (44), 30294-30303.
31. Zhang, L.; Wang, J.; Du, T.; Zhang, W.; Zhu, W.; Yang, C.; Yue, T.; Sun, J.; Li, T.; Wang, J., NH2-MIL-53(Al) Metal–Organic Framework as the Smart Platform for Simultaneous High-Performance Detection and Removal of Hg2+. *Inorg. Chem.* **2019,** *58* (19), 12573-12581.
32. Zhang, L.; Wang, J.; Wang, H.; Zhang, W.; Zhu, W.; Du, T.; Ni, Y.; Xie, X.; Sun, J.; Wang, J., Rational design of smart adsorbent equipped with a sensitive indicator via ligand exchange: A hierarchical porous mixed-ligand MOF for simultaneous removal and detection of Hg2+. *Nano Res.* **2021,** *14* (5), 1523-1532.
33. Esrafili, L.; Gharib, M.; Morsali, A., Selective detection and removal of mercury ions by dual-functionalized metal–organic frameworks: design-for-purpose. *New J. Chem.* **2019,** *43* (46), 18079-18091.

34. Lv, S.-W.; Liu, J.-M.; Li, C.-Y.; Zhao, N.; Wang, Z.-H.; Wang, S., A novel and universal metal-organic frameworks sensing platform for selective detection and efficient removal of heavy metal ions. *Chem. Eng. J.* **2019,** *375*, 122111.
35. Xue, H.; Chen, Q.; Jiang, F.; Yuan, D.; Lv, G.; Liang, L.; Liu, L.; Hong, M., A regenerative metal–organic framework for reversible uptake of Cd (II): from effective adsorption to in situ detection. *Chem. Sci.* **2016,** *7* (9), 5983-5988.
36. Fan, L.; Deng, M.; Lin, C.; Xu, C.; Liu, Y.; Shi, Z.; Wang, Y.; Xu, Z.; Li, L.; He, M., A multifunctional composite Fe 3 O 4/MOF/l-cysteine for removal, magnetic solid phase extraction and fluorescence sensing of Cd (ii). *RSC Adv.* **2018,** *8* (19), 10561-10572.
37. Li, H.; Liu, H.; Zhang, J.; Cheng, Y.; Zhang, C.; Fei, X.; Xian, Y., Platinum nanoparticle encapsulated metal–organic frameworks for colorimetric measurement and facile removal of mercury (II). *ACS Appl. Mater. Interfaces.* **2017,** *9* (46), 40716-40725.
38. Halder, S.; Mondal, J.; Ortega-Castro, J.; Frontera, A.; Roy, P., A Ni-based MOF for selective detection and removal of Hg 2+ in aqueous medium: a facile strategy. *Dalton Trans.* **2017,** *46* (6), 1943-1950.
39. Radwan, A.; El-Sewify, I. M.; Shahat, A.; Azzazy, H. M.; Khalil, M. M.; El-Shahat, M. F., Multiuse Al-MOF chemosensors for visual detection and removal of mercury ions in water and skin-whitening cosmetics. *ACS Sustain. Chem. Eng.* **2020,** *8* (40), 15097-15107.
40. Yang, H.; Peng, C.; Han, J.; Song, Y.; Wang, L., Three-dimensional macroporous Carbon/Zr-2, 5-dimercaptoterephthalic acid metal-organic frameworks nanocomposites for removal and detection of Hg (II). *Sensor Actuat B: Chem* **2020,** *320*, 128447.
